# *Pseudomonas aeruginosa* acyl-CoA dehydrogenases and structure-guided inversion of their substrate specificity

**DOI:** 10.1038/s41467-025-57532-z

**Published:** 2025-03-08

**Authors:** Meng Wang, Prasanthi Medarametla, Thales Kronenberger, Tomas Deingruber, Paul Brear, Wendy Figueroa, Pok-Man Ho, Thomas Krueger, James C. Pearce, Antti Poso, James G. Wakefield, David R. Spring, Martin Welch

**Affiliations:** 1 Department of Biochemistry, Tennis Court Road, Cambridge, UK; 2 School of Pharmacy, University of Eastern Finland, Kuopio, Finland; 3 Interfaculty Institute of Microbiology and Infection Medicine (IMIT), University of Tübingen, Tübingen, Germany; 4 Partner-site Tübingen, German Center for Infection Research (DZIF), Tübingen, Germany; 5 Yusuf Hamied Department of Chemistry, Lensfield Road, Cambridge, UK; 6 Living System Institute, Department of Biosciences, University of Exeter, Exeter, UK

**Keywords:** Enzymes, X-ray crystallography, Microbiology techniques

## Abstract

Fatty acids are a primary source of carbon for *Pseudomonas aeruginosa* (PA) in the airways of people with cystic fibrosis (CF). Here, we use tandem mass-tag proteomics to analyse the protein expression profile of a CF clinical isolate grown on different fatty acids. Two fatty acyl-CoA dehydrogenases (designated FadE1 and FadE2) are strongly induced during growth on fatty acids. FadE1 displays a strong preference for long-chain acyl-CoAs, whereas FadE2 exclusively utilizes medium-chain acyl-CoAs. Structural analysis of the enzymes enables us to identify residues comprising the substrate selectivity filter in each. Engineering these residues enables us to invert the substrate specificity of each enzyme. Mutants in *fadE1* displayed impaired virulence in an infection model, and decreased growth on long chain fatty acids. The unique features of the substrate binding pocket enable us to identify an inhibitor that is differentially active against FadE1 and FadE2.

## Abstract (web summary)

Wang et al show that the pathogen *Pseudomonas aeruginosa* employs two distinct acyl-CoA dehydrogenases for β-oxidation. They identify the substrate selectivity filter in each enzyme, and show that the enzymes are required for optimal virulence.

## Introduction

*Pseudomonas aeruginosa* is an opportunistic human pathogen commonly associated with causing infections in the airways of people with cystic fibrosis (CF)^1^. The reasons for this pulmonary predilection are still not entirely clear, although the abundance of surfactant in the airway environment has been proposed as a contributory factor^2,3^. Dipalmitoyl phosphatidylcholine (DPPC) comprises 70%-80% of the airway surfactant, and is a rich source of long-chain fatty acids^4^.

**Fig. 1:**
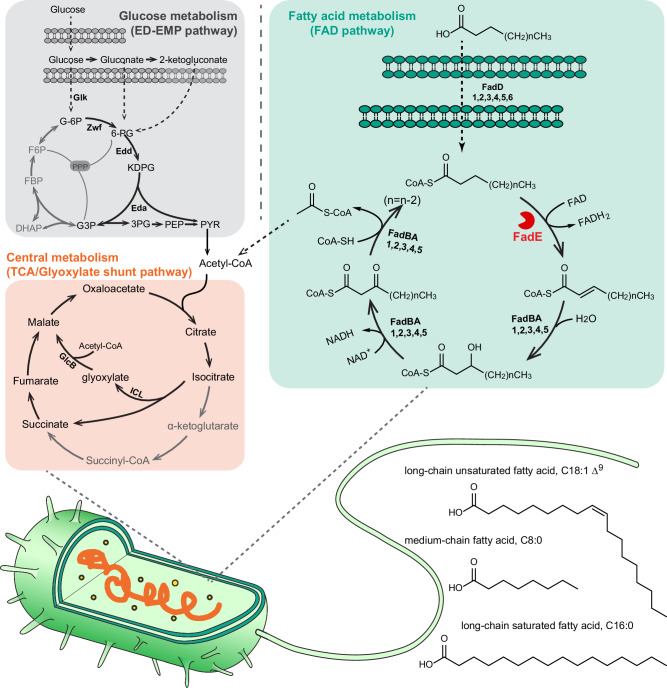
β-oxidation pathway and the fate of acetyl-CoA in *P. aeruginosa.* Acetyl-CoA is generated either via the oxidation of glucose in the Entner-Doudoroff pathway (grey panel) or from the β-oxidation of fatty acids (green panel; the reaction catalysed by FadE is highlighted). Irrespective of whether it is derived from glycolysis or β-oxidation, the acetyl-CoA then condenses with oxaloacetate to yield citrate, and thence, *iso*citrate (magnolia panel). In conditions that favour gluconeogenesis, some *iso*citrate is redirected away from the main tricarboxylic acid cycle (TCA cycle) reactions to enter the glyoxylate shunt, where it is cleaved by *iso*citrate lyase (ICL) to yield glyoxylate and succinate. The glyoxylate is then condensed with a further molecule of acetyl-CoA to yield malate and thence oxaloacetate for gluconeogenesis. This way, the four atoms of carbon entering the cycle as acetyl-CoA are conserved for biomass production.

Long-chain fatty acids are catabolized by the fatty acid degradation (Fad) pathway (Fig. 1). The Fad pathway has been best studied in *Escherichia coli*^5^. Here, exogenous fatty acids are transported across the cell envelope by FadL. The fatty acid is then esterified with coenzyme A to yield the corresponding acyl-CoA, in a reaction catalysed by FadD. The resulting acyl-CoA enters the β-oxidation cycle, being initially converted to enoyl-CoA by an FAD-dependent fatty acyl-CoA dehydrogenase (FadE), followed by sequential hydration, oxidation, and thiolytic cleavage reactions. The latter are carried out by the FadBA complex. However, and in spite of their physiological importance, the *E. coli* Fad enzymes have been the subject of remarkably little detailed biochemical analysis^6^. For example, we still understand very little about how the FADH_2_ moiety in bacterial acyl-CoA dehydrogenases becomes re-oxidized, or about how the enzyme manages to handle acyl-CoA substrates of different chain lengths. In this regard, purified *E. coli* FadE can desaturate both long- and medium-chain fatty acyl-CoA substrates, although since the latter fail to de-repress expression of the *fad* genes in this organism, *E. coli* does not grow on fatty acids shorter than around 14 carbon atoms^7^.

Whereas *E. coli* contains only a single set of *fad* genes, *P. aeruginosa* encodes six FadD and five FadBA homologues, all of which are known to contribute to fatty acid degradation^8–10^. However, the *P. aeruginosa* chromosome encodes more than 20 predicted acyl-CoA dehydrogenases (potential FadE enzymes), few of which have been genetically or biochemically characterized, and it is likely that most encode functions unrelated to β-oxidation. As a consequence, and although it is obvious that the organism is biochemically “wired up” to metabolize fatty acids, we know almost nothing about the FadE-catalysed step in *P. aeruginosa* β-oxidation (Fig. 1). Potentially, some insights into bacterial acyl-CoA dehydrogenase function have been obtained through analysis of fatty acid degradation in *Mycobacterium tuberculosis* (Mtb). Like *P. aeruginosa*, Mtb and the closely-related *M. smegmatis* (Ms) display a predilection for metabolizing fatty acids. Of the ca. 35 *fadE* homologues encoded by these mycobacteria, one protein, FadE5, was recently characterized in detail by Chen et al.^11^. They showed that FadE5 displays a rather broad substrate specificity, accommodating both long- and short-chain fatty acyl-CoAs in its active site (albeit with a very strong preference for long-chain substrates^11^). This raised the question of how such a wide range of substrates might fit into the active site of the enzyme. This conundrum was resolved by showing that the substrate binding cavity in FadE5_Ms_ is capable of remodelling (via re-orientation of certain amino acid side chains) to accommodate larger substrates, as needed. However, FadE5 appears to play a rather specialised role in mycobacterial metabolism (probably related to sulfolipid and polyacyltrehalose synthesis^12^, rather than β-oxidation) and was characterised in detail due to its involvement in drug resistance.

In this work, we identify the dominant fatty acyl-CoA dehydrogenases (designated FadE1 and FadE2, respectively) required for growth of *P. aeruginosa* on long- and medium-chain fatty acids. Deletion of the corresponding genes leads to impaired growth on these substrates, and to a virulence defect in an infection model. Kinetic analysis of the purified enzymes reveals that FadE1 exhibits a rather broad substrate specificity (like FadE5_Ms_), whereas FadE2 has a more restricted specificity profile. Structural analyses indicate that this specificity profile is likely determined by a selectivity filter in the substrate-binding pocket. Mutating the residues of this filter in FadE2 to the corresponding residues in FadE1 enabled the FadE2 to use long-chain fatty acyl-CoA substrates. Similarly, we were also able to invert the substrate specificity of FadE1 by mutating its substrate binding tunnel to become more FadE2-like. Finally, we show that the FadE1 and FadE2 enzymes can be differentially inhibited by low molecular weight compounds, opening the way towards targeting *P. aeruginosa* fatty acid catabolism for therapeutic effect.

## Results

### Proteomic analyses reveal that FadE1 and FadE2 are up-regulated during growth on fatty acids

**Fig. 2:**
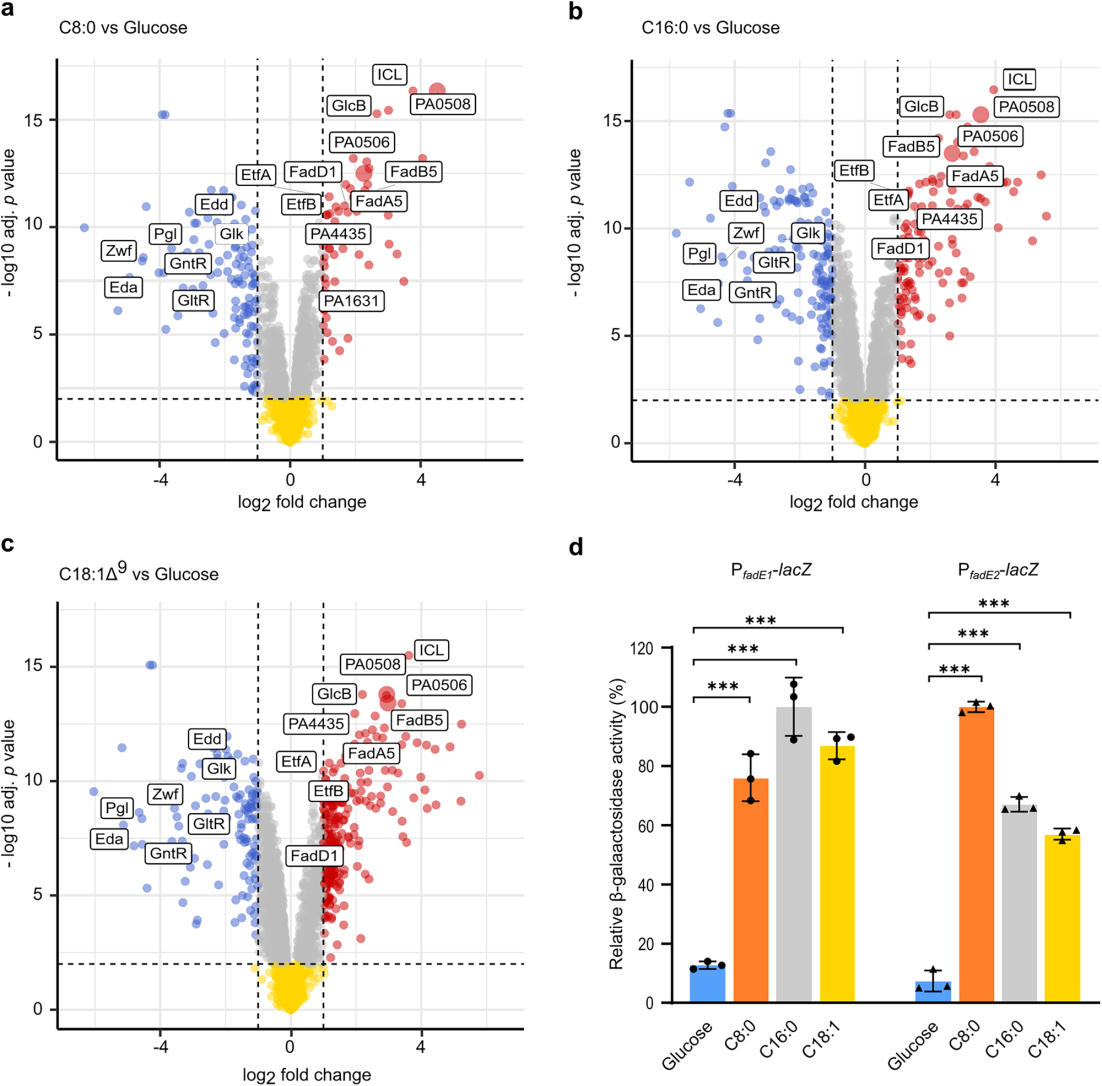
Proteomic analysis of PA grown on fatty acids or glucose. **a-c** Volcano plots showing differentially expressed PA proteins during growth on octanoate (C8:0), palmitate (C16:0), and oleate (C18:1^Δ9^). The plots show relative protein abundance (log_2_ abundance on fatty acid: abundance on glucose ratio) on the *x*-axis and significance level (-log_10_ *p*-value) on the *y*-axis. Red and blue points represent proteins with Benjamini-Hochberg adjusted *p* value  ≤ 0.01 and ≥ 2-fold change. Grey and yellow points indicate proteins below either the threshold 2-fold change or with an adjusted *p* > 0.01. *P* values were calculated with a moderated two-sided *t*-test using an empirical Bayesian method. The red circles indicate proteins that were up-regulated during growth on fatty acids by ≥ 2-fold (adjusted *p* ≤ 0.01). The blue circles indicate proteins that were down-regulated during growth on fatty acids by ≥ 2-fold (adjusted *p* ≤ 0.01). The grey circles indicate significantly-modulated proteins (adjusted *p* ≤ 0.01) that were up-regulated or down-regulated during growth on fatty acids by <2-fold. The yellow circles indicate proteins that were not significantly modulated (adjusted *p*  >  0.01). Proteins that are likely to be involved in glucose and fatty acid metabolism are boxed and labelled. The data points for PA0506 and PA0508 are enlarged in each plot. **d** β-galactosidase activity of PA14 containing pLP170 (P_*fadE1*_-*lacZ*) or pLP170 (P_*fadE2*_-*lacZ*), during growth on the indicated substrates. The relative β-galactosidase activity on each carbon source is shown (*cf*. the highest observed activity on that carbon source). Data represent the mean ± SD of three biological replicates. Statistical significance was determined by unpaired t-test, two-tailed, *** *p* < 0.01. Source data are provided as a Source Data file.

We reasoned that proteins involved in the Fad pathway would be up-regulated during growth on fatty acids. To identify potential FadE enzymes, we grew a clinical PA isolate (which whole genome sequencing revealed to be the Manchester Epidemic Strain^13,14^) on minimal media containing either glucose, octanoate (C8:0), palmitate (C16:0), or oleate (C18:1 ∆^9^) as a sole carbon source. We chose to work with the Manchester Epidemic Strain in this experiment because it displayed a comparable growth rate on all four carbon sources (Supplementary fig S1A), whereas many other clinical isolates and laboratory strains such as PAO1 exhibited differential growth or led to “clumping” on the long-chain fatty acid substrate. N = 4 independent biological replicates were analysed for each growth condition examined. Following harvesting of cells (at mid-log phase of growth) and protein extraction/tryptic digestion, the resulting peptides were derivatized with tandem mass tags (TMTs) and resolved/identified by LC-MS/MS. Of the 2640 proteins that were common to all of the samples, 19 showed increased abundance (log_2_FC ≥ 1.0, adjusted *p* value ≤ 0.01) on all three fatty acid substrates (*cf*. glucose) and 57 showed decreased abundance (Supplementary figs. S2a, b). A full list of the modulated proteins is shown in Supplementary Data 1. Not surprisingly, when PA was grown on fatty acid carbon sources, the abundances of proteins directly involved in glucose metabolism (e.g., the Entner-Doudoroff pathway proteins Glk, GntK, Zwf, Pgl, Edd, Eda, GnuT, GntR and GltR) were significantly diminished. However, the abundance of proteins such as FadA5, FadB5, GlcB (malate synthase G), and ICL (*iso*citrate lyase), which are responsible for fatty acid degradation and the downstream glyoxylate shunt^15–17^, were consistently elevated during growth on fatty acids. (Fig. 2). Notably, two potential fatty acyl-CoA dehydrogenases (PA0506 and PA0508), were also among the most highly up-regulated proteins on all three fatty acid substrates (Fig. 2a-c).

Hereafter, we designate PA0506 as FadE1, and PA0508 as FadE2. These two FadE homologues share 53% amino acid identity with one another, and share low sequence identity with *E. coli* FadE (FadE1 22.33% and FadE2 23.35%). We note that *fadE1* and *fadE2* are also both induced in CF sputum^3^. Reassuringly, bar-code Tn-seq implicated homologues of FadE1 and FadE2 (PP_0368 and PP_0370, respectively) in fatty acid catabolism by *Pseudomonas putida*, although no further biochemical or genetic investigation was carried out^18^. To confirm the induction of *fadE1* and *fadE2* expression during growth on fatty acids in vitro, we fused the upstream region of each gene with the promoterless *lacZ* on plasmid pLP170 to generate transcriptional fusions. The resulting reporter plasmids were introduced into PA14. We chose to work with PA14 because it is genetically-amenable, and of the domesticated laboratory strains, displayed the best growth on fatty acids in vitro. Growth of the reporter strains on fatty acids (*cf*. glucose) led to a substantial increase in β-galactosidase activity, indicating that expression from the *fadE1* and *fadE2* promoters is induced on these substrates (Fig. 2d).

### Mutations in *fadE1* and *fadE2* lead to impaired growth on fatty acids

**Fig. 3:**
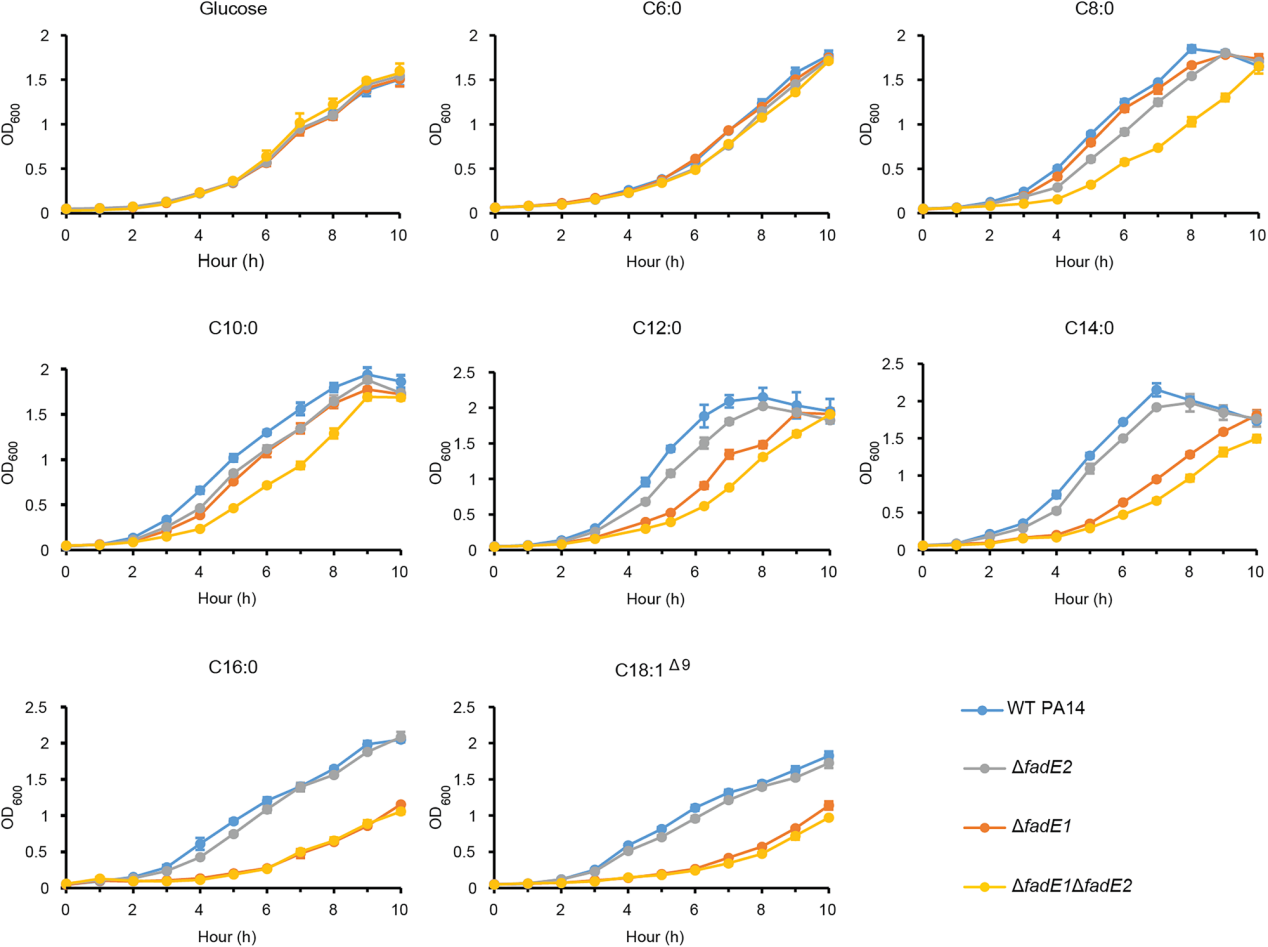
Growth of Δ*fadE1*, Δ*fadE2* and Δ*fadE1* Δ*fadE2* mutants (*cf*. wild-type PA14) on a range of fatty acids substrates with different acyl chain lengths. Each data point represents the mean ± SD of three biological replicates. Source data are provided as a Source Data file.

To investigate the role of *fadE1* and *fadE2* in fatty acid catabolism further, we generated defined deletion mutants in *P. aeruginosa* PA14. We then monitored the ability of these mutants and the wild-type progenitor to grow on fatty acids with chain lengths ranging from C6 to C18. None of the mutants (Δ*fadE1*, Δ*fadE2*, or Δ*fadE1* Δ*fadE2*) showed a growth phenotype relative to the wild-type on glucose or hexanoic acid (C6) as a sole carbon source (Fig. 3). However, the Δ*fadE1* mutant (and the Δ*fadE1* Δ*fadE2* double mutant) showed impaired growth on long-chain fatty acid substrates containing ≥ 12 carbon atoms. By contrast, the Δ*fadE2* mutant (and the Δ*fadE1* Δ*fadE2* mutant) showed growth impairment - albeit to a lesser extent - on medium-chain fatty acids containing ≤ 12 carbon atoms (Fig. 3). The growth phenotype of the *fadE2* mutant on C8 substrate was complemented by expression of *fadE2* in trans from pUCP20 (Supplementary Fig. S1b). Similarly, the growth phenotype of the *fadE1* mutant on C16 substrate was largely restored by expression of *fadE1* in trans (Supplementary Fig. S1c). Taken together, these data suggest that FadE1 plays a more important role in degrading long-chain fatty acids, whereas FadE2 plays a role in degrading medium-chain fatty acids. The residual growth on long-chain fatty acids in the Δ*fadE1* Δ*fadE2* double mutant suggests that additional (minor) acyl-CoA dehydrogenases remain to be characterized. In this regard, we note that several other FadE homologues (encoded by PA4435, PA1631, and PA5187) were also up-regulated during growth on fatty acids, albeit to a much lesser extent than FadE1 or FadE2 (Supplementary data 1).

### Mutants in *fadE1* show impaired virulence in vivo

**Fig. 4:**
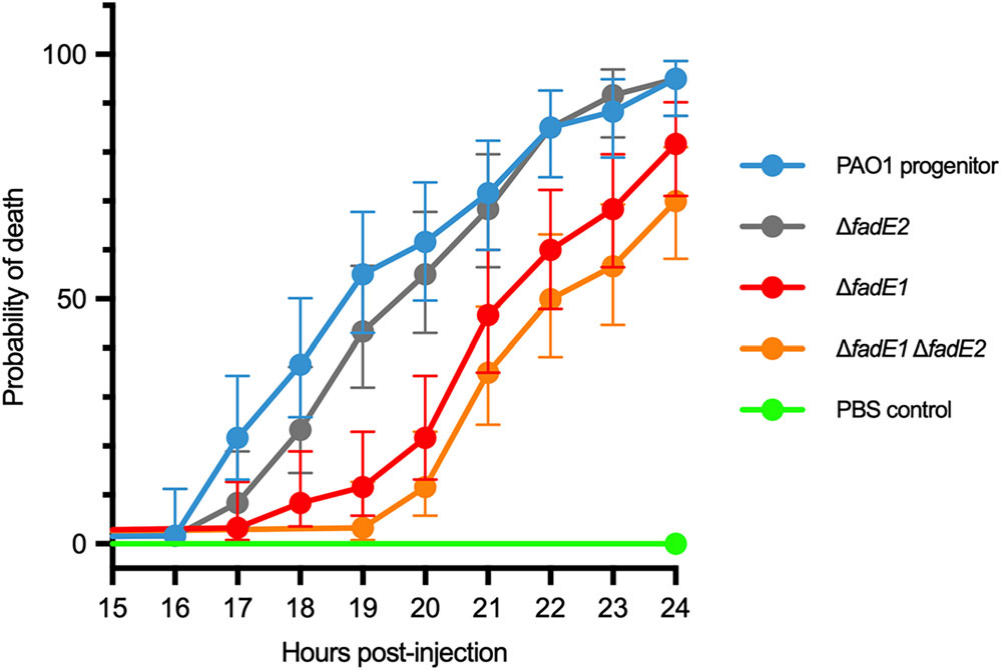
Δ*fadE1* and Δ*fadE1* Δ*fadE2* mutants display impaired virulence in a *Gall*eria *mellonella* infection model. Aliquots (20 CFU per injection) of the indicated strains, or, as a control for injection-induced mortality, 10 μL sterile PBS, were injected into the right proleg on the 6^th^ abdominal segment of each larva. The larvae were incubated at 37°C in darkness and checked for survival by thigmostimulation at hourly intervals from 14-24 h post injection. Sample sizes were calculated using R package “LogrankPower” V1.0.0 to detect a 30% difference mortality rate when accounting for Bonferroni corrections in the four treatment groups. Survival data was analysed using the log-rank (Mantel-Cox) test in GraphPad Prism 10. *n* = 60 larvae were tested for each strain/condition. Each data point shows the mean probability of death (with 95% confidence interval) for each indicated strain/condition. Source data are provided as a Source Data file.

We speculated that, given its predilection for fatty acid consumption, mutants in *fadE1* and/or *fadE2* may display impaired virulence in vivo. To test this, we infected *Galleria mellonella* larvae with the PA14-derived Δ*fadE1*, Δ*fadE2*, and Δ*fadE1* Δ*fadE2* mutants, scoring for death over time. However, the PA14 background proved to be exceptionally virulent, killing all the larvae rapidly, even at low inoculation titres. We therefore reconstructed the mutants in a PAO1 background, which is known to be less aggressive than PA14. This revealed that the Δ*fadE1* Δ*fadE2* mutant (and to a slightly lesser extent, also the Δ*fadE1* mutant) displayed significantly impaired ability to kill the larvae (Fig. 4). This is consistent with the data in Fig. 3 showing that FadE1 plays the more dominant role in fatty acid oxidation compared with FadE2.

### Oligomeric status and substrate specificity of FadE1 and FadE2

To determine the substrate specificity of FadE1 and FadE2, the *fadE1* and *fadE2* ORFs were His_6_-tagged, over-expressed, and purified (Supplementary Fig. S3). The His_6_ tags were proteolytically removed, and the molecular mass of the resulting native proteins was assessed using analytical ultracentrifugation (AUC). This revealed that FadE1 and FadE2 are likely to be dimeric in solution (Supplementary Fig. S4). Based on the genetic data and growth/virulence phenotypes (Figs. 3, 4), we speculated that FadE1 would display a preference for long-chain fatty acyl-CoA substrates, whereas FadE2 would prefer medium-chain substrates. Consistent with this, purified FadE1 exhibited dehydrogenase activity against both long-chain (C16, C18) and medium-chain (C8, and to a low extent, also C6) substrates with a preference for the longer chain acyl-CoAs, whereas FadE2 was active against only the medium-chain substrates (Table 1).

**Table 1:** Michaelis-Menten kinetic parameters for substrates oxidized by FadE1 and FadE2

Substrate		FadE1			FadE2	
	*K*_M_(μM)	*k*_cat_ (min^−1^)*	*k*_cat_/*K*_M_ μM^−1^	*K*_M_ (μM)	*k*_cat_ (min^−1^)*	*k*_cat_/*K*_M_ μM^−1^
C18-CoA	11.71 ± 1.29	1057 ± 33	90.3	NA		
C16-CoA	16.36 ± 2.09	1218 ± 40	74.5	NA		
C8-CoA	12.29 ± 1.43	977 ± 33	79.5	21.59 ± 1.23	1038 ± 21	48.1
C6-CoA	90.51 ± 8.51	184 ± 8.0	2.0	14.6 ± 1.43	1043 ± 31	71.5
C4-CoA		ND		10.35 ± 1.06	885 ± 25	85.0

ND, not determined; NA, no activity; *calculated for the monomeric unit of each enzyme. Source data are provided as a Source Date file.

### Structure of FadE1 and FadE2

**Fig. 5:**
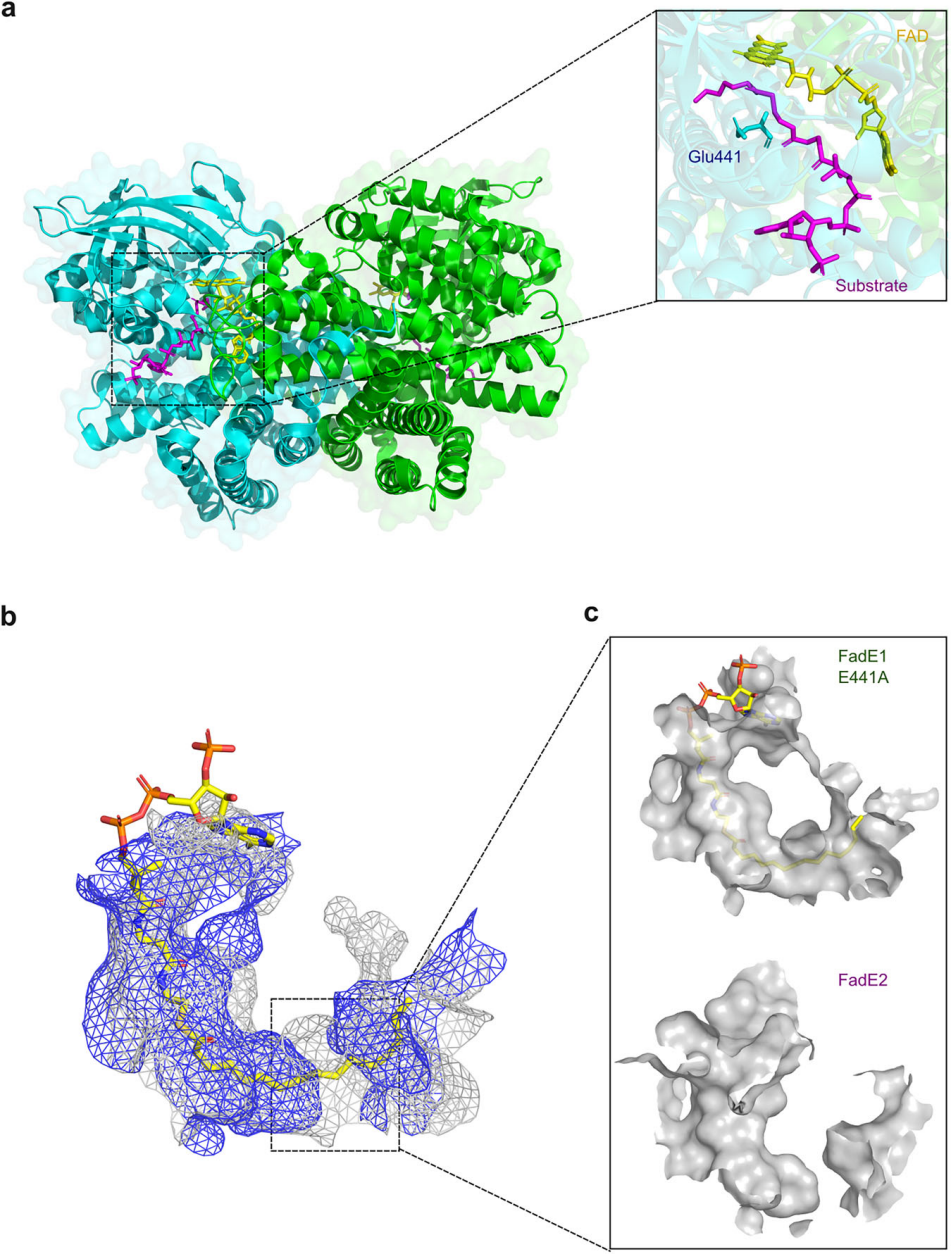
Overall structure of the FadE1 acyl-CoA dehydrogenase from PA and differences in the substrate-binding cavities of FadE1 and FadE2. **a** The cartoon representation shows the FadE1 homodimer with the individual protomers shaded green and cyan. The close-up view shows the C8-CoA substrate (magenta) sandwiched by the catalytic Glu441 side chain (cyan) and FAD cofactor (yellow) in the substrate binding pocket. **b** Superposition of the substrate-binding cavities in the mutant FadE1^E441A^ protein containing bound C16-CoA (grey) and apo-FadE2 (blue) shown as a mesh surface. The substrate, C16-CoA, in the FadE1^E441A^ structure is shown as yellow sticks. **c** Close-up view of the individual substrate-binding cavities in FadE1^E441A^ and FadE2.

To understand better why *P. aeruginosa* β-oxidation employs two acyl-CoA dehydrogenases, we solved the structures of FadE1 and FadE2 using X-ray crystallography. The structure of FadE1 and FadE2 were solved to a resolution of 2.08 Å and 1.94 Å, respectively (Supplementary table S2). Consistent with the AUC data, each asymmetric unit contained two homodimers arranged along a crystallographic 2-fold axis (Fig. 5a). In the structure of FadE1, we noted some additional electron density, which did not correspond to any crystallization additive, but was consistent with the presence of a bound fatty acyl-CoA moiety. The best-fit for this acyl-CoA moiety was octanoyl-CoA, presumably acquired from the *E. coli* host prior to purification. The acyl-CoA substrate was sandwiched between a probable catalytic residue (Glu441) and the FAD moiety (Fig. 5a).

FadE1 (601 residues) and FadE2 (592 residues) had the same overall fold (with the backbone RMSD of 1.001 Å) as other acyl-CoA dehydrogenase (ACAD) superfamily members. The first ca. 460 residues from both proteins comprised a pair of α-helix-rich domains (the N- and CI-domains) separated by a β-sheet domain (residues 160-280). The C-terminal ca. 140 residues of each protein formed a distinct α-helical bundle in which the helices are stacked perpendicular to those in the adjacent domains (Supplementary Fig. S5a). This additional structural element (domain CII) has been observed in the dimeric human very long-chain acyl-CoA dehydrogenase VLCAD (PDB 3B96)^19^, in 3-methylmercaptopropionate-CoA dehydrogenase (PDB 6IJC)^20^, and in the tetrameric *Caenorhabditis elegans* ACDH-11 (PDB 4Y9L)^21^. However, it is absent in porcine liver medium-chain acyl-CoA dehydrogenase (PDB 3MDD)^22^. One FAD molecule was bound in each protomer of FadE1 and FadE2.

### d_N_/d_S_ analyses

With the structural data in hand, and to identify possible functional hotspots in the proteins, we next looked for signatures of evolutionary selection in FadE1 and FadE2. Initially, this was done by aligning the amino acid sequences of FadE1 and FadE2 with the corresponding sequences extracted from the International *Pseudomonas* Consortium Database (IPCD). The IPCD comprises 854 *P. aeruginosa* sequences, including 379 CF-derived strains and 475 non-CF strains. The resulting % conservation data were then mapped onto the 3D structure of each protein. This revealed that the amino acid sequence of FadE1 is strongly conserved, irrespective of the source of the isolate (CF or non-CF), whereas FadE2 showed much less conservation across the whole sequence of the protein (Supplementary Fig. S6). To gain a finer-grained understanding of this, we carried out a residue-by-residue d_N_/d_S_ analysis across each protein. This revealed that, in spite of its strong signature of amino acid conservation, FadE1 displayed a markedly non-uniform distribution of d_N_/d_S_ values across its structure. Notably, the CII domain displayed a strong hallmark of negative (purifying) selection. Given that the corresponding CII domain in FadE2 did not display such a signal, these data suggest that the CII domain in FadE1 has probably reached a peak in the adaptive fitness landscape. By contrast, FadE2 displayed an overall signature of positive selection (dN/dS > 1), indicative of a drive towards differentiation away from the ancestral state. Neither the negative selection associated with FadE1, nor the positive selection associated with FadE2 appeared to be linked with the origin (CF- or non-CF) of the isolates.

The putative catalytic base in FadE1 and FadE2 (Glu441 and Glu442, respectively) is conserved (Supplementary Fig. S7). We therefore wondered whether we might improve substrate binding by preventing catalytic turnover. This was done by mutating the active site glutamate residues in FadE1 and FadE2 to alanine. This way, we were able to solve the structure of FadE1^E441A^ complexed with C16-CoA to a resolution of 1.44 Å (Supplementary table S1). However, efforts to solve the FadE2 structure with a bound fatty acyl-CoA ligand failed. Similar to FadE5_Ms_, we noted that in the presence of bound C16-CoA, the side chain of Phe289 in FadE1^E441A^ formed a π-π stacking interaction with the adenine ring on the CoA moiety of the substrate (Supplementary Fig. S5b). Overall, six FadE1^E441A^ side chains formed hydrogen bonds with the CoA moiety of the substrate, of which Arg454, Asp450 and Arg296 are also conserved in FadE5_Ms_ (Supplementary Fig. S5b). For FadE5_Ms_, it was previously reported that residues Tyr446, Met134, and Lys338 play an important role in defining the architecture of the active site. In particular, orientation of the Tyr446 side chain was postulated to act as a crucial gatekeeper, allowing the enzyme to bind substrates of differing acyl chain length. However, by comparing the substrate-bound FadE1^E441A^ structure with that of apo-FadE2 (with no substrate bound) the analogous residues (Tyr440 and Tyr441, respectively) adopt an exclusively “open” configuration (Supplementary Fig. S5c). In the structure of FadE5_Ms_, the tunnel that accommodates the acyl moiety is blocked mid-way along by the side chain of Met134; an obstruction that becomes displaced to allow long-chain fatty acyl-CoAs to bind. This is not the case in FadE1^E441A^, where the analogous residue is alanine (Supplementary Fig. S7), making this unplugging mechanism unlikely. Finally, Lys338 in FadE5_Ms_ is conserved in FadE2 but not in FadE1. Moreover, unlike the situation in FadE5_Ms_, where the loop containing Lys338 forms intimate contacts with the pantothenate moiety of the substrate, in both FadE1^E441A^ and FadE2, this loop points away from the substrate (Supplementary Fig. S5d). In summary, our data indicate that the features determining substrate specificity and binding in FadE1 and FadE2 are likely to be different to those in FadE5_Ms_.

### Molecular basis for the substrate specificity of FadE1 and FadE2

**Fig. 6:**
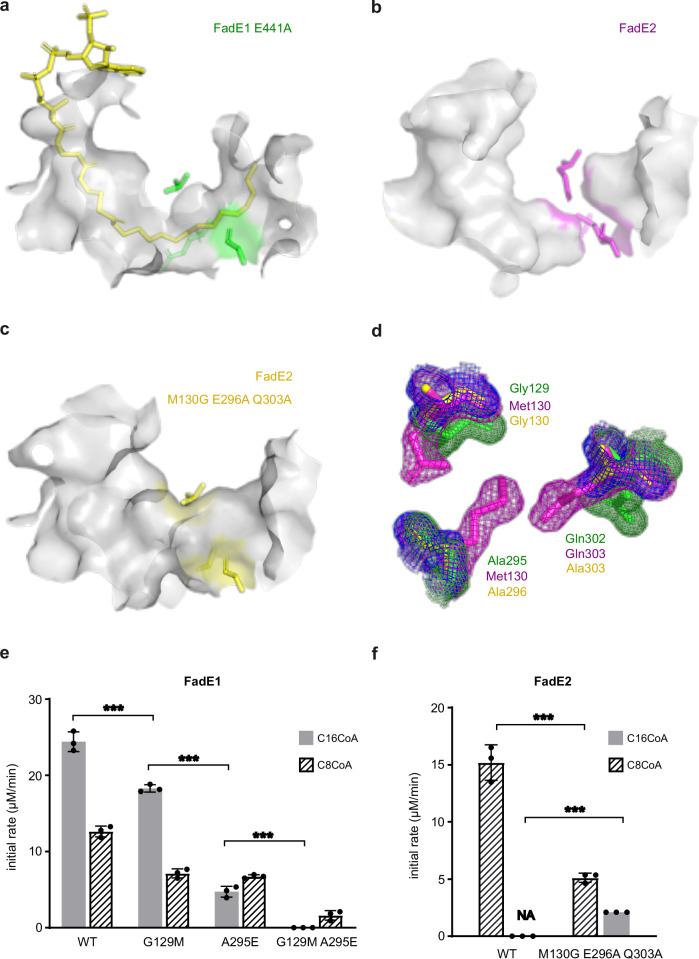
Engineering substrate specificity in FadE1 and FadE2. **a** The substrate-binding cavity in FadE1^E441A^. The bound C16-CoA ligand is shown in yellow, and residues Gly129 and Ala295 are shown in green. **b** The substrate-binding cavity in apo-FadE2. Residues Met130, Glu296, and Gln303 are shown in magenta. **c** Substrate-binding cavity in the FadE2^M130G E296A Q303A^ triple mutant. Residues Gly130, Ala296 and Ala303 are shown as yellow sticks. **d** 2F_c_-F_o_ map comparing key residues in the FadE1 (green sticks and mesh) and FadE2 (magenta sticks and mesh, contoured at 1σ) substrate tunnel pinchpoint with the analogous residues in the FadE2^M130G E296A Q303A^ triple mutant (yellow sticks and blue mesh, also contoured at 1σ). Enzymatic activity of wild-type and the indicated mutant FadE1 (**e**) and FadE2 (**f**) enzymes towards C16-CoA and C8-CoA. NA; no activity detected. The data in (**e** and **f**) show the mean ± SD of three technical replicates. Statistical significance was determined by unpaired t-test, two-tailed, *** *p* < 0.01. Source data are provided as a Source Data file.

The ability of fatty acyl-CoA dehydrogenases to catalyse substrate desaturation depends on the dimensions of the alkyl chain-binding pocket. To investigate this further, we superimposed the structures of C16-CoA-bound FadE1^E441A^ and apo-FadE2 to compare their substrate-binding cavities. This structural alignment revealed that the FadE1^E441A^ substrate cavity was deep enough to accommodate long-chain substrates. However, the substrate cavity of FadE2 was blocked in the middle, allowing FadE2 to only accommodate substrates with a maximum of 8-10 carbon atoms in the acyl chain (Fig. 5b, c). The blockage in the FadE2 substrate-binding cavity was due to the side chains of three amino acids; Met130, Glu296, and Gln303 (Fig. 6a–c). In FadE1, the corresponding residues are Gly129, Ala295, and Gln302. In the crystal structure of FadE2, the side chains of Met130 and Glu296 protrude directly into the substrate channel, creating a physical block. Similarly, and although it is conserved between FadE2 and FadE1, the glutamine residue (Gln303) in FadE2 is oriented towards the substrate channel, whereas in the C16-CoA-bound FadE1^E441A^ structure, the corresponding residue (Gln302) points away from the substrate-binding site.

In the light of the structural data, we predicted that mutating the FadE2 substrate tunnel to make it more “FadE1-like” by introducing the substitutions M130G, E296A, and Q303A, might lead to activity against C16-CoA. The Gln303→Ala substitution was introduced because, as noted above, in the FadE2 crystal structure the Gln303 side chain points towards the substrate binding tunnel. By introducing a shorter side chain (Ala), we hoped to offset any potential physical blockage in the tunnel arising from Gln303. Consistent with this, when we solved the X-ray structure of the FadE2^M130G E296A Q303A^ protein, the mutations had indeed opened up the substrate-binding tunnel (Figs. 6c, d; Supplementary Figs. S8a, b; Supplementary table S1). Furthermore, comparison of the distances between Cα atoms of residues 130 and 303 in wild-type FadE2 and in the FadE2^M130G E296A Q303A^ triple mutant over a 1 μs trajectory using molecular dynamics (MD) confirmed that the substrate binding tunnel was nearly 3 Å wider in the triple mutant (Supplementary Figs. S8a, b). To directly test whether these mutations could change the substrate specificity of the enzyme, we next measured the activity of the wild-type FadE2 enzyme and of the FadE2^M130G E296A Q303A^ triple mutant against C16-CoA and C8-CoA substrates. As previously noted, wild-type FadE2 was completely inactive against C16-CoA. However, the FadE2^M130G E296A Q303A^ mutant displayed low but non-negligible activity against this substrate (Fig. 6f). We conclude that by removing the “plug” in the substrate binding tunnel of FadE2, the enzyme can be engineered to accept long-chain acyl-CoA substrates.

We also used a similar approach to see if we could make the FadE1 substrate-binding site more “FadE2-like”, and increase its selectivity for C8-CoA over C16-CoA. We were unable to crystallize the relevant mutants, so instead, we employed in silico mutagenesis combined with MD simulations to gain structural insight. First, we mutated (in silico) Ala441 in the FadE1^E441A^ crystal structure back to glutamate, to generate a catalytically-active wild-type protein with bound C16-CoA (Supplementary Fig. S9a). Next, we used this template to generate a FadE1^G129M A295E^ double mutant in silico (Supplementary Fig. S9b). The wild-type and mutant structures were subjected to MD simulations (10 × 200 ns for each system) and metadynamics simulations (5 × 400 ns for each system). These analyses revealed that the acyl-chain binding pocket does indeed display a markedly reduced volume (from ca. 310 Å^3^ in the wild-type protein to ca. 240 Å^3^ in the FadE1^G129M A295E^ double mutant), irrespective of whether “normal” MD or metadynamic analyses were employed (Supplementary Fig. S9c).

To further experimentally test the MD predictions, we used site-directed mutagenesis to mutate the residues Gly129 and Ala295 in FadE1. The G129M mutation in FadE1^G129M^ decreased activity towards both the C8-CoA and C16-CoA substrates, but consistent with the MD predictions (that Met129 in this mutant spends relatively little time inserted into the substrate-binding tunnel) the mutant enzyme still retained higher activity towards the long-chain substrate (Fig. 6e). By contrast, introduction of the glutamate side chain in the FadE1^A295E^ mutant led to higher activity with the C8-CoA substrate compared with the C16-CoA substrate (Fig. 6e). When we combined the G129M and A295E mutations, the resulting FadE1^G129M A295E^ double mutant displayed only a low level of activity, but notably, had lost all activity against C16-CoA, while retaining detectable activity against C8-CoA (Fig. 6e). These data are consistent with the MD predictions (Supplementary Fig. S9).

### FadE1 as a target for drug development

**Fig. 7:**
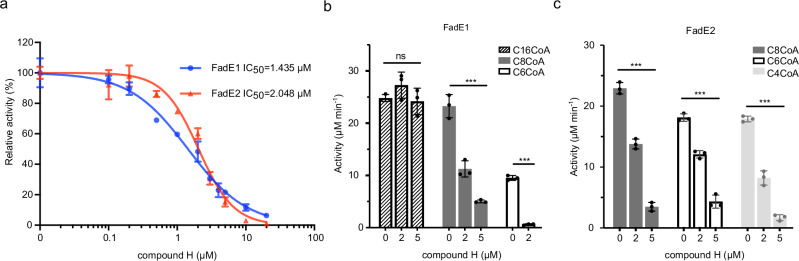
Inhibition of FadE1 and FadE2 by compound H. **a** Dose-response curve showing concentration dependent inhibition of purified FadE1 and FadE2 in vitro by compound H. The fatty acyl-CoA substrate was C8-CoA. **b** Concentration-dependent inhibitory activity of compound H against FadE1 with C16-CoA, C8-CoA, and C6-CoA substrates. **c** Concentration-dependent inhibitory activity of compound H against FadE2 with C8-CoA, C6-CoA, and C4-CoA substrates. The data in **b** and **c** show the mean ± SD of three technical replicates. Statistical significance within each group of substrate and enzyme were determined by either one-way ordinary ANOVA followed by Tukey’s multiple comparisons test, or an unpaired two-tail *t* test (for the FadE1 C6-CoA, where only two substrate concentrations were tested). ns, not significant within the indicated group. ***, *p* < 0.01. Source data are provided as a Source Data file.

Given the importance of fatty acid catabolism in CF infection scenarios, we wondered whether FadE1 and FadE2 might be good targets for drug development. To investigate this further, we “mutated” Ala441 back to Glu in the crystal structure of FadE1^Glu441Ala^/C16-CoA to generate a wild-type catalytic configuration in the active site, and then used the Schrödinger Drug Discovery suite to perform docking calculations on both the “wild-type” substrate-bound FadE1 and apo-FadE2. These analyses revealed that the fatty acid binding pocket in FadE1 and FadE2 both have a high druggable potential (Supplementary Fig. S10a). However, the active site in FadE1 had a much larger druggable pocket size, sufficient to incorporate small molecule ligands, even in the presence of bound fatty acyl-CoA substrate (Supplementary Fig. S10b). Given that a number of acyl-CoA dehydrogenase thioester inhibitors have been identified in previous studies (reviewed in ref. ^23^), we next investigated whether any of these compounds (Supplementary Fig. S11a) might differentially inhibit FadE1 *cf*. human very long-chain acyl-CoA dehydrogenase (HsVLCAD, PDB 3B96) and FadE2. The docking poses for each compound were energy minimized and the potential binding energy of the ligands was calculated using molecular mechanics with generalized Born and surface area continuum solvation (Supplementary Fig. S11b). This revealed a distinct binding profile for each ligand, with compounds E and H (Supplementary Figs. S11c, d) showing apparently greater specificity for FadE1. To investigate this further, we synthesized compounds E and H and tested their ability to inhibit FadE1 and FadE2 in vitro. For these assays, we employed the C8-CoA substrate, since this is a substrate for both enzymes (Table 1), enabling a direct comparison to be made. Compound E did not inhibit FadE1 or FadE2. However, compound H showed good inhibitory activity against both enzymes. Consistent with the computational predictions, compound H inhibited FadE1 slightly more efficiently (IC_50_ = 1.4 μM) than it did FadE2 (IC_50_ = 2.0 μM) (Fig. 7a). When we tested the efficacy of compound H in blocking activity against substrates with a range of acyl chain lengths, it had little effect on the ability of FadE1 to catalyse dehydrogenation of C16-CoA, but did decrease the activity of FadE1 against acyl-CoA substrates with chains of 8 carbon atoms or fewer (Fig. 7b). Compound H inhibited FadE2-catalysed substrate dehydrogenation of all of the substrates tested in a concentration-dependent manner (Fig. 7c).

### Phylogenetic relationship between FadE1, FadE2 and other fatty acyl-CoA dehydrogenases

To further investigate evolutionary relationships between the FadE-family of acyl-CoA dehydrogenases, we generated a phylogenetic tree from the available sequence data using maximum likelihood (Supplementary Fig. S12). The high branch support values (in most cases, marked by a Bayes posterior probability value of 1) suggest a clear separation between relevant clades. The sub-clade containing FadE1 and FadE2 (Supplementary Fig. S12) grouped closer to the clade containing the *M. tuberculosis* FadE5 enzyme, supporting the distinct druggability of these bacterial enzymes. Moreover, we note that the human HsVLCAD enzyme is located in clade that is distant from that of the *P. aeruginosa* and *M. tuberculosis* FadE enzymes, further reinforcing the notion that the bacterial enzymes might be selectively targeted for inhibition without affecting the host.

## Discussion

Prior to the current work, the main fatty acyl-CoA dehydrogenase(s) – FadE enzymes - used by *P. aeruginosa* in β-oxidation had not been identified. There are twenty-two putative acyl-CoA dehydrogenases encoded in the *P. aeruginosa* genome, so we used a proteomics-based approach to establish which of these are likely involved in β-oxidation. Our data show that PA employs two main fatty acyl-CoA dehydrogenases for β-oxidation, denoted here as FadE1 and FadE2. The FadE1 enzyme has a broad substrate specificity, being able to use both long- and medium-chain acyl-CoA substrates, whereas FadE2 exclusively uses medium-chain fatty acyl-CoAs. This raised several questions. For example, why does the organism employ two enzymes with overlapping, but distinct substrate specificities? Why not employ a single promiscuous enzyme with relaxed substrate specificity, like FadE5 in *M. tuberculosis*? The most likely explanation is that to achieve enough flux through β-oxidation to support rapid growth, the acyl-CoA dehydrogenases in *P. aeruginosa* need to be efficient. In this regard, we note that with C18-CoA as a substrate FadE1 exhibited a *k*_cat_/*K*_M_ value of 1.5 × 10^6^ M^-1^ s^-1^, whereas for FadE5_Mtb_, *k*_cat_/*K*_M_ for this substrate was around 250-fold lower (3.8 × 10^3^ M^-1^ s^-1^)^11^. Similarly, with C4-CoA as a substrate FadE2 exhibited a *k*_cat_/*K*_M_ value of 1.4 × 10^6^ M^-1^ s^-1^, whereas for FadE5_Mtb_, *k*_cat_/*K*_M_ for the C4-CoA substrate was nearly 500-fold lower (3.0 × 10^3^ M^-1^ s^-1^)^11^. We conclude that FadE1 and FadE2 are catalytically far more efficient than FadE5_Mtb_. However, the catalytic efficiency of FadE1 declined sharply when the substrate acyl-chain length was <8 carbon atoms, whereas FadE2 was optimally active on substrates of this size. Presumably, on long-chain fatty acyl-CoA substrates, FadE1 catalyses the initial rounds of β-oxidation, generating a pool of progressively shorter acyl-CoA intermediates. FadE2 is required to complete the β-oxidation of these trimmed-down acyl-CoAs. This would explain why FadE1 and FadE2 are both strongly induced during growth on long-chain fatty acids, although it does not explain why FadE1 is also strongly induced during growth on medium-chain fatty acids, since it should not be required for β-oxidation of these substrates. Presumably, the answer to this problem lies in how *fad* genes are regulated. Expression of the *fad* genes (including *fadE1* and *fadE2*) is known to be controlled by two transcriptional regulators, PsrA and PvrA. PsrA is a repressor of genes involved in long-chain fatty acid degradation, including PA0506 (*fadE1*) and *fadBA5*^24^. Consistent with this, a consensus PsrA binding motif (G/CAAACN_2–4_GTTTG/C) is located upstream (-136 to -123 bp) of the *fadE1* ORF. PsrA binds free fatty acids with acyl chains of >12 carbon atoms, leading to de-repression^24^. However, medium chain fatty acids such as octanoate do not bind to PsrA, so this does not explain why *fadE1* is strongly expressed during growth on this substrate. More recently, PvrA has been proposed as a transcriptional activator of genes involved in β-oxidation, including PA0508 (*fadE2*)^25^, and a PvrA binding motif (GGTCA) is also located upstream (-394 to -390 bp) of the *fadE1* ORF. PvrA is activated upon binding C16-CoA (although unfortunately, no other acyl-CoAs were investigated in that study). An economical working hypothesis is therefore that expression of *fadE1* and *fadE2* is stimulated by PvrA in the presence of medium-chain acyl-CoA molecules.

Based on phylogenetic analysis of the acyl-CoA dehydrogenases (Supplementary Fig. S12), FadE1 and FadE2 belong to the same sub-clade, along with another *fadE* homologue, PA0507. Notably, FadE1 (PA0506), PA0507, and FadE2 (PA0508) are encoded alongside one another, although the cluster is not predicted to be operonic. The protein encoded by PA0507 shares over 50% identity with FadE1 and FadE2 (Supplementary Fig. S13), and PA0507 retains the same constellation of residues around the substrate tunnel constriction (Gly128, Ala294, and Gln301 (PA0507 numbering)) as FadE1. However, and although peptides from PA0507 were detected in the proteomic analysis (indicating at least some expression of the protein) we observed no significant differential expression of the protein during growth on fatty acids. Furthermore, a deletion mutant in PA0507 had no phenotype when grown on C8, C16 and C18-chain fatty acids (Supplementary Figs. S13b-d). This suggests that if PA0507 plays a role in β-oxidation at all, it is likely to be a minor or specialised one.

Taken together, our data indicate that FadE1 and FadE2 have different substrate specificities, but that both are required to ensure optimal fatty acid degradation when the flux through β-oxidation is high, e.g., during growth in fatty acid-rich infection environments such as the CF airways or *Galleria mellonella* haemocoel (Fig. 4). Structural comparison of the two enzymes revealed residues that likely play an important role in determining substrate specificity, raising the question of whether these residues might be manipulated to make FadE2 more “FadE1-like” (i.e., accepting longer-chain substrates than the parent enzyme) and vice versa. Opening up the substrate-binding tunnel in FadE2 (by substituting Met130, Glu296 and Gln303 with smaller side chains) enabled the FadE2^M130G E296A Q303A^ mutant to utilize long chain fatty acyl-CoA substrate. Similarly, we were able to invert the substrate specificity preference of FadE1 from long acyl-chain to medium acyl-chain by increasing the bulk of side chains Gly129 and Ala295 in the FadE1^G129M Ala295E^ double mutant. However, in both cases, this bioengineering also led to lower overall enzyme activity. In the case of the FadE2^M130G E296A Q303A^ mutant, this is not surprising since the “blocked end” of the substrate-binding tunnel (after the original Met130/Glu296/Gln303 constriction) will presumably have been under no evolutionary selection pressure to remain accessible to a long chain substrate. On the other hand, this argument should not apply to the FadE1^G129M Ala295E^ mutant, since here, the block was engineered midway into a pre-existing tunnel. Presumably, the substrate tunnels in each enzyme are subtly different and should not be considered as simple hydrophobic tubes of different length. This makes good enzymological sense, since, as β-oxidation proceeds, the acyl-CoA substrates will become increasingly paired-down. Consequently, their interactions (and hence, their affinity) with the substrate-binding tunnel of FadE1 will diminish. If the function of FadE2 is indeed to “take over” from FadE1 at this point, it follows that the shortened substrates should bind to FadE2 more tightly than they do with FadE1. We would therefore expect the substrate binding tunnel of the two enzymes to be non-equivalent. Consistent with this, we found that a known acyl-CoA dehydrogenase inhibitor, compound H (Supplementary Fig. S11) showed marginally greater selectivity for FadE1 over FadE2 (Fig. 7a), consistent with computational predictions. Inhibition of FadE1 would be expected to have greater phenotypic impact, given that loss of *fadE1* had a much larger impact on growth than loss of *fadE2* (Fig. 3).

On one final note, we have shown that the CII domain in FadE1 displays a strong signature of negative selection, whereas the same domain in FadE2 does not. [Indeed, FadE2 displays all the hallmarks of an enzyme that is evolving away from the ancestral state.] Assuming that the CII domain is involved in membrane-association^19,26^, these data may indicate that the FadE1 and FadE2 proteins interact with different binding partners in the membrane. One logical suggestion for such binding partners might be the electron transfer flavoproteins (ETFs), which have been predicted (but not experimentally proven) to transfer reducing equivalents from the FADH_2_ moiety in FadE enzymes to the membrane-associated electron transport complex(es). In this regard, *P. aeruginosa* encodes two ETFs (*etfA* and *etfB*), both of which appear to be essential in the organism. However, the experimental evidence suggests that EtfA and EtfB are periplasmic, raising the question of how they capture reducing equivalents from FadE on the cytoplasmic face of the membrane. Current efforts are aimed at investigating this issue. A longer-term goal is to establish whether we can exploit the Fad pathway for therapeutic benefit, especially given that *P. aeruginosa* mutants defective in fatty acid oxidation are attenuated in both chronic and acute infection models^2^.

## Methods

### Strains, growth medium and culture conditions

*Pseudomonas aeruginosa* PA14 was used to generate the *fadE* mutants. The Manchester Epidemic Strain (MES), Pa10348, was used for the proteomic analyses. Pa10348 was isolated from a person with CF attending Papworth Hospital, Cambridge (UK)^13^. The whole genome sequence of Pa10348 was determined by MicrobesNG on the Illumina HiSeq 2500 platform and the data were deposited on the NCBI Bioproject database (Accession Number: BioProject PRJNA1008998). Fatty acid stock solutions (3% w/v of C6:0, C8:0, C10:0, C12:0, C14:0, C16:0 and C18:1∆^9^) were prepared in 1% Brij-58. KOH was added in a 1:1 mole ratio to convert the free acid to their potassium salt form^24^. Additional heating was required to emulsify the longer chain fatty acids and water before neutralization. Optical density (OD_600_) of the fatty acid cell culture was measured by a fourfold dilution with 4% Brij-58 in a cuvette^24^. For the proteomic and growth analyses, MOPS (morpholinepropanesulfonic acid) minimal medium^27^ supplemented with 0.1% (w/v) of the indicated fatty acid potassium salt or with 10 mM glucose was used, as required. Briefly, overnight starter cultures of *P. aeruginosa* were grown in 10 mL Luria Broth (LB) on a rotating drum at 37 °C. Cell pellets were then washed in the appropriate MOPS medium and inoculated to a starting OD_600_ of 0.05. The bacterial cultures were incubated with good aeration (agitation at 200 rpm) at 37 °C. When required, carbenicillin was added to *E. coli* cultures at a concentration of 50 μg mL^-1^ and to *P. aeruginosa* cultures at a concentration of 300 μg mL^-1^. For protein expression, chloramphenicol was added to the *E. coli* cultures at a concentration of 34 μg mL^-1^.

### β-galactosidase assays

Three samples of PA14 containing the indicated *lacZ* reporter plasmid were collected from independent cultures at OD_600_ 0.5 (corresponding to mid-exponential growth). The cells were lysed by sonication in 1 mL of 100 mM phosphate buffer (pH 7.2) supplemented with protease inhibitor cocktail (Roche). The cell lysate was clarified by centrifugation at 12,000 ×  *g* for 15 min at 4 °C. Aliquots (2 μL) of the supernatant were then added to 98 μL of buffer containing 100 mM potassium phosphate (pH 7.2) and 2 mM *o*-nitrophenyl-β-D-galactopyranoside (ONPG) substrate. Reactions were incubated at 37°C and terminated by addition of 100 μL 1 M Na_2_CO_3_. The amount of released nitrophenol was quantified using a FLUOstar Omega platereader (BMG labtech) at 420 nm. β-galactosidase activity was defined as the amount of released *o*-nitrophenol in one minute by one OD of cells^28^.

### Sample preparation for 16-plex TMT-quantitative proteomics

The proteomic analyses were carried out essentially as described by Dolan et al.^29^. Briefly, *n* = 4 cultures (100 mL volume) of the MES strain, Pa10348, were grown in MOPS-minimal medium supplemented with either glucose, octanoate, palmitate or oleate. The cultures were harvested at mid-exponential phase of growth by sedimentation at 3220  × *g* for 30 min at 4 °C. Cell pellets were resuspended in 1.5 mL lysis buffer (50 mM Tris-HCl, 100 mM NaCl, 1 mM *tris*(2-carboxyethyl)phosphine hydrochloride (TCEP) and 5% (v/v) glycerol, pH 7.5, containing one cOmplete Mini protease inhibitor cocktail (Roche) for every 10 mL of buffer) and lysed to completion by sonication on ice. The samples were clarified by sedimentation (21000 × *g*, 4 °C, 30 min) and their protein concentration was determined using the Bio-Rad protein assay (Bradford) reagent.

### TMT-labelling and high-pH first dimension reverse-phase fractionation

Samples containing 100 μg of protein were reduced (10 mM TCEP) and alkylated (17 mM iodoacetamide) and digested using trypsin. The resulting peptides were labelled with tandem mass tags (TMT) using a TMTpro™ 16-plex Label Reagent Set (Thermo Scientific, lot number LOT# WB314414) following the manufacturer’s instructions. The calculated N-terminal labelling efficiency was 92.2%. The percentage of TMT-labelled lysines was 99%. Table S3 indicates which samples were labelled with which tags.

The overall labelling efficiency was 99.6%. The samples were pooled and cleaned on Sep-Pak C18 cartridge, dried and dissolved in 20 mM ammonium formate (pH 10). The solution was then pipetted into a sample vial and placed into the autosampler of a Waters Acquity UPLC pump (Waters Corporation, Milford MA). The following LC conditions were used for the fractionation of the TMT samples: desalted peptides were resuspended in 0.1 mL 20 mM ammonium formate (pH 10.0) + 4% (v/v) acetonitrile. Peptides were loaded onto an Acquity bridged ethyl hybrid C18 UPLC column (Waters; 2.1 mm i.d. x 150 mm, 1.7 µm particle size), and profiled with a linear gradient of 5-60% acetonitrile + 20 mM ammonium formate (pH 10.0) over 60 min, at a flow-rate of 0.25 mL/min. Chromatographic performance was monitored by sampling eluate with a diode array detector (Acquity UPLC, Waters) scanning between wavelengths of 200 and 400 nm. Samples were collected in 1 min increments. The fractions were concatenated into 16 fractions (1  +  17, 2  +  18, … 16  +  32), and reduced to dryness by vacuum centrifugation.

### LC-MS/MS

Dried fractions were reconstituted in 0.1% formic acid and analysed by LC-MS/MS. LC-MS/MS experiments were performed using a Dionex Ultimate 3000 RSLC nanoUPLC (Thermo Fisher Scientific Inc, Waltham, MA, USA) system and a Lumos Orbitrap mass spectrometer (Thermo Fisher Scientific Inc, Waltham, MA, USA). Peptides were loaded onto a pre-column (Thermo Scientific PepMap 100 C18, 5 μm particle size, 100 Å pore size, 300 μm i.d. x 5 mm length) from the Ultimate 3000 auto-sampler with 0.1% formic acid for 3 min at a flow rate of 15 μL/min. After this period, the column valve was switched to allow elution of peptides from the pre-column onto the analytical column. Separation of peptides was performed by C18 reverse-phase chromatography at a flow rate of 300 nL/min using a Thermo Scientific reverse-phase nano Easy-spray column (Thermo Scientific PepMap C18, 2 μm particle size, 100 Å pore size, 75 μm i.d. x 50 cm length). Solvent A was water + 0.1% formic acid and solvent B was 80% acetonitrile, 20% water + 0.1% formic acid. The linear gradient employed was 2–40% B in 93 min. The total LC run run time was 120 min including a high organic wash step and column re-equilibration.

The eluted peptides from the C18 column LC eluant were sprayed into the mass spectrometer by means of an Easy-Spray source (Thermo Fisher Scientific Inc.). All m/z values of eluting peptide ions were measured in an Orbitrap mass analyser, set at a resolution of 120,000 and were scanned between m/z 380–1500 Da. Data dependent MS/MS scans (Top Speed) were employed to automatically isolate and fragment precursor ions by collision-induced dissociation (CID, Normalised Collision Energy (NCE): 35%) which were analysed in the linear ion trap. Singly charged ions and ions with unassigned charge states were excluded from being selected for MS/MS and a dynamic exclusion window of 70 s was employed. The top 10 most abundant fragment ions from each MS/MS event were then selected for a further stage of fragmentation by Synchronous Precursor Selection (SPS) MS3^30^ in the HCD high energy collision cell using HCD (High energy Collisional Dissociation, NCE: 55%). The m/z values and relative abundances of each reporter ion and all fragments (mass range from 100-500 Da) in each MS3 step were measured in the Orbitrap analyser, which was set at a resolution of 60,000. This was performed in cycles of 10 MS3 events before the Lumos instrument reverted to scanning the m/z ratios of the intact peptide ions and the cycle continued.

### Data Analysis

Proteome Discoverer v2.5 (Thermo Fisher Scientific) and Mascot (Matrix Science) v2.6 were used to process raw data files. Data were searched against the Uniprot *Pseudomonas aeruginosa*. PAO1 database (Supplementary Data 2) and with the common repository of contaminant proteins. Percolator was used to assess the FDR (false discovery rate) and only high-confidence peptides (FDR threshold 1%) of a minimum length of six amino acid residues were used for protein identification. Protein identification allowed an MS tolerance of ± 10 ppm and an MS/MS tolerance of ± 0.8 Da ppm along with permission of up to 2 missed tryptic cleavages. Quantification was achieved by calculating the sum of centroided reporter ions within a ± 2 millimass unit (mmu) window.

All comparative analyses were performed with the R statistical language^31^. The R package MSnbase^32^ was used for processing proteomics data. Briefly, this entailed missing value removal (instances where a protein was identified but not quantified in all channels were rejected from further analysis), log2-transformation of the raw data, followed by sample normalization; utilizing the ‘diff.median’ method in MSnbase (this translates all samples columns so that they all match the grand median). Protein differential abundance was evaluated using the Limma package^33^. Differences in protein abundances were statistically determined using Student’s t-test with variances moderated by Limma’s empirical Bayes method. P-values were adjusted for multiple testing by the Benjamini-Hochberg method^34^. The principal components analysis (PCA) plot, volcano plots, and Venn diagram were made in R v4.0.5. The code used to construct these plots is available at https://github.com/MWang2025/PAfadE.

### Plasmid constructions

PCR was done using Q5 polymerase (New England Biolabs). Recombinant plasmids for protein expression were made by inserting the full-length ORFs encoding *fadE1* and *fadE2* (PCR-amplified from PAO1 genomic DNA template and then digested with *BamH*I/*Nde*I) into *Nde*I/*BamH*I-digested pET19m. The encoded proteins have an N-terminal TEV protease-cleavable His_6_-tag. Site-directed mutagenesis of *fadE1* and *fadE2* was carried out by overlap extension PCR^35^. Pairs of overlapping primers were designed to introduce the specific mutation into the indicated ORF. In the first round of PCR, the overlap primers were paired with the cloning primers to amplify the 5’-fragment and 3’-fragment of the ORF. The two resultant DNA fragments (carrying the mutation site on their overlapping regions) were then fused and amplified using the cloning primers in a second round of PCR. The PCR product was then inserted to pET19m via *Nde*I and *BamH*I restriction sites. For complementation assays, the *fadE1* and *fadE2* ORFs, along with their endogenous ribosome binding site (RBS), were PCR-amplified. The amplicons were digested with either *Bam*HI/*Hind*III (for *fadE1*) or *Eco*RI/*Bam*HI (for *fadE2*) and cloned into appropriately digested pUCP20. For the *lacZ* transcriptional reporters, the promoter regions upstream of *fadE1* or *fadE2* were PCR-amplified and digested with *EcoR*I and *BamH*I. The amplicons were then introduced (separately) upstream of the promoterless *lacZ* ORF in appropriately-digested pLP170. All recombinant plasmids were confirmed by Sanger sequencing. A list of the primers used for this and the other constructs used in this paper is shown in Table S2.

### Protein expression and purification

*E. coli* Rosetta 2(DE3) was used as a host for protein expression. Overnight cultures (10 mL) were used to inoculate 1 L of LB medium supplemented with 10 μg mL^-1^ riboflavin, 50 μg mL^-1^ carbenicillin, and 34 μg mL^-1^ chloramphenicol. The cultures were grown at 37 °C until OD_600_ = 0.5. At that point, *iso*propyl-β-D-1-thiogalactopyranoside (IPTG) was added to a final concentration of 0.1 mM. The temperature was decreased to 20 °C, and the culture was grown overnight until harvesting. Cells were harvested by sedimentation (3000 × *g*, 10 min, 4 °C) and resuspended in 15 mL ice-cold buffer containing 50 mM Tris-HCl, 500 mM NaCl, 5% v/v glycerol (pH 7.5), and one cOmplete EDTA-free protease inhibitor cocktail tablet (Roche). The cell suspension was lysed by sonication. The crude lysate was supplemented with 200 μM FAD and incubated on ice for 30 min. The lysate was then clarified by sedimentation (17,500 × *g*, 30 min, 4 °C) and the clear supernatant was further filtered through 0.45 μm syringe filter. The sample was loaded onto a pre-equilibrated Ni-NTA column and washed with a large volume (200-300 column volumes) of 50 mM Tris-HCl, 500 mM NaCl, 5% v/v glycerol (pH 7.5). Bound protein was eluted in wash buffer supplemented with 250 mM imidazole. To cleave the His_6_-tag, the purified eluted protein was mixed with His_6_-tagged TEV protease (1:10 TEV:FadE) and dialysed at 4 °C for 36-48 h in 20 mM Tris-HCl, pH 7.5, 100 mM NaCl, 10% v/v glycerol, 10 mM imidazole (pH 7.5). The His_6_-TEV and liberted His_6_ tags were removed using Ni-NTA resin, and the unbound protein was dialysed to remove the imidazole. Purified protein was then concentrated by ultrafiltration, frozen in aliquots (in liquid nitrogen) and stored at -80 °C until use. The protein concentration was determined spectrophotometrically at λ = 280 nm, assuming ε_FadE1_ = 64330 M^-1^ cm^-1^ and ε_FadE2_ = 75665 M^-1^ cm^-1^. FadE1 E441A and FadE2 E442A were purified using the same procedure but without supplementation of the growth medium with riboflavin or addition of FAD to the lysis buffer.

### Analytical ultracentrifugation analyses

Purified FadE1 and FadE2 were dialysed overnight at 4 °C against 20 mM Tris-HCl, 100 mM NaCl, 10 mM imidazole (pH 7.5). The proteins were then diluted to 1 mg mL^-1^ for analytical ultracentrifugation, using the dialysate buffer as blank. The samples were sedimented at 50000 rpm with an AN-60 Ti rotor (Beckman Optima Xl-I) at 20 °C. Absorbance (A_250_) and interference scans were taken every 180 s. Data analysis was conducted with SEDFIT. The viscosity and density of the buffer used in the experiments were estimated by SEDNTERP.

### *P. aeruginosa* mutant construction and complementation

The in-frame deletion mutants in strain PA14 were made using a two-step allelic exchange method^36^. Briefly, sequences (ca. 400-500 bp) flanking *fadE1* (PA14_06600) and *fadE2* (PA14_06640) were PCR-amplified (see Table S2 for a list of the primers used) and fused by overlap-extension PCR. The resulting PCR products were digested *Eco*RI and *Bam*HI and ligated to similarly-digested pEX19Gm. The resulting plasmids were introduced into PA14 by electroporation. Merodiploids were selected on 50 μg mL^-1^ gentamicin. Following sucrose counter-selection on low-salt LB-agar containing 15% w/v sucrose, deletion mutants were confirmed by colony PCR. The Δ*fadE1* Δ*fadE2* double mutant was made by introducing the *fadE2* allele exchange plasmid into the Δ*fadE1* mutant. As the *fadE1* and *fadE2*-encoding regions between PAO1 and PA14 are almost identical, the corresponding mutants in PAO1 were generated in the same way by introducing the above plasmids into PAO1. For complementation, *fadE1* and *fadE2* (inclusive of their cognate ribosome binding sites) were cloned, separately, into pUCP20. The resulting plasmids (or empty vector, as appropriate) were introduced into the indicated mutants by electroporation.

### Enzyme kinetics

Enzyme activity was measured with minor modifications, using the protocol outlined by Lehman et al.^37^. Briefly, 2 μL of appropriately diluted purified FadE1 or FadE2 was added to 198 μL of ferrocenium hexafluorophosphate (0.3 mM) in 50 mM potassium phosphate, 0.1 mM EDTA, 10% v/v glycerol, 0.1 μM FAD (pH 7.0). Reduction of the electron acceptor, ferrocenium hexafluorophosphate (Fc^+^PF_6_^-^), was monitored at 300 nm in an Eppendorf BioSpectrometer® Kinetic. The concentration of the reduced product, ferrocene, was calculated using the molar absorption coefficient ε = 4300 M^-1^cm^-1^. Kinetic parameters were determined using nonlinear regression in GraphPad 8.0, assuming Michaelis–Menten kinetics.

### Crystallization, X-ray diffraction and data collection

FadE1, FadE1^E441A^, FadE2 and FadE2^M130G E296A Q303A^ were crystalized using the sitting drop vapour diffusion method. FadE1 was crystallized in 0.1 M MES 0.1 M calcium chloride, 20% w/v PEG 6000 (pH 6.0). The FadE1^E441A^·C16-CoA complex was obtained from 0.1 M bis-tris propane, 0.2 M sodium nitrate, 20% w/v PEG 3350, 10% v/v ethylene glycol, 5 mM C16-CoA (pH 6.5). FadE2 was crystallized from 0.1 M sodium acetate, 30% (v/v) PEG 300 (pH 4.6). FadE2^M130G E296A Q303A^ was crystallized from 25% (w/v) PEG 1500, 0.1 M PCB (propionate/cacodylate/bis tris propane in 2:1:2 molar ratio) (pH 7.0). Diffraction data were collected at the Diamond Light Source (Harwell, UK) on beamline I04-1 or beamline I04. The data were processed on pipeline xia2 DIALS. Structures were solved by molecular replacement using PHASER^38^ from the CCP4 package^39^. The structure FadE5_Ms_ (PDB code: 6KPT) was used as the starting model. Refinement was performed with COOT^40,41^ and validated by Refmac5^42,43^ in CCP4i. Figures were generated using PyMOL.

### Molecular dynamics simulations

Crystal structures were prepared using protein preparation wizard in Schrödinger by adding hydrogens and missing residues. Waters were removed for all the systems during protein preparation. PROPKA (Schrödinger, LLC, New York, NY, 2023) was used to adjust the charges and protonation states. Structures were optimized and then energy-minimized using the OPLS4 force-field (Schrödinger, LLC, New York, NY, 2023). The crystal structure of FadE1 (Glu441Ala) with bound C16-CoA was mutated back to the wild-type and the corresponding dimers were generated by aligning to the FadE1 structure with bound FAD and C8-CoA. To simulate the effect of specific mutations on binding of the C8-CoA and C16-CoA substrates to FadE1 and FadE2, we used the “mutate residue” module in Schrodinger. MD simulations were carried out using Desmond^44^, with the OPLS4 force-field^45^. The protein-substrate complexes were prepared using water as a solvent (TIP3P water model)^46^ and charges were neutralized with Na^+^ or Cl^-^. Each simulation was carried out by treating the system as if it were in an orthorhombic box with periodic boundary conditions (box size 10 Å). Systems were relaxed before the runs using RESPA integrator, and short-range coulombic interactions were treated using a cut-off value of 9.0 Å. The simulation was performed under the NPT ensemble for 5 ns, implementing the Berendsen thermostat and barostat methods. The simulation was maintained at constant temperature (310 K) and 1 atm pressure using the Nose-Hoover thermostat^47,48^ and Martyna-Tobias-Klein barostat algorithms^49^, respectively. After minimization and relaxation of the system, the production runs were done with sampling every 1000 ps. For each system, five independent replicas of 1 µs were generated, totalling around 5 μs. Raw trajectories and the calculated simulation data have been deposited to Zenodo repository.

Unless otherwise noted, we used Schrödinger python scripts for most of the MD analyses. MD trajectories of each run (1 μs × 5 replicates) were concatenated using trj_merge.py script and analyses were performed on the resulting 5 μs trajectories. The Simulation Interaction Analysis tool was utilized to inspect Root Mean Square Deviation (RMSD), Root Mean Square Fluctuation (RMSF), and protein-substrate interactions based on the Cα atoms and backbone. To inspect the overall changes in each system during a simulation, the Desmond trajectory clustering tool was utilized, based on the backbone RMSD of up to 10 clusters. These clusters were also visualized using PyMOL (v2.5) to inspect the flexible regions and side chain conformations. Distances between the active site residues and entire α-helices were calculated using the trj_asl_distance.py script. These values were plotted using Prism 10.2.

### Molecular docking

System preparation and docking calculations were performed using the Schrödinger Drug Discovery suite for molecular modelling (version 2024.1). Protein−ligand complex was prepared with the Protein Preparation Wizard to fix protonation states of amino acids, add hydrogens, and fix missing side-chain atoms, where we selected the most likely ionization state as proposed by the software, and the structures were energy minimized. All ligands for docking were drawn using Maestro and prepared using LigPrep^50^ to generate the 3D conformation, adjust the protonation state to physiological pH (7.4), and calculate the partial atomic charges with the OPLS4 force field^45^. Docking studies with the prepared ligands were performed using Glide (Glide V7.7)^51,52^ with the flexible modality of induced-fit docking with extra precision (XP), followed by a side-chain minimization step using Prime. Ligands were docked within a grid around 12 Å from the centroid of the co-crystallized structure.

### MM/GBSA binding energy calculations

Molecular mechanics with generalized Born and surface area continuum solvation (MM/GBSA) predicts the binding free energy of protein-ligand complexes. [The ranking of ligands based on these free energies is correlated with experimental binding affinities, especially in a congeneric series.] The docking poses were used as input files for the MM/GBSA calculations using Prime^53^ allowing energy minimization of amino acid side-chains within a 3.5 Å radius around the ligand binding pocket. Calculated free-binding energies are represented by the MM/GBSA and normalized by the number of heavy atoms (HAC), according to the following formula: ligand efficiency = (Binding energy) / (1 + ln(HAC)). Binding energies are expressed in kcal/mol.HAC, where HAC is the Heavy Atom Count.

### Sequence similarity search and phylogenetic tree

Acyl-CoA dehydroegnase sequences from model organisms were retrieved from UniProt, using the term ‘Fad’ as the keyword. Sequences were retrieved from NCBI/GenBank using BLAST searching (with scoring matrix BLOSUM80, for distant similar sequences) against the PDB database creating our dataset for further alignment. Sequence renaming and editing were performed with in-house Perl scripts. The selected sequences underwent global alignment using ClustalOmega^54^. This algorithm often selects single organisms representing a full clade of highly similar sequences, randomly selecting a centroid sequence within the cluster as a representative. The maximum likelihood phylogenetic tree was generated using PhyML 3.0^55^ with posterior probability values (aBayes) as branch statistical support. The substitution model Q.pfam +R + F^56^ was selected for calculations by SMS^57^, based on the highest Bayesian Information Criterion values. All other parameters, except the equilibrium frequencies, were estimated from the dataset. Dendrogram figures were generated using FigTree 1.4.4 (http://tree.bio.ed.ac.uk/software/figtree/“).

### Calculation of d_N_/d_S_ at single codon resolution

The sequences of *fadE1* and *fadE2* were analysed at the single codon level using an in-house dN/dS pipeline based on the likelihood method described by Muse and Gaut^58^. The code for d_N_/d_S_ mapping is available at https://github.com/ph-u/PAO1_fadE_dNdS/. Specifically, we compared the *fadE1* and *fadE2* genes from PAO1 (NCBI accession GCF_000006765.1) against the International *Pseudomonas* Consortium Database (BioProject number PRJNA325248). This analysis was performed on the Cambridge CSD3 Icelake-himem high performance computing cluster. The output was mapped onto the structures of FadE1 and FadE2 in PyMOL (v2.5.0). Domain-specific differences were tested by Wilcox test with Benjamini-Hochberg false discovery rate p-value correction.

### Galleria mellonella infection assay

The *Galleria mellonella* Research Centre (GMRC) colony was reared in-house under standardised conditions at 30°C in constant darkness^59^. Sixty highly motile, unmelanised larvae weighing between 340-380 mg were selected per treatment on the day of injection for experimental use. Colonies of the PA01 progenitor and of the *fadE* mutants were picked and grown overnight in liquid LB broth. Aliquots (50 μL volume) of this starter culture were used to inoculate 5 mL fresh LB broth and the cultures were grown to OD_600_ of ~0.8 and then pelleted (10,000 × *g*, 10 min). The pellets were washed twice with 2 × sterilised phosphate buffered saline (PBS) using the same sedimentation parameters. Cultures were then resuspended to an OD_600_ of 0.30 in sterile PBS (pH 7.4) equating to roughly 2 × 10^8^ colony forming units (CFU) of bacteria. The bacteria were then serially diluted to 2 × 10^3^ CFU mL^-1^ in sterile PBS and 10 μL (equating to 20 CFU per injection) were injected into the right (as viewed dorsally) proleg on the 6^th^ abdominal segment using a 23 gauge 1750 series gastight Hamilton syringe with repeating dispenser. A PBS injected group was used as a control for injection-induced mortality. Each injection mix was also plated on LB agar to confirm the inoculum. Larvae were incubated in 90 mm petri dishes at 37°C in darkness and checked for survival at hourly intervals from 14–24 h post injection. Larvae were scored as dead when they did not respond to a gentle touch to both head and tail segments, showed no motile response when turned over, and exhibited no contractile activity in their dorsal vessel. Sample sizes for the infection assay treatments were calculated using R package “LogrankPower” V1.0.0 (using the methodology described in [37]) powered to detect a 30% difference mortality rate when accounting for Bonferroni corrections for comparison between the four treatment groups. Survival data was analysed using the log-rank (Mantel-Cox) test in GraphPad Prism 10 software package.

### Synthesis of CoA conjugates

The CoA conjugate of compound E was chemically synthesized from 2-cyclobutylacetic acid, and the compound E and its CoA conjugate was synthesized using 3-butynoic acid as starting material. Synthesised compounds were purified by HPLC and were validated by NMR. A full synthesis procedure, HPLC chromatograms and NMR spectrum were provided in Supplementary Information.

## Supplementary information

Description of Additional Supplementary Files

Supplementary Information

Supplementary Dataset 1

Supplementary Dataset 2

Transparent Peer Review file

## Source data

Source Data

## Data Availability

The whole genome sequence of Pa10348 was determined by MicrobesNG and the data were deposited on the NCBI database under accession BioProject PRJNA1008998. The proteomics data were deposited in the PRIDE database under accession number PXD044807, project: https://proteomecentral.proteomexchange.org/cgi/GetDataset?ID=PXD044807. Code used for generating proteomics plots is available at: https://github.com/MWang2025/PAfadE Protein X-ray structures have been deposited on the PDB database with accession numbers 8PNS, 8PNG, 8PU5, 8R1E. The molecular dynamics trajectory data and molecular docking data have been deposited in Zenodo, accession codes: 10.5281/zenodo.8283113; 10.5281/zenodo.12085844, and 10.5281/zenodo.12158295. The dN/dS analysis is available at https://github.com/ph-u/PAO1_fadE_dNdS/. Source data are provided with this paper as a Source Data file. Source data are provided with this paper.
