# Supplementary material for: Pseudomonas aeruginosa acyl-CoA dehydrogenases and structure-guided inversion of their substrate specificity
Source: Nat Commun. 2025 Mar 8;16:2334. doi: 10.1038/s41467-025-57532-z (PMC11890623; doi:10.1038/s41467-025-57532-z)
Supplement: Supplementary file 1 — Description of Additional Supplementary Files [file 41467_2025_57532_MOESM1_ESM.docx]

**Description of Additional Supplementary Files**

**Supplementary Data 1:** Proteomics Data. Source data for Figure 1a-c

**Supplementary Data 2:** Fasta file for proteomics Mascot search
