# Supplementary material for: Pseudomonas aeruginosa acyl-CoA dehydrogenases and structure-guided inversion of their substrate specificity
Source: Nat Commun. 2025 Mar 8;16:2334. doi: 10.1038/s41467-025-57532-z (PMC11890623; doi:10.1038/s41467-025-57532-z)
Supplement: Supplementary file 2 — Supplementary Information [file 41467_2025_57532_MOESM2_ESM.pdf]

**_Pseudomonas aeruginosa_ acyl-CoA dehydrogenases and structure-guided inversion of their substrate specificity.**

Meng Wang[1], Prasanthi Medarametla[1,2], Thales Kronenberger[2,3,4], Thomas Deingruber[5], Paul Brear[1], Wendy Figueroa[1], Pok-Man Ho[1], Thomas Krueger[1], James C. Pearce[6], Antti Poso[2], James G. Wakefield[6], David R. Spring[5], and Martin Welch[1]*

[1]Department of Biochemistry, Tennis Court Road, Cambridge, CB2 1QW, United Kingdom.

[2]School of Pharmacy, University of Eastern Finland, FI-70211, Kuopio, Finland.

[3]Interfaculty Institute of Microbiology and Infection Medicine (IMIT), University of Tübingen, Tübingen, Germany.

[4]Partner-site Tübingen, German Center for Infection Research (DZIF), Tübingen, Germany.

[5] Yusuf Hamied Department of Chemistry, Lensfield Road, Cambridge, CB2 1EW, United Kingdom.

[6] Living System Institute, Department of Biosciences, University of Exeter, UK.

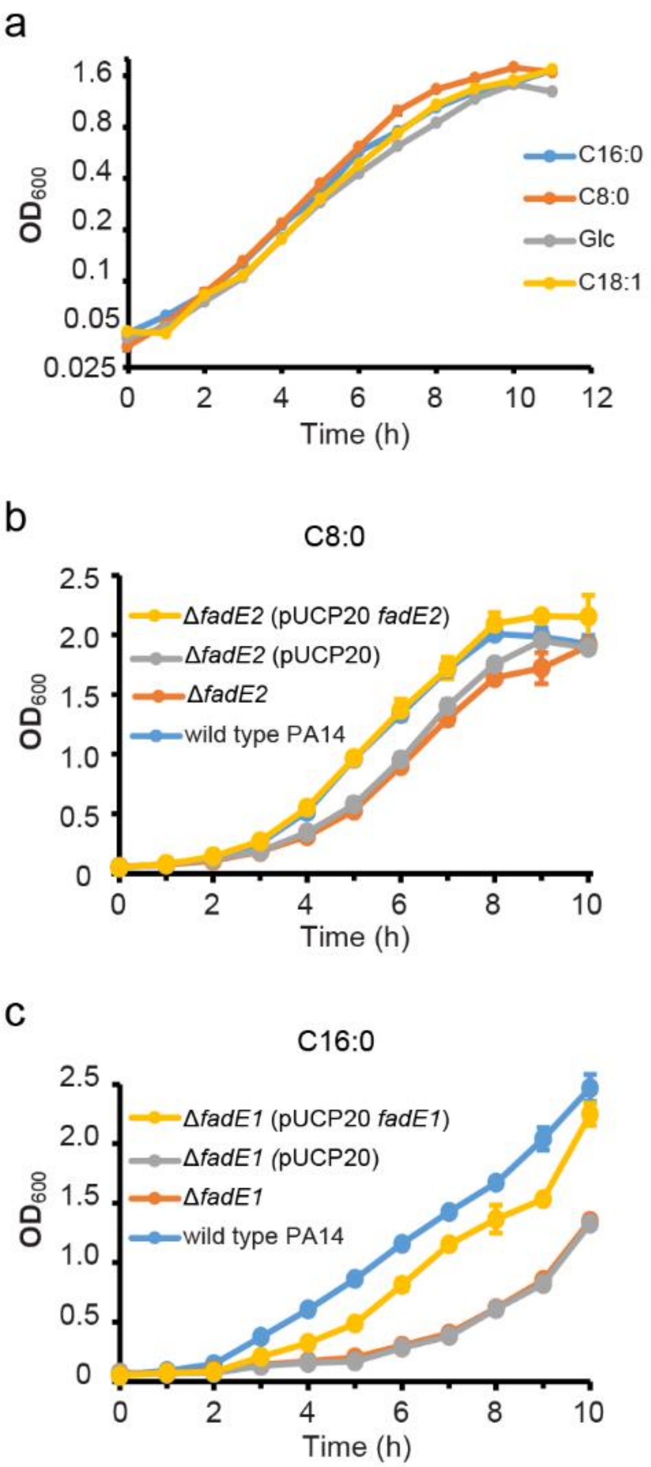


**Figure S1**. Growth of the indicated strains/mutants. **a** Growth of the Manchester epidemic strain (Pa10348) on glucose, C8:0, C16:0 and C18:1$^{\Delta9}$. **b** Growth of wild-type PA14, the Δ*fadE2* mutant, the Δ*fadE2* mutant containing pUCP20 (*fadE2*), and of the Δ*fadE2* mutant containing empty pUCP20 vector, on C8:0 as a sole C-source. **c** Growth of wild-type PA14, the Δ*fadE1* mutant, the Δ*fadE1* mutant containing pUCP20 (*fadE1*), and of the Δ*fadE1* mutant containing empty pUCP20 vector, on C16:0 as a sole C-source. Each data point represents the mean ± SD of three independent biological replicates except the Δ*fadE2* mutant containing pUCP20 (*fadE2*), which represents the mean ± SD of two replicates. Source data are provided as a Source Data file**.**

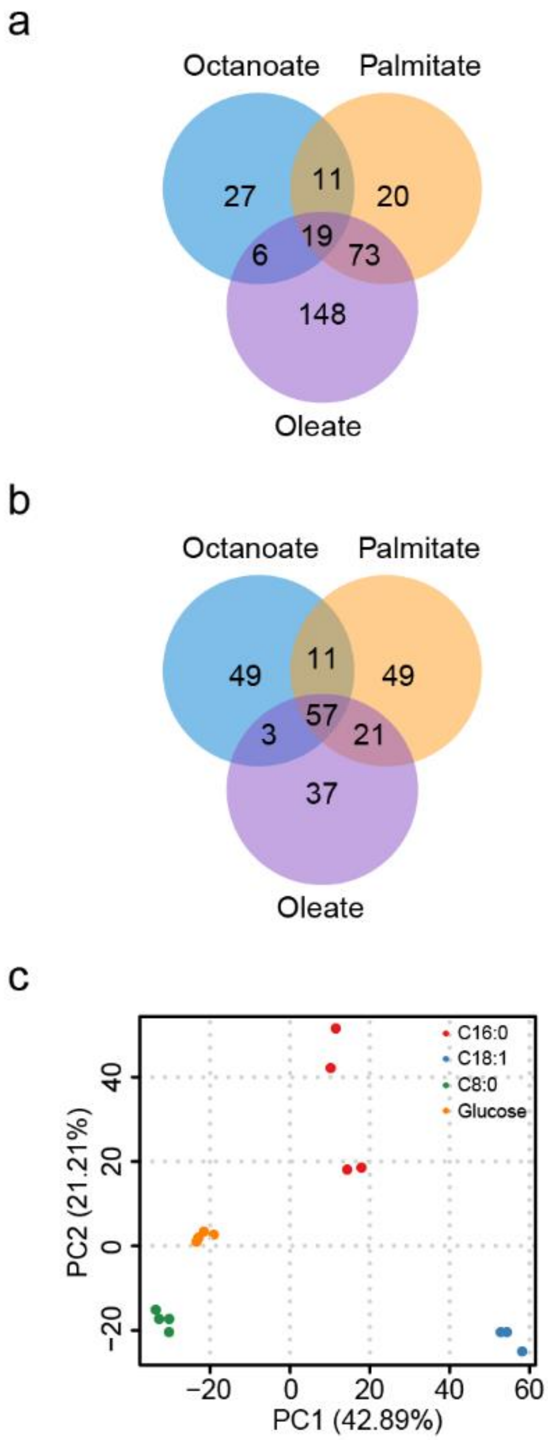


**Figure S2**. Overview of the proteomic data. **a** Venn diagram showing the number of proteins with *increased* abundance ($p < 0.01$) during growth on each fatty acid (*cf*. growth on glucose). **b** Venn diagram showing the number of proteins with *decreased* abundance ($p < 0.01$) during growth on each fatty acid (*cf*. growth on glucose). **c** Principal components analysis (PCA) of *P. aeruginosa* grown on glucose, octanoate (C8:0), palmitate (C16:0) and oleate (C18:1). The PCA plot was generated using the 2640 normalized protein abundances common to all of the samples as an input. Data represent $n$ = 3 replicates for C18:1, and $n$ = 4 replicates for the other carbon sources.

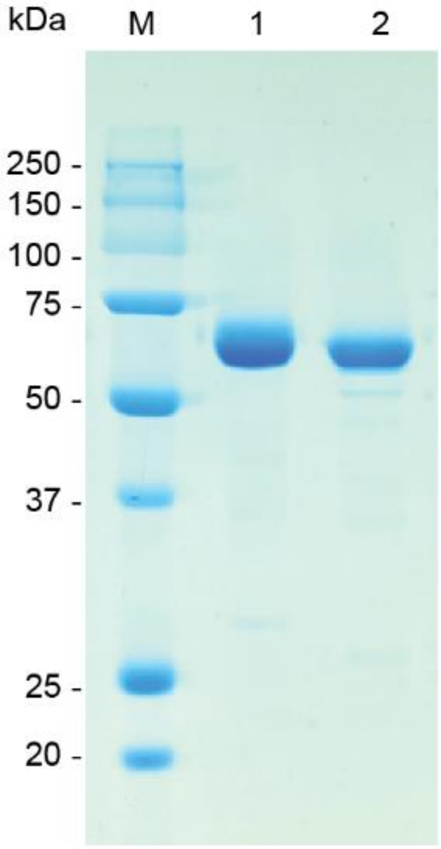


**Figure S3**. The figure shows a Coomassie Brilliant Blue R250 stained 12% SDS-polyacrylamide gel of representative FadE1 and FadE2 protein preparations. FadE1 (lane 1) has a theoretical mass of 65.5 kDa, whereas FadE2 (lane 2) has a theoretical mass of 63.8 kDa. The raw image is provided as a Source Data file.

a

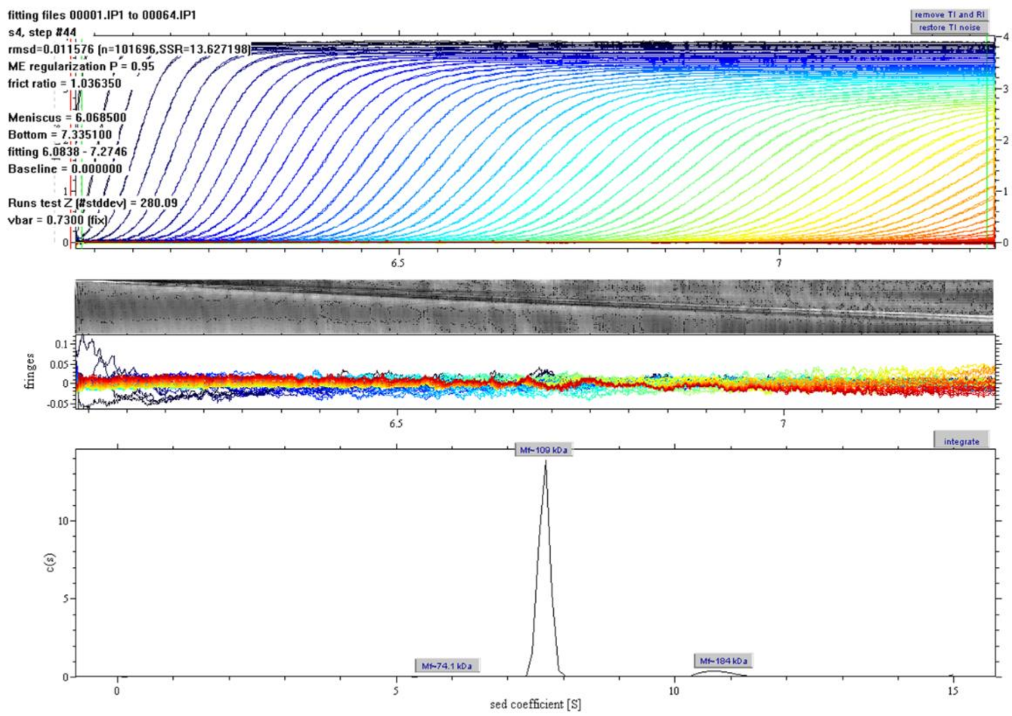


b

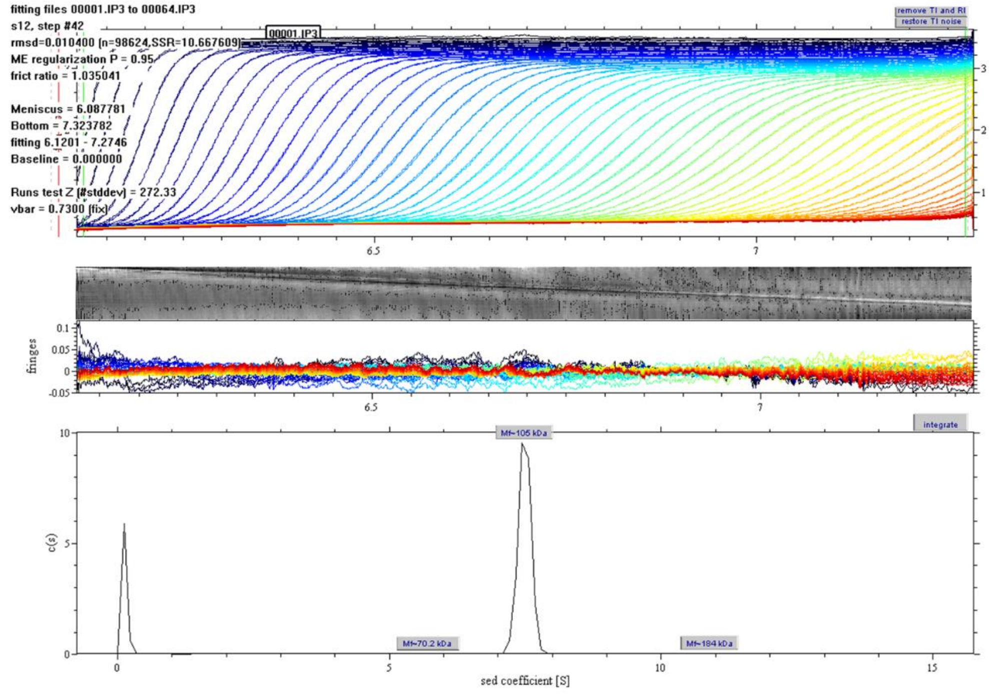


**Figure S4**. Analytical ultracentrifugation (AUC) analysis revealed that **a** FadE1 is a 109 kDa protein in solution, and **b** FadE2 is a 105 kDa protein in solution. Given the theoretical molecular mass of the FadE1 (65.5 kDa) and FadE2 (63.8 kDa) monomers, these values are consistent with FadE1 and FadE2 most likely being dimeric.

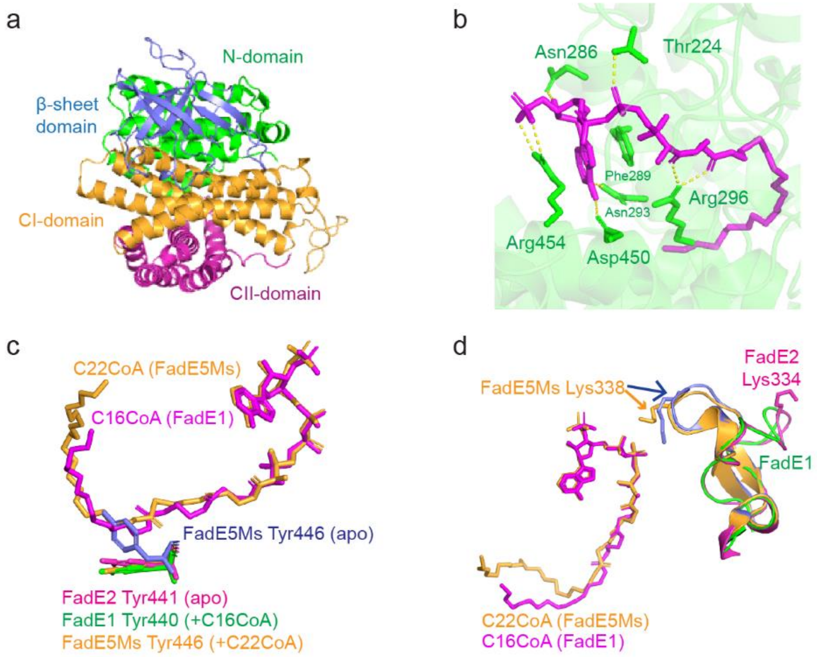


**Figure S5.** Comparison between FadE1 from *P. aeruginosa* and FadE5 from *Mycobacterium smegmatis*. **a** Cartoon showing the domain architecture and nomenclature of FadE1 and FadE2. The order of the domains in the primary sequence is N-domain (α helices shown in green) → β-sheet domain (β-strands shown in blue) → CI-domain (α helices shown in orange) → CII-domain (α helices shown in magenta). **b** Hydrogen bonds and a $\pi$-$\pi$ interaction formed between the FadE1$^{E441A}$ mutant protein and the C16-CoA substrate. The C16-CoA substrate is shown in magenta and the interacting side chains on FadE1$^{E441A}$ are shown in green. **c** Comparison of the configuration of Tyr446 in FadE5$_{Ms}$ (apo form in blue, with bound C22-CoA in orange), Tyr440 in FadE1 with bound C16-CoA (green), and Tyr441 in FadE2 (apo form, pink). Note that the tyrosine residue in PA is likely to be the "open" state in both the presence and absence of the substrate. **d** The loop harboring Lys338 (FadE5$_{Ms}$ numbering) is oriented towards the substrate in FadE5$_{Ms}$ but is oriented away from the substrate in FadE1 and FadE2. RMSD values between *P. aeruginosa* FadE1 (containing bound C16-CoA) and FadE5$_{MS}$ (containing bound C22-CoA or no bound substrate, respectively) are 3.339 Å or 3.536 Å. RMSD values between *P. aeruginosa* apo-FadE2 and FadE5$_{MS}$ (containing bound C22-CoA or no bound substrate, respectively) are 4.756 Å and 4.631 Å.

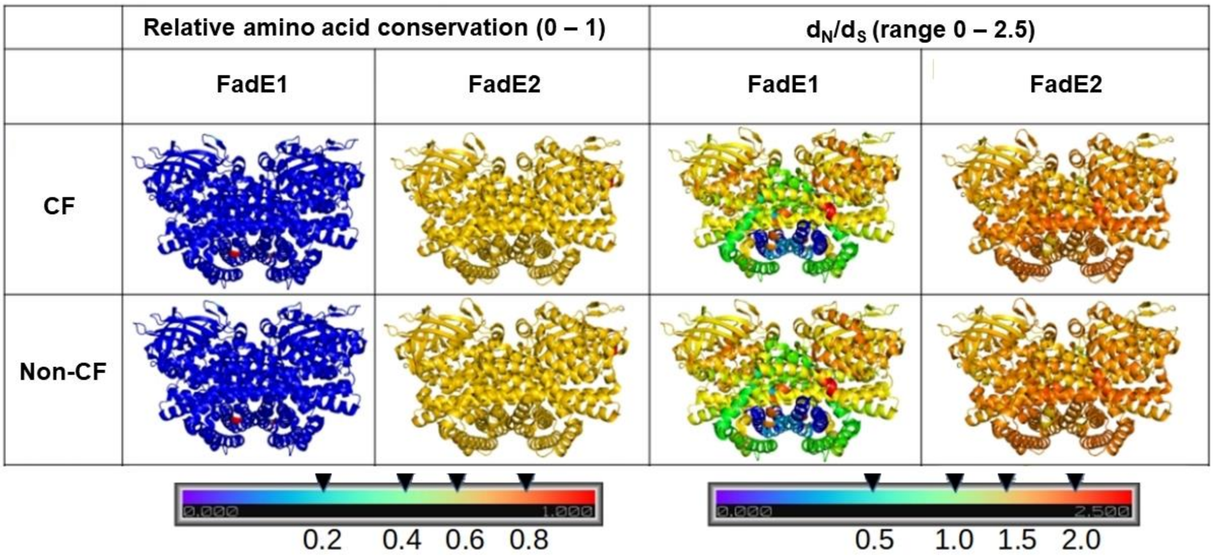


**Figure S6** Protein sequence conservation and $d_N/d_S$ ratio of FadE1 or FadE2 against the CF-derived and non-CF strains. The blue spectrum in the rainbow bar represents for high similarity and the red color trends to low similarity.

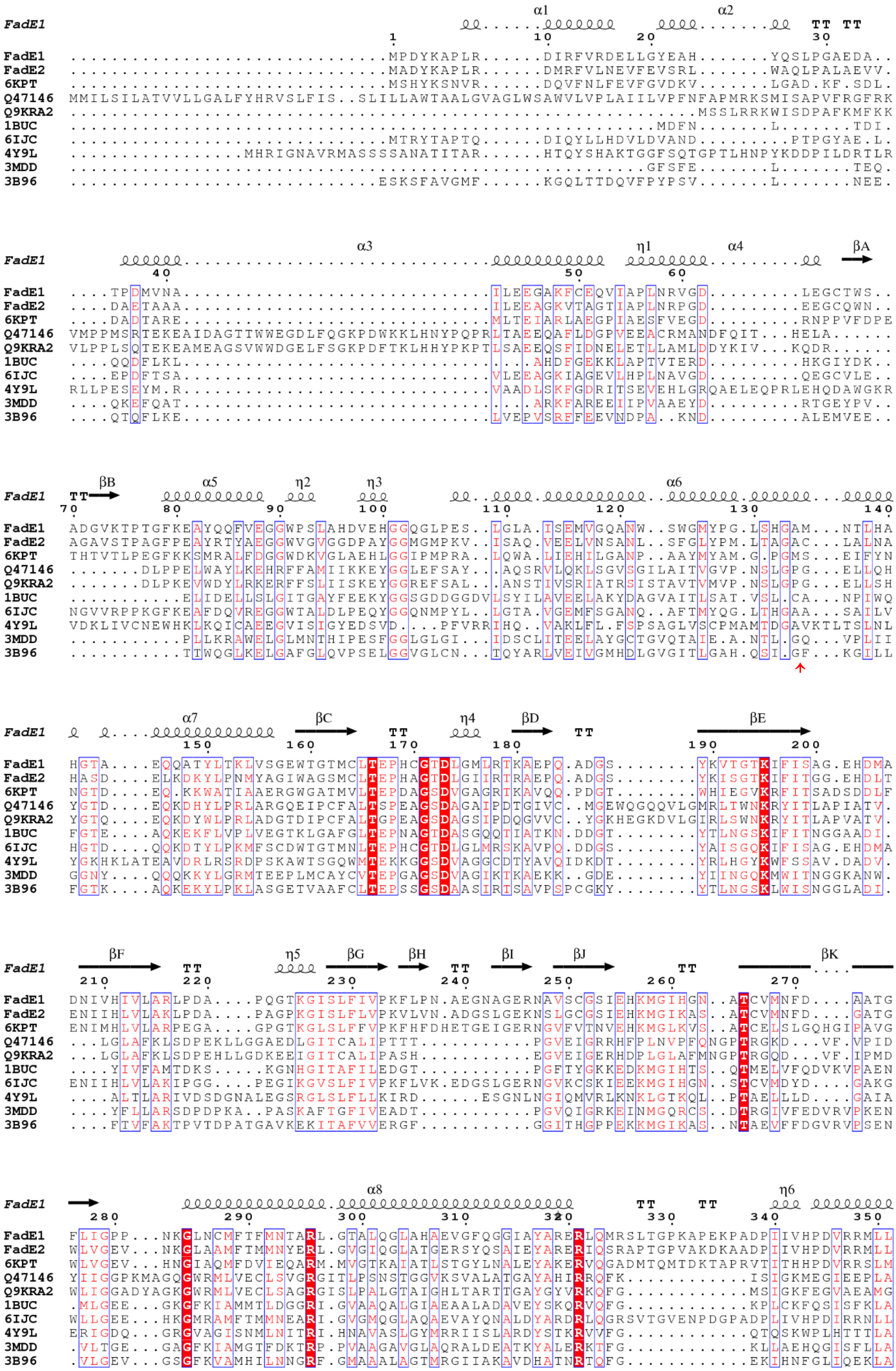
FadE1
FadE2
6KPT
Q47146
Q9KRA2
1BUC
6IJC
4Y9L
3MDD
3B96

```
                α9                                  α10                       η7   η8
FadE1
            360       370       380       390       400       410       420       ★

FadE1   TMKAFAEGNRAMLYFAAKQVDIVQ.RSQDEEQKKAADSMLAFLTPIAKAFMTEVGFESANHGVQIFGGHGFIAEHG..ME
FadE2   TMKALNEGGRAFSSYVAMQLDTAK.YSEDAVTRKRAEELVALLTPVAKAFLTDMGLETTIHGQQIFGGHGFIREWG..QE
6KPT    TQKAYAEGLRAIYLYTATFQDAEVAQAVHGVDGDLAARVNDLLLPIVKGFGSETAYAKLTESLQTLGGSGFLQDYP..IE
Q47146  RIAGNAYVMDAASLITYGIMLG...............EKPAVLSAIVKYHCTHRGQQSIIDAMDITGGKGIMLGQSNFLA
Q9KRA2  RIGGLTYLLEATRTLTTTSLDLK..............EKPGIVTAIAKYHMTEIARTILNDAMDIHSGRAIQDGPMNYLA
1BUC    DMKMQIEAARNLVYKAACK.KQEG.............KPFTVDAAIAKRVASDVAMRVTTEAVQIFGGYGYSEEYP..VA
6IJC    DQKSFIEGARAFLLWGAQMIDRAE.RGKDE....AAHGMVSLLTPVIKGFLTDEGYDMTVQAQQVYGGHGYIEETG..MS
4Y9L    KMEVDTRGSMLLLFEAARLLGL....SEAGKSSDVEAMMLRLITPVLKLYAGKQAVPMVSEGIECFGGQGYMEDTG..LP
3MDD    DMAMKVELARLSYQRAAWE.IDSG.............RRNTYYASIAKAYAADIANQLATDAVQVFGGNGFNTEYP..VE
3B96    RMVMLQYVTESMAYMVSANMDQGA.............TDFQIEAAISKIFGSEAAWKVTDECIQIMGGMGFMKEPG..VE

         α11      η9          α12         η10        α13                 η11
FadE1
        430       440       450       460       470       480       490       500

FadE1   QNVRDSRISMLYEGTTGVQALDLLGRKVLMTQGEALKGFTKIVHKFCQANEAN.EAVKEFVAPLAQLNKEWG.....DLT
FadE2   QLVRDCRITQIYEGTNGIQALDLVGRKVIGSGGAFSRHFTDEIKAFVA..SAD.EALGEFSKPLAAAVENLE.....ELT
6KPT    QYIRDSKIDSLYEGTTAIQAQDFFFRKIIRDKGQALAYVAGEIEQFIKNENGN.GRLKTERELLATALADVQ.....GMA
Q47146  RAYQGAPIAITVEGANILTRSMMIFGQGAIRCH...PYVLEEMEAAKNN.D.....VNAFDKLLFKHIGHVGSNKVRSFW
Q9KRA2  THYLGVPVAITVEGANILTRNLMIFGQGATRCH...PYVLKEMEAAANP.D.QQEGAKAFDELLFKHIAHATKNSFGALF
1BUC    RHMRDAKITQIYEGTNEVQLM.VTGGALLR..................................................
6IJC    QFTRDARIAMIYEGANGVQALDLVGRKLAQDGGKHVMAFFDLVKGFIKEAGTD.GAMAEFTEPLKSASKDLQ.....SAG
4Y9L    TLLRDAQVTPIWEGTTNVLSLDVLR..VFSGKENILLAFGKRVEQLLGNTKTEDEKLKKSKEAVESALKQLQ.....KLL
3MDD    KLMRDAKIYQIYEGTAQIQRI.IIAREHIGRYK...............................................
3B96    RVLRDLRIFRIFEGTNDILRL.FVALQGCMDKGKELSGLGSALKNPFGNAG...LLLGEAGKQLRRRAG.L......GSG
                    *

        α14                                                 α15
FadE1
                          510       520                     530       540

FadE1   MKVGM.............A...AMKDREEVGAASVDYLMYS...............GYACLAYFWADMARLAAE......
FadE2   AWLLD.............R...AKGNPNEIGAASVEYLHVF...............GYTAYAYMWALMARTAL.......
6KPT    ASLTGYLMA.......AQE...DAASIYKVGLGSVRFLMAV...............GDLLSGWLLARQAAVAIE......
Q47146  LGLTRGLTSSTPTGDATKRYYQHLNRLSANLALLSDVSMAVLGGSLKRRERISARLGDILSQLYLA...SAVL.......
Q9KRA2  AALTGSRFIKANMSGPTQDYYKQMTRLSRALAVSADVAMLTLGGELKRKEMISARLGDALSYLYMG...SAVL.......
1BUC    ................................................................................
6IJC    MFFMQ.............N...GMKNPNAALAGSYDFMHLF...............GHVCLGLMWGRMAEASLK......
4Y9L    VKASDSAI................QGETRI..DSVARHIA......................FTIARIYSGAL.......
3MDD    ................................................................................
3B96    LSLSGLVH................PELSRSGELAVRALEQF..ATVVEAKLIKHKKGIVNEQFLLQRLADGAIDLYAMVV

                                α16         η12      α17        α18    η13
FadE1
                550       560       570       580       590

FadE1   .......KLAAGT..GEEAFYKAKLQTARFYFQRILPRTRAH...VAAMLSGANNLMEMAEEDFA...............
FadE2   ..........AKQ..GEDDFYASKLGTARFYFARLLPRIHSL...SASVRAGSESLYLLDAEQF................
6KPT    .......KLDAGATGADKSFYEGKIAAASFFAKNMLPLLTST...RQIIENLDNDVMELDEAAF................
Q47146  ..KR...YDDEGRNEADLPLVHWGVQDALYQA.............................EQAMDDLLQNFPNRVVAGL
Q9KRA2  ..KK...YEDEGRHQADLDYVHYAMQYCLHHA.............................AKSLNQAYRNYPVTGVGAI
1BUC    ................................................................................
6IJC    .......ALAEGR..GDANFHETKLATARFYMTRRLPATKLH...LARIESGADPVMALDADRF................
4Y9L    LIDHASDSSVANQS........DIEVAYRYCCEQPLIDLRWEWFASERVKAD............REIVFDNFTALEKSKI
3MDD    ................................................................................
3B96    VLSRASRSLSEGHPT....AQHEKMLCDTWCI.EA..........AARIREG...MAALQSDPWQQELYRNFKSISKALV

FadE1
                                                    600

FadE1   ..................................................LGY...........................
FadE2   ................................................................................
6KPT    ................................................................................
Q47146  LNVVIFPTGRHYLAPSDKLDHKVAKILQVPNATRSRIGRGQYLTPSEHNPVGLLEEALVDVIAADPIHQRICKELGKNL.
Q9KRA2  LKGLLFPLGNHFAPPSDELAVKLAESLMTPGAHRDRLTALCYIGKGEDDSVGLMEKAFLAMYSVKGLERKLQQGVKEGKV
1BUC    ................................................................................
6IJC    ................................................................................
4Y9L    ................................................................................
3MDD    ................................................................................
3B96    ERGGVV........................................TSNPLGF...........................

FadE1

FadE1   ..................................................................
FadE2   ..................................................................
6KPT    ..................................................................
Q47146  .PFTRLDELAHNALVKGLIDKDEAAILVKAEESRLRSINVDDFDPEELATKPVKLP.EKVRKVEAA
Q9KRA2  ARKGLLVDRLAQAEQAGVLSADEVASILAAEKLRSRAIQVDHFSHDFSQIHTHQTTKPKLNSVA..
1BUC    ..................................................................
6IJC    ..................................................................
4Y9L    ..................................................................
3MDD    ..................................................................
3B96    ..................................................................
```

**Figure S7**. Alignment of FadE1 and FadE2 from PA with acyl-CoA dehydrogenases from other sources (6KPT from *Mycobacterium smegmatis* (referred to in the main body text as FadE5$_{Ms}$), Q47146 from *Escherichia coli*, Q9KRA2 from *Vibrio cholerae*, 1BUC from *Megasphaera elsdenii*, 6IJC from *Roseovarius nubinhibens*, 4Y9L from *Caenorhabditis elegans*, 3MDD from *Sus scrofa* and 3B96 from *Homo sapiens*). Secondary structures have been assigned based the structure of FadE1 in this work. Residues highlighted in red were conserved across all of the acyl-CoA dehydrogenases aligned here. The glutamic acid (Glu441 in FadE1) labelled with a red asterisk is the catalytic base in the enzyme. The residue with an arrow below is one of the residues in FadE5$_{Ms}$ (6KPT) that undergoes a conformational change upon substrate binding but is not conserved in the FadE1 and FadE2 proteins from PA.

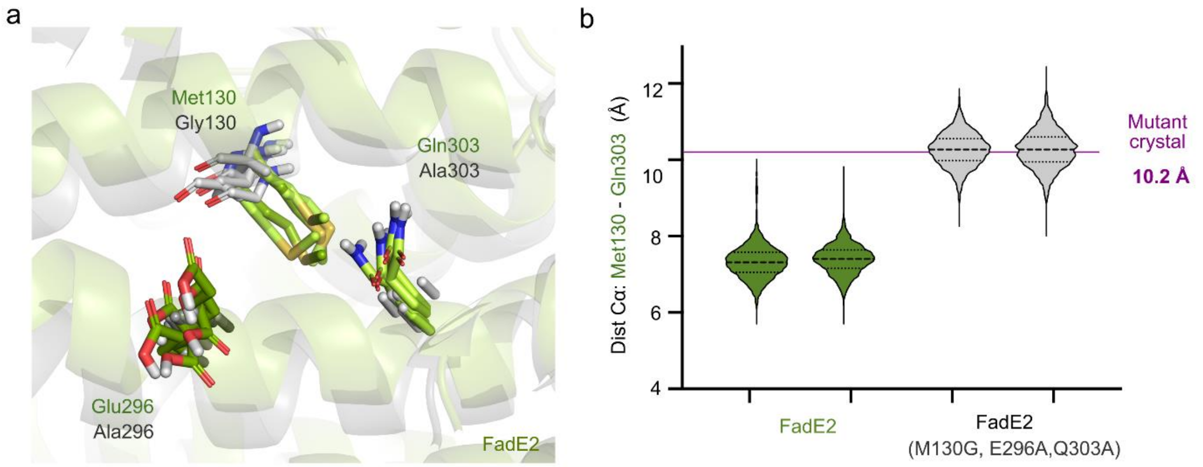


**Figure S8.** Molecular dynamics simulation of the substrate binding tunnel of FadE2. **a** Cartoon representation showing the dynamics of the indicated residues in wild-type FadE2 (green) and in the FadE2$^{M130G\ E296A\ Q303A}$ triple mutant (grey). **b** Distribution of distances between the Cα atoms of residues 130 and 303 in wild-type FadE2 (green) and in the FadE2$^{M130G\ E296A\ Q303A}$ triple mutant (grey) based on MD analyses. Note how the substrate tunnel is more open (by ca. 3 Å) in the triple mutant, and that the FadE2 mutant crystal structure opening distance (purple line) agrees closely with the simulation median values (10.2 Å).

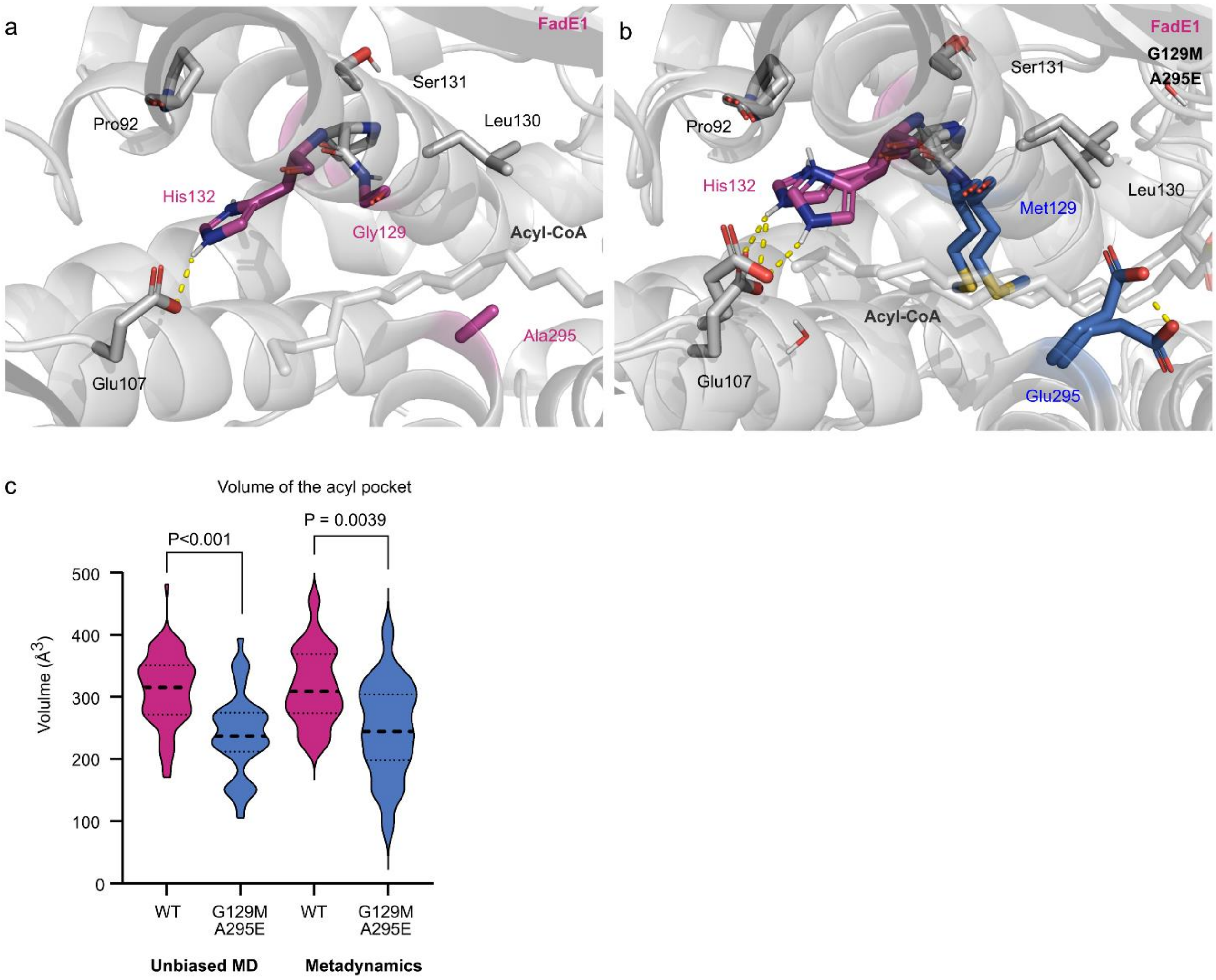


**Figure S9**. Molecular dynamics (MD) simulations of the substrate binding cavity in wild-type FadE1 and in the FadE1$^{G129M\ A295E}$ double mutant protein. **a.** Wild-type FadE1 with bound substrate (C16-CoA); the "wild-type" FadE1 structure here is based on the FadE1$^{E441A}$ crystal structure in which residue Ala441 has been mutated (*in silico*) back to glutamate (comparing the backbone of the "wild-type" structure obtained this way with the experimentally-determined FadE1$^{E441A}$ mutant structure yielded an RSMD of 0.144 Å). Key amino acid residues are labelled and shown as sticks. The location of the substrate (C16-CoA) is shown in grey. **b** FadE1$^{G129M\ A295E}$ double mutant with bound C16-CoA. **c** MD simulations with the apo-structure for wild-type (WT) FadE1 and for the FadE1$^{G129M\ A295E}$ double mutant (G129M A295E) protein were used to evaluate differences in the acyl binding pocket volume. We employed both classical unbiased simulations (10 × 200 ns per system) and metadynamics (5 × 400 ns per system). Kolmogorov-Smirnov tests were performed to compare the wild-type (purple) and double mutant (blue) simulations. The cumulative distribution and exact p-values are depicted. Median volume for wild-type FadE1 and for the FadE1$^{G129M\ A295E}$ double mutant, respectively, were: 315.4 Å$^3$ and 237.3 Å$^3$ (classical simulations) and 309.1 Å$^3$ and 244.2 Å$^3$ (metadynamics trajectories), suggesting similar sampling amplitude in both independent approaches.

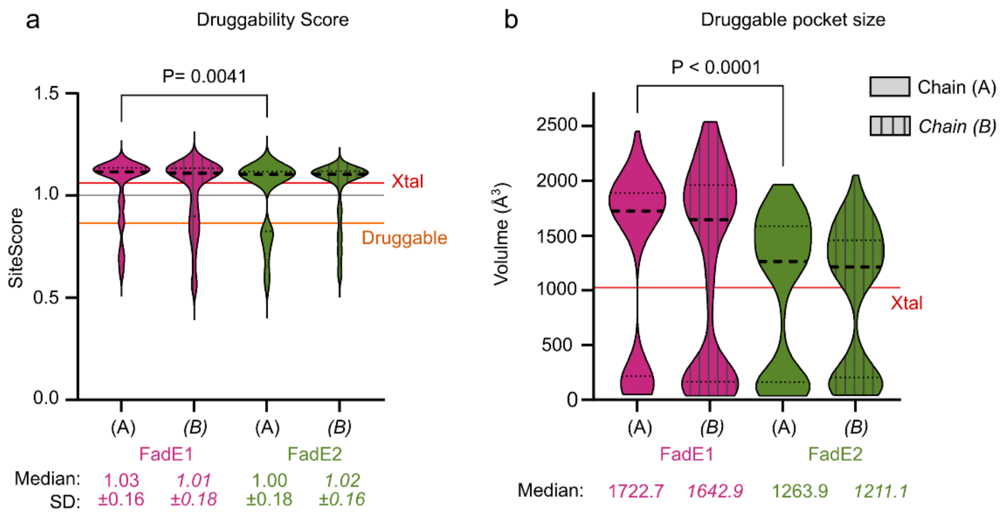


**Figure S10**. Dimensions and druggability of the FadE1 and FadE2 substrate binding pockets. **a** The fatty acid binding pocket in FadE1 and FadE2 was identified as having the highest druggability score in MD trajectories. **b** The overall fatty acid binding pocket volume of FadE1 is larger than that in FadE2. Mann-Whitney tests were performed to compare FadE1 (purple) against FadE2 (green). The cumulative distribution (chain-by-chain) and exact p-values are depicted when available.

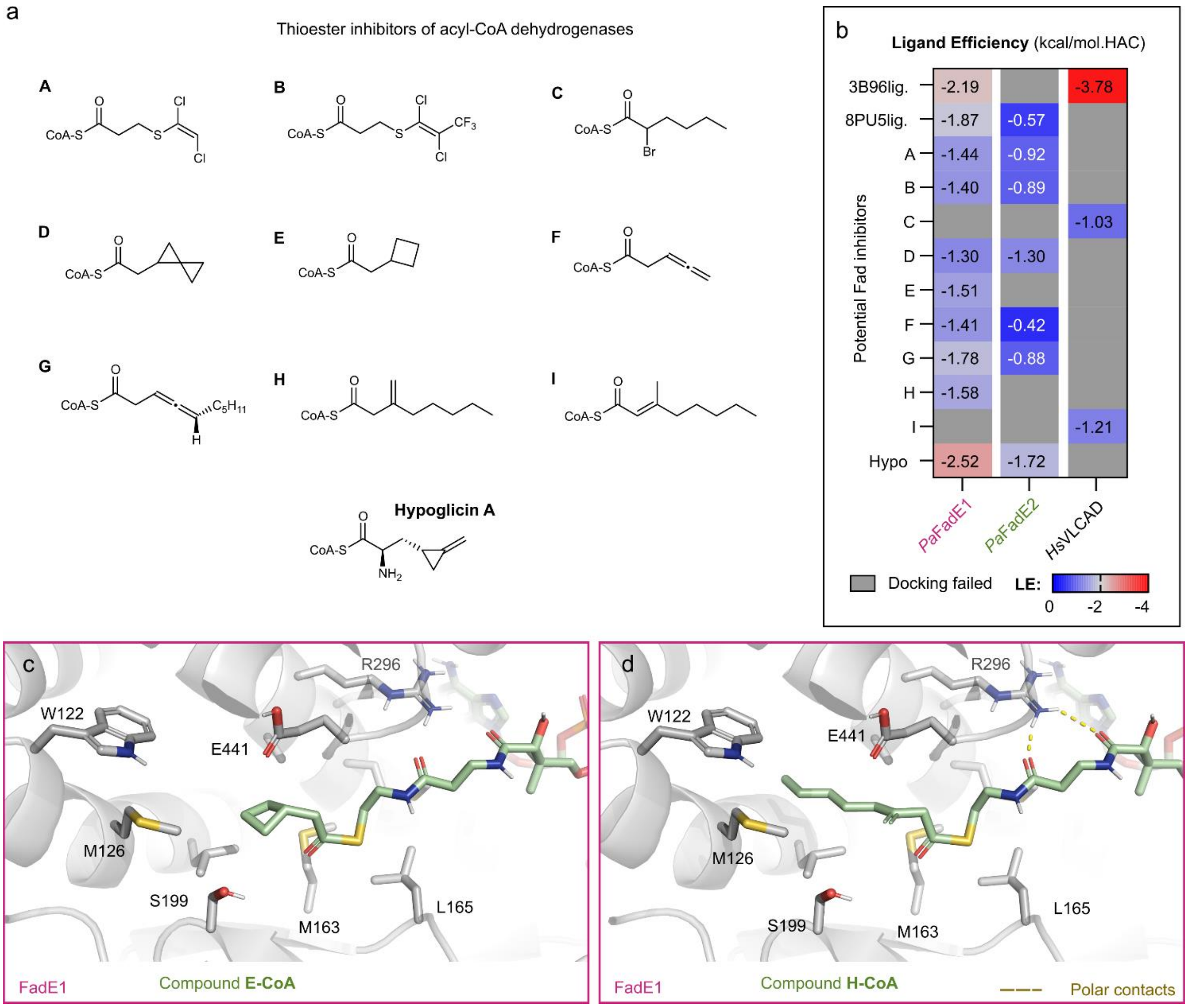


**Figure S11**. Selective inhibition of FadE1 by small molecules. **a** Structures of the acyl-CoA dehydrogenase thioester inhibitors we tested[1]. The compounds were docked in the fatty acid binding pocket of FadE1 (PDB 8PU5), FadE2 (PDB 8PNG) and the human very long chain dehydrogenase (HsVLCAD, PDB 3B96). The docking poses underwent energy minimization and their potential binding energy was calculated using MM/GBSA. **b** Heatmap representation of ligand efficiency (binding energy/number of heavy atoms) for the possible poses associated with each inhibitor. Grey shading indicates ligands which yielded no docking pose in the respective protein. **c-d** Examples of the potential binding mode for compound E (**c**) and compound H (**d**) in the fatty acid binding pocket of FadE1. Relevant interactions with the acyl portion of the inhibitors are shown as dashed yellow lines.

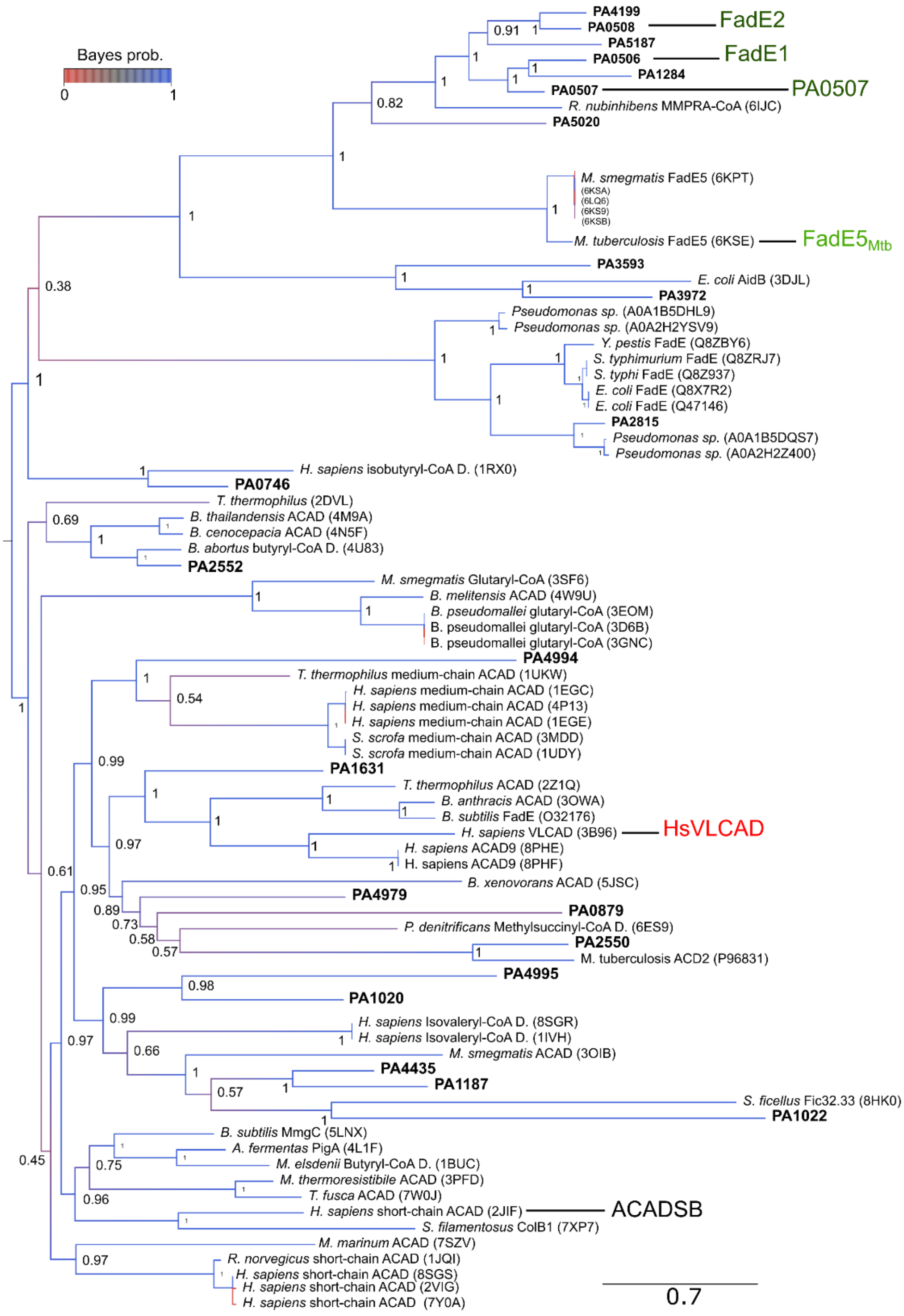


**Figure S12**. Phylogenetic tree showing evolutionary relationships within the FadE acyl-CoA dehydrogenase (ACAD) protein family. Phylogenetic inferences were generated using the maximum likelihood method (see Methods). Branch support values (Bayes posterior probabilities) are color-coded as indicated, and are displayed as numbers for the most relevant clade separations. Proteins are labelled according to the species from which they are associated, followed by the protein name/function and by their 4-letter PDB code or their UniProt accession code (in parentheses). Proteins mentioned in the current manuscript are highlighted, along with human HsVLCAD and the human short/branched chain acyl-CoA dehydrogenase, ACADSB. All 22 acyl-CoA dehydrogenases encoded by *P. aeruginosa* (PAO1) are indicated in bold.

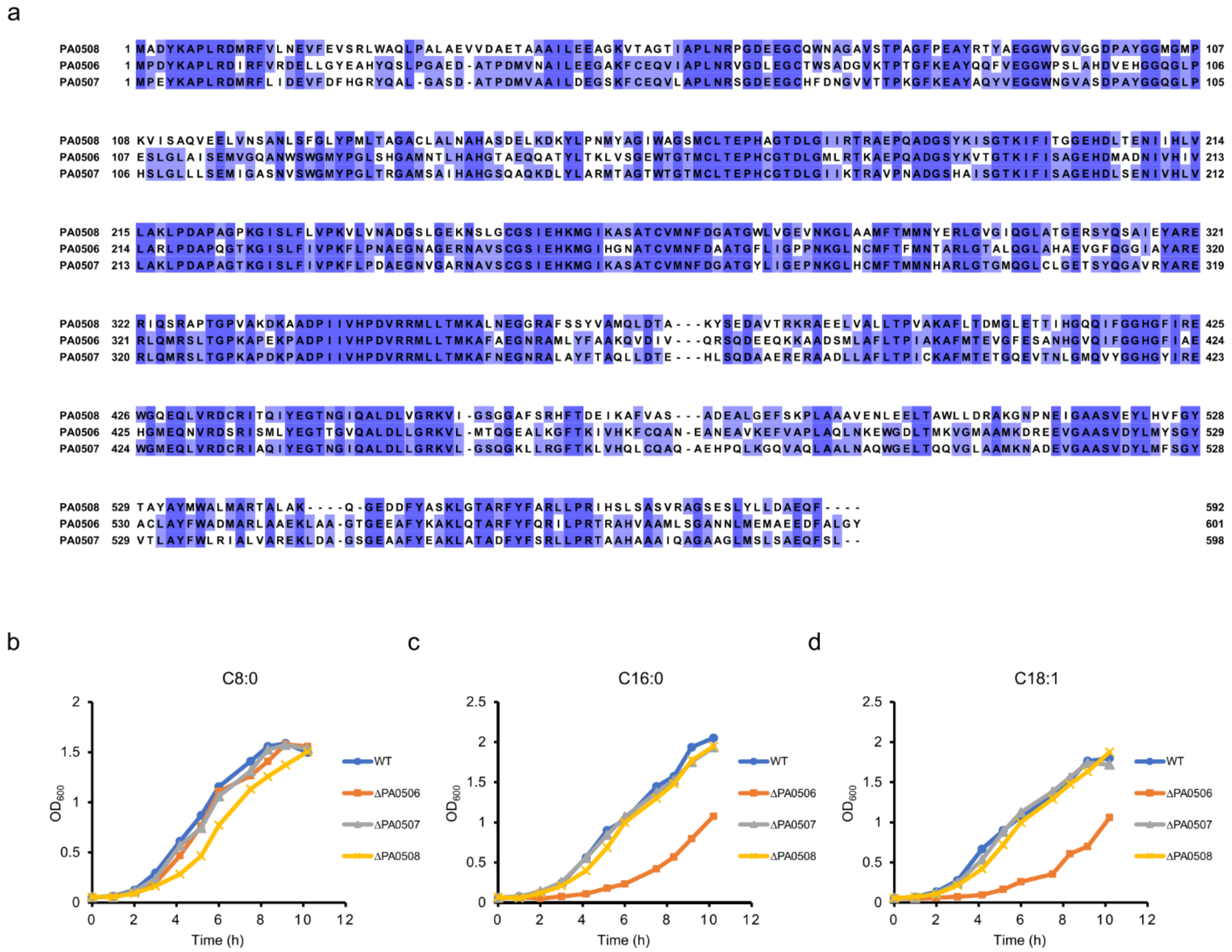


**Figure S13**. Loss of PA0507 does not affect growth on C8, C16, or C18:1 fatty acids. The ORF (PA0507) located between *fadE1* and *fadE2* encodes an acyl-CoA dehydrogenase paralogue with >50% identity to FadE1 and FadE2. **a** Lineup of amino acid sequences of FadE1 (PA0506), FadE2 (PA0508) and PA0507. **b-d** Growth phenotypes (n = 1) of a deletion mutant of PA0507 (ΔPA0507) compared with the deletion mutants of *fadE1* (Δ0506) and *fadE2* (Δ0508) on the indicated carbon sources. Based on these data, the Δ0507 mutant appears to have no obvious growth phenotype. Source data are provided as a Source Data file.

**Supplementary table 1**. Data collection and refinement statistics

| Parameters | FadE1 (PA0506) | Apo FadE2 (PA0508) | FadE1 E441A C16-CoA | FadE2 M130G E296A Q303A |
|---|---|---|---|---|
| **PDB code** | 8PNS | 8PNG | 8PU5 | 8R1E |
| Synchrotron/X-ray source | Diamond Light Source | | | |
| beamline | I04-1 | I04 | I04-1 | I04 |
| **Data collection** | | | | |
| Wavelength (Å) | 0.9179 | 0.9795 | 0.9179 | 0.9537 |
| Resolution range (Å) | 135.03-2.08 (2.12-2.08) | 64.40-1.94 (1.97-1.94) | 128.78-1.44 (1.46-1.44) | 71.99-1.62 (1.65-1.62) |
| Space group | P61 | $P2_12_12_1$ | $P3_112$ | $P2_12_12_1$ |
| *a*, *b*, *c* (Å) | 155.92, 155.92, 99.51 | 61.58, 64.40, 286.97 | 92.96, 92.96, 128.78 | 61.89, 64.76, 287.95 |
| α, β, γ (°) | 90, 90, 120 | 90, 90, 90 | 90, 90, 120 | 90, 90, 90 |
| Asymmetric unit content | 2 monomers | 2 monomers | 1 monomer | 2 monomers |
| Total reflections | 1315596 | 2300337 | 4425463 | 1810830 |
| Unique reflections | 82487 | 86018 | 114956 | 147729 |
| Multiplicity | 15.9 (11.1) | 26.7 (22.5) | 38.5 (29.3) | 12.3 (6.0) |
| Completeness (%) | 100.00 (99.2) | 100 (98.5) | 100 (100) | 99.76 (94.93) |
| Mean $I/\delta(I)$ | 8.2 (0.4) | 6.1 (0.4) | 11.5 (0.4) | 9.66 (0.32) |
| Wilson B factor | 26.610 | 22.080 | 15.100 | 17.300 |
| $R_{merge}$ | 0.238 (2.900) | 0.476 (8.337) | 0.194 (4.451) | 0.1443 (2.6324) |
| $R_{meas}$ | 0.246 (3.041) | 0.485 (8.529) | 0.197 (4.529) | 0.150 (2.884) |
| $R_{p.i.m.}$ | 0.061 (0.905) | 0.093 (1.788) | 0.031 (0.834) | 0.042 (1.143) |
| $CC_{1/2}$ | 0.996 (0.370) | 0.995 (0.305) | 0.999 (0.333) | 0.998 (0.310) |
| **Refinement** | | | | |
| Resolution range (Å) | 135.03-2.08 | 62.91-1.94 | 80.63-1.44 | 71.99-1.62 |
| No. of used reflections | 78359 | 81637 | 109044 | 144075 |
| R-work | 0.216 | 0.209 | 0.194 | 0.205 |
| R-free | 0.254 | 0.261 | 0.218 | 0.234 |
| No. non-hydrogen atoms | 9545 | 9701 | 5145 | 9916 |
| No. atoms | | | | |
| Protein | 9165 | 8965 | 4605 | 8930 |
| Ligand/ion | 234 | 194 | 119 | 134 |
| Water | 146 | 542 | 421 | 852 |
| B-factor ($Å^2$) | | | | |
| Protein | 42.9 | 35.6 | 23.1 | 30.2 |
| Ligand/ion | 58.9 | 44.8 | 35.8 | 31.1 |
| Water | 33.5 | 39.3 | 36.9 | 35.6 |
| Ramachandran plot | | | | |
| Favored (%) | 92 | 92 | 92.8 | 93.4 |
| Allowed (%) | 8 | 7.8 | 7.2 | 6.6 |
| Disallowed (%) | 0 | 0.2 | 0 | 0 |
| RMSD | | | | |
| Bond lengths (Å) | 0.010 | 0.007 | 0.010 | 0.008 |
| Bond angles (°) | 1.699 | 1.422 | 1.629 | 1.479 |

**Supplementary table 2**. Primers and vectors used in this study.

| Primer | Sequence (5'-3') |
|---|---|
| **In-frame deletion (pEX19Gm vector)** | |
| *fadE1* Up F | CCGGAATTCAGGCCGGCAGCGAGATCG |
| *fadE1* UP R | GTAACCGAGGGCCTTGTAATCAGGCATAG |
| *fadE1* Dn F | TACAAGGCCCTCGGTTACTGATGG |
| *fadE1* Dn R | CGCGGATCCATCGCTGGCGACACCGTTCC |
| *fadE2* UP F | CGGAATTCGTTCTCCGGCTACGTGACC |
| *fadE2* UP R | GCCTCAGAACATGTGGGGGAATCCTCG |
| *fadE2* Dn F | TCCCCCACATGTTCTGAGGCCTGGCGC |
| *fadE2* Dn R | CGGGATCCGAAGCGACTGCCGATGAAC |
| **Site-directed mutagenesis and protein expression (pET19m vector)** | |
| *fadE1* F | GGAATTCCATATGCCTGATTACAAGGCCCCC |
| *fadE1* R | CGCGGATCCTCAGTAACCGAGGGCGAAATC |
| *fadE2* F | GGAATTCCATATGGCTGATTACAAAGCTCC |
| *fadE2* R | CGGGATCCTCAGAACTGCTCGGCGTCC |
| *fadE2* E442A Up | CACGCAGATCTACGcAGGCACCAATG |
| *fadE2* E442A Dn | GAATGCCATTGGTGCCTgCGTAGATCTGCGTGATG |
| *fadE2* M130G Up | GGCGCCGGCGGTCAGCccCGGATACAGGCC |
| *fadE2* M130G Dn | GGCCTGTATCCGggGCTGACCGCCGGCGCC |
| *fadE2* E296A Q303A Up | GCCAGGCCCGcGATGCCGACGCCAAGGCGCgCGTAGTTCATC |
| *fadE2* E296A Q303A Dn | GATGAACTACGcGCGCCTTGGCGTCGGCATCgcGGGCCTGGC |
| *fadE1* E441A Dn | ATCTCGATGCTGTACgcaGGCACCACCGGCGTTCAGG |
| *fadE1* E441A Up | CCTGAACGCCGGTGGTGCCtgcGTACAGCATCGAGAT |
| *fadE1* G129M Up: | GGCGCCGTGGGACAGcatCGGGTACATGCC |
| *fadE1* G129M Dn: | GGCATGTACCCGatgCTGTCCCACGGCGCC |
| *fadE1* A295E Up | GCGGTACCCAGGCGttCGGTGTTCATGAAGGTG |
| *fadE1* A295E Dn | CACCTTCATGAACACCGaaCGCCTGGGTACCGC |
| *fadE1* His132A Up | CAGGGTGTTCATGGCGCCGgcGGACAGGCCCGGGTACAT |
| *fadE1* His132A Dn | ATGTACCCGGGCCTGTCCgcCGGCGCCATGAACACCCTG |
| **Complementation (pUCP20 vector)** | |
| *fadE1* RBS F | CGGGATCCTTCGTAGAGGTTGACTGCTATGCC |
| *fadE1* RBS R | CCCAAGCTTCCATCAGTAACCGAGGGCG |
| *fadE2* RBS F | CGGAATTCACGATCGAGGATTCCCCCAC |
| *fadE2* RBS R | CGGGATCCTCAGAACTGCTCGGCGTCC |
| **Transcriptional reporter (pLP170 vector)** | |
| *fadE1* pLP F | GGAATTCGCCCGGGTCATCGAGGTAG |
| *fadE1* pLP R | CGGGATCCTAGCAGTCAACCTCTACGAAGGG |
| *fadE2* pLP F | GGAATTCCTGGCCTATTTCTGGTTGCGC |
| *fadE2* pLP R | CGGGATCCGTGGGGGAATCCTCGATCGTAC |

**Supplementary table S3** Proteomics sample labelling data

| Sample name | Growth condition | Replicate | Tag |
|---|---|---|---|
| ctrl_glu 1 | Glucose | 1 | TMT126 |
| ctrl_glu 2 | Glucose | 2 | TMT127N |
| ctrl_glu 3 | Glucose | 3 | TMT127C |
| ctrl_glu 4 | Glucose | 4 | TMT128N |
| c8 1 | Octanoate (C8:0) | 1 | TMT128C |
| c8 2 | Octanoate (C8:0) | 2 | TMT129N |
| c8 3 | Octanoate (C8:0) | 3 | TMT129C |
| c8 4 | Octanoate (C8:0) | 4 | TMT130N |
| c16 1 | Palmitate (C16:0) | 1 | TMT130C |
| c16 2 | Palmitate (C16:0) | 2 | TMT131N |
| c16 3 | Palmitate (C16:0) | 3 | TMT131C |
| c16 4 | Palmitate (C16:0) | 4 | TMT132N |
| c18 1 | Olate (C18:1) | 1 | TMT132C |
| c18 2 | Olate (C18:1) | 2 | TMT133N |
| c18 3 | Olate (C18:1) | 3 | TMT133C |
| c18 4 | Olate (C18:1) | 4 | TMT134N |

# Supplementary Methods

## Synthesis of FadE inhibitors

### Solvents and chemicals.

Unless otherwise stated, reactions were carried out in oven-dried glassware under nitrogen atmosphere. All other reagents were used as provided by commercial sources (Sigma Aldrich, Fluorochem, Fischer Scientific). Coenzyme A, trilithium salt, dihydrate (CAS: 18439-24-2, CoA-SH) was obtained from MP Biomedicals Germany GmbH. TFA stands for trifluoroacetic acid.

### Infrared spectroscopy.

Infrared spectra were recorded on an Agilent Cary 630 FTIR with single bounce diamond ATR accessory using neat compounds. Absorption maxima ($\tilde{\nu}_{max}$) are reported in wavenumbers ($cm^{-1}$) rounded to a whole number. Peaks above 1500 $cm^{-1}$ are reported and the following abbreviations are used to describe their appearance: w, weak; m, medium; s, strong; br, broad.

### NMR spectroscopy.

Magnetic resonance spectra were recorded at 298 K using an internal deuterium lock on Bruker Avance III HD (500 MHz; Smart probe) and 700 MHz TXO (700 MHz, Cryoprobe) spectrometers. Proton chemical shifts (δH) are quoted in ppm to the nearest 0.01 ppm and are referenced to the residual non-deuterated solvent peak ($CDCl_3$: 7.26, $D_2O$: 4.79). Carbon chemical shifts ($\delta_C$) are quoted in ppm to the nearest 0.1 ppm and are referenced to the deuterated solvent for $CDCl_3$ (77.2 ppm) or unreferenced for samples in $D_2O$. Phosphorus chemical shifts ($\delta_P$) are quoted in ppm to the nearest 0.1 ppm, are not referenced and were measured with proton decoupling. Coupling constants (J) are reported in Hertz to the nearest 0.1 Hz. Data are reported as follows: chemical shift, integration, multiplicity [br, broad; app, apparent; s, singlet; d, doublet; t, triplet; q, quartet; quint, quintet; sext, sextet; sept, septet; m, multiplet; or as a combination of these (e.g. app s, dd, dt, etc.)], coupling constant(s) and assignment. Proton and carbon assignments are supported by DEPT135, 1H-$^{1}$H COSY, $^{1}$H-$^{13}$C HSQC and $^{1}$H-$^{13}$C HMBC spectra. The numbering of the compounds does not follow IUPAC convention.

### Column chromatography and TLC.

Flash chromatography was done using Millipore Silica gel 60 (0.040–0.063 mm) and distilled solvents. Thin layer chromatography was done using Supelco glass plates covered with TLC Silica gel 60 F254 and distilled solvents as eluents. The TLC plates were visualised by UV unless otherwise specified. If specified, the KMnO4 stain (1.05 g of $KMnO_4$, 7 g of $K_2CO_3$, 90 mg of NaOH in 100 mL of water) was used. The retention factor Rf was quoted to two decimal places.

### HPLC

Analytical HPLC was done on Agilent 1200 Series, fitted with a quaternary pump, using Agilent Eclipse Plus C18 (4.6 mm x 150 mm, particle size 3.5 µm, porosity 95 Å) column and operated by ChemStation B.04.03 software. The LC systems used a linear gradient of solvent B (acetonitrile with 0.05% TFA) in solvent A (water with 0.05% TFA) run over 15min, flow rate 1 mL/min, and UV absorption was measured using a diode-array detector at the wavelength of 254 nm. Preparative HPLC was performed on Agilent 1260 Infinity Series, fitted with a binary pump cluster, Agilent 10 Prep-C18 (21.2 mm x 250 mm, particle size 10 µm, porosity 100 Å) column and operated by ChemStation C.01.07 software. The LC system used a linear gradient of solvent B (acetonitrile with 0.05% TFA) in solvent A (water with 0.1% TFA), flow rate 20 mL/min, and fractions were collected based on absorption at 254 nm.

## HRMS

High-resolution mass spectrometry was measured on Waters Vion IMS QTof using ESI techniques. Mass values are reported within the error limits of ±5 ppm mass units.

## Synthetic scheme

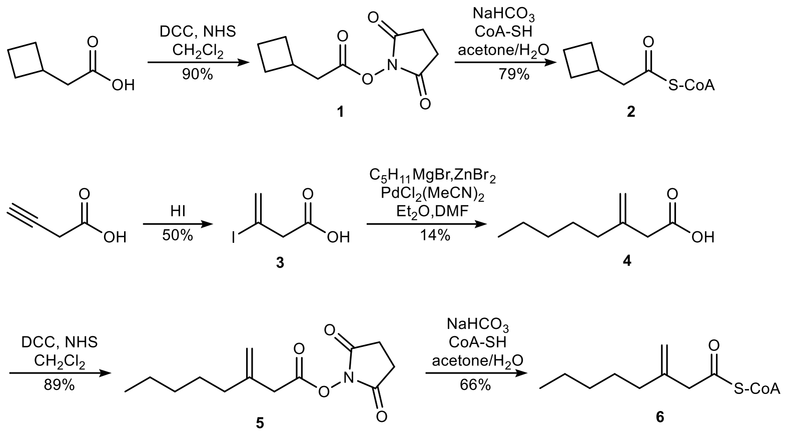


## Experimental procedures

## 2,5-dioxopyrrolidin-1-yl 2-cyclobutylacetate (1)

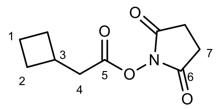

2-Cyclobutylacetic acid (228 mg, 2.00 mmol) and *N*-hydroxysuccinimide (230 mg, 2.00 mmol) were mixed in dichloromethane (5 mL). A solution of *N,N'*-dicyclohexylcarbodiimide (495 mg, 2.40 mmol) in dichloromethane (2.5 mL) was added dropwise and the mixture was stirred overnight at room temperature. The reaction mixture was filtered through Celite and the filtrate concentrated *in vacuo*. The crude material was purified by normal-phase chromatography (gradient of ethyl acetate 0–40% in petroleum ether) to yield 1 (390 mg, 1.85 mmol, 90%) as a white solid.

**TLC:** $R_f$ 0.31 (3:7 ethyl acetate/petroleum ether)

**1H NMR (500 MHz, $CDCl_3$):** δ 1.75–1.98 (4H, m, H-1 and H-2), 2.15–2.24 (2H, m, H-2), 2.69 (2H, d, J = 7.7 Hz, H-4), 2.77 (1H, sept, J = 7.7 Hz, H-3), 2.82 (4H, br s, H-7)

**13C NMR (126 MHz, $CDCl_3$):** δ 18.5 (C-1), 25.7 (C-7), 28.2 (C-2), 31.9 (C-3), 37.7 (C-4), 167.6 (C-5), 169.3 (C-6)

FT-IR (neat): 2984 (w), 2963 (w), 2939 (w), 2861 (w), 1813 (m), 1781 (m), 1725 (s)

## 2-cyclobutylacetyl Coenzyme A (2)

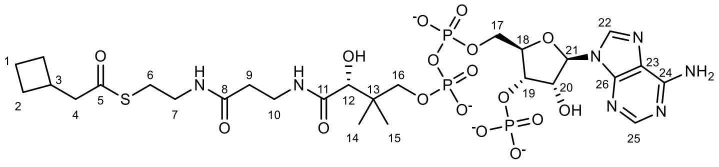

Synthesised according to modified procedure by Gao *et al.*[2] Compound **1** (30 mg, 0.14 mmol) was dissolved in acetone (0.4 mL), **CoA-SH** (26 mg, 0.032 mmol) was dissolved in water (0.4 mL) and $NaHCO_3$ (40 mg, 0.48 mmol) was dissolved in water (1.2 mL). The **CoA-SH** and $NaHCO_3$ solutions were combined at 0 °C and stirred for 10 min. Subsequently, the compound **1** solution was added dropwise and more acetone added dropwise until a clear homogeneous solution formed. The mixture was stirred at 0 °C for 5.5 h, after which the pH was adjusted to 6 with 1 M HCl and the solvent was evaporated under a stream of nitrogen. The residue was redissolved in 1:1 mixture of water/acetonitrile and directly purified by preparative HPLC (gradient 20–40%B over 10 min). The fractions were lyophilised to yield TFA salt of **2** (24.5 mg, 0.025 mmol, 79%) as a white solid.

**$^1$H NMR (700 MHz, $D_2O$):** δ 0.84 (3H, s, H-14/15), 0.97 (3H, s, H-14/15), 1.67–1.74 (2H, m, H-2), 1.78–1.83 (1H, m, H-1), 1.83–1.91 (1H, m, H-1), 2.04–2.10 (2H, m, H-2), 2.46 (2H, t, *J* = 6.7 Hz, H-9), 2.64 (1H, sept, *J* = 7.9 Hz, H-3), 2.73 (2H, d, *J* = 7.5 Hz, H-4), 3.01 (2H, t, *J* = 6.5 Hz, H-6), 3.35 (2H, t, *J* = 6.3 Hz, H-7), 3.48 (2H, t, *J* = 6.7 Hz, H-10), 3.62 (1H, dd, *J* = 9.8, 4.7 Hz, H-16), 3.89 (1H, dd, *J* = 9.8, 4.8 Hz, H-16), 4.06 (1H, s, H-12), 4.25–4.32 (2H, m, H-17), 4.63 (1H, quint, *J* = 2.6 Hz, H-18), 4.89 (1H, ddd, *J* = 8.1, 5.0, 3.0 Hz, H-19), 4.92 (1H, ddd, *J* = 6.1, 5.1, 1.0 Hz, H-20), 6.26 (1H, d, *J* = 6.1 Hz, H-21), 8.47 (1H, s, H-25), 8.72 (1H, s, H-22)

**$^{13}$C NMR (176 MHz, $D_2O$):** δ 18.0 (C-1), 18.2 (C-14/15), 20.9 (C-14/15), 27.6 (C-2), 27.9 (C-6), 32.5 (C-3), 35.3 (C-9), 35.4 (C-10), 38.3 (d, *J* = 7.8 Hz, C-13), 38.6 (C-7), 50.2 (C-4), 65.1 (d, *J* = 5.2 Hz, C-17), 71.9 (d, *J* = 6.2 Hz, C-16), 74.01 (d, *J* = 4.9 Hz, C-20), 74.14 (C-12), 74.17 (d, *J* = 5.2 Hz, C-19), 83.6–83.8 (m, C-18), 87.5 (C-21), 118.6 (C-23), 142.6 (C-22), 144.8 (C-25), 148.6 (C-26), 150.0 (C-24), 174.0 (C-8), 174.7 (C-11), 203.7 (C-5)

**$^{31}$P NMR (202 MHz, $D_2O$):** δ -11.4 (br), -10.9 (br), -0.3

**HRMS-ESI (*m/z*):** $[M - H]^-$ calcd for $[C_{27}H_{43}N_7O_{17}P_3S]^-$, 862.1654; found, 862.1635

**3-iodobut-3-enoic acid (3)**

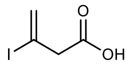

3-Butynoic acid (500 mg, 5.95 mmol) was dissolved in aqueous hydriodic acid (1 mL, 57% w/w) and the mixture was stirred at 70 °C for 6 h. The reaction was quenched by addition of saturated $NaHCO_3$ solution, pH was readjusted with 2 M HCl to 4 and the mixture was extracted with diethyl ether (× 3). The combined organic layers were dried over anhydrous $MgSO_4$, solvent removed *in vacuo* and the residue triturated with petroleum ether. Compound **3** (630 mg, 2.97 mmol, 50%) was obtained as a pale yellow solid.

**TLC:** $R_f$ 0.27 (1:1 diethyl ether/petrol ether, streaks)

**$^1$H NMR (500 MHz, $CDCl_3$):** δ 3.64 (2H, d, *J* = 0.9 Hz), 5.97 (2H, d, *J* = 1.8 Hz), 6.25 (2H, dd, *J* = 2.9, 1.2 Hz), 9.78 (1H, br s)

**$^{13}$C NMR (126 MHz, $CDCl_3$):** δ 50.3, 96.4, 131.3, 175.2

**FT-IR (neat):** 3200–2400 (br m), 1688 (s), 1620 (m)

The data are in accordance with previously reported values.[3]

**3-methyleneoctanoic acid (4)**

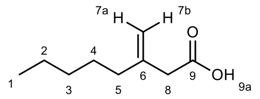

Magnesium shavings (243 mg, 10.0 mmol) were stirred under vacuum for 1 h. Anhydrous diethyl ether (8 mL) was added, followed by 1-bromopentane (1.33 mL, 1.62 g, 10.7 mmol) and the mixture was refluxed for 1 h. The solution was added to a flask charged with dry zinc bromide (2.32 g, 10.3 mmol) and stirred at room temperature overnight. Subsequently, anhydrous *N*,*N*-dimethylformamide was added (6.7 mL) dropwise, followed by dropwise addition of compound **3** (423 mg, 3.33 mmol) pre-mixed with bis(acetonitrile)dichloropalladium (50 mg, 0.19 mmol) in *N*,*N*-dimethylformamide (1.7 mL). The mixture was stirred at 40 °C for 30 min and then at 25 °C overnight. The reaction was quenched by addition of acetic acid (1 mL), the mixture was filtered through Celite and diluted with ethyl acetate. The organic layer was washed with saturated $NH_4Cl$ solution, 5% LiCl solution (× 2) and extracted with 1 M NaOH solution. The aqueous layer was acidified with 1 M HCl and extracted with diethyl ether (× 2). The combined organic layers were dried over anhydrous $MgSO_4$, solvent removed *in vacuo* and the crude material purified using normal-phase chromatography (7:3 petroleum ether/diethyl ether) to yield compound **4** (74 mg, 0.47 mmol, 14%) as a clear oil.

**TLC:** $R_f$ 0.30 (1:1 diethyl ether/petroleum ether, $KMnO_4$)

**$^1$H NMR (700 MHz, $CDCl_3$):** δ 0.89 (3H, t, *J* = 7.1 Hz, H-1), 1.24–1.35 (4H, m, H-2 and H-3), 1.45 (2H, quint, *J* = 7.5 Hz, H-4), 2.12 (2H, t, *J* = 7.7 Hz, H-5), 3.08 (2H, s, H-8), 4.93 (1H, s, H-7a/b), 4.96 (1H, s, H-7a/b), 11.33 (1H, br s, H-9a)

**$^{13}$C NMR (176 MHz, $CDCl_3$):** δ 14.2 (C-1), 22.6 (C-2), 27.2 (C-4), 31.5 (C-3), 36.0 (C-5), 41.7 (C-8), 114.2 (C-7), 142.2 (C-6), 177.7 (C-9)

**FT-IR (neat):** 3200–2600 (br w), 2960 (w), 2930 (m), 2862 (w), 1701 (s), 1643 (m)

**2,5-dioxopyrrolidin-1-yl 3-methyleneoctanoate (5)**

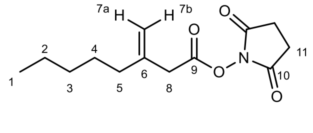

Compound 4 (48 mg, 0.31 mmol) and *N*-hydroxysuccinimide (36 mg, 0.31 mmol) were mixed in dichloromethane (0.8 mL). A solution of *N*,*N*′-dicyclohexylcarbodiimide (76 mg, 0.37 mmol) in

dichloromethane (0.4 mL) was added dropwise and the mixture was stirred overnight at room temperature. The reaction mixture was filtered through Celite and the filtrate concentrated *in vacuo*. The crude material was purified by normal-phase chromatography (gradient of ethyl acetate 0–50% in petroleum ether) to yield **5** (70 mg, 0.28 mmol, 89%) as a clear oil.

**TLC:** $R_f$ 0.33 (3:7 ethyl acetate/petroleum ether)

**$^1$H NMR (700 MHz, $CDCl_3$):** δ 0.89 (3H, t, *J* = 7.0 Hz, H-1), 1.25–1.36 (4H, m, H-2 and H-3), 1.47 (2H, quint, *J* = 7.5 Hz, H-4), 2.16 (2H, t, *J* = 7.7 Hz, H-5), 2.83 (4H, br d, *J* = 8.3 Hz, H-11), 3.31 (2H, s, H-8), 5.03 (1H, s, H-7a/b), 5.06 (1H, s, H-7a/b)

**$^{13}$C NMR (176 MHz, $CDCl_3$):** δ 14.1 (C-1), 22.6 (C-2), 25.7 (C-11), 27.1 (C-4), 31.5 (C-3), 35.8 (C-5), 38.6 (C-8), 115.1 (C-7), 140.4 (C-6), 166.7 (C-9), 169.2 (C-10)

**FT-IR (neat):** 2959 (w), 2932 (w), 2862 (w), 1816 (w), 1786 (w), 1735 (s), 1649 (w)

**3-methyleneoctanoyl Coenzyme A (6)**

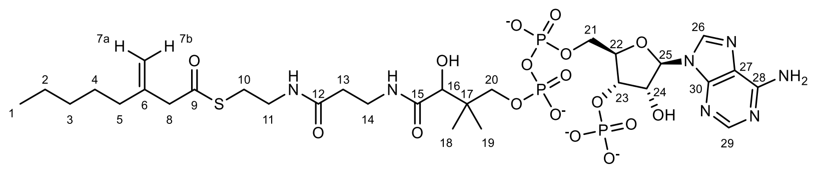

Compound **5** (35 mg, 0.14 mmol) was dissolved in acetone (0.4 mL), **CoA-SH** (26 mg, 0.032 mmol) was dissolved in water (0.4 mL) and $NaHCO_3$ (40 mg, 0.48 mmol) was dissolved in water (1.2 mL). The **CoA-SH** and $NaHCO_3$ solutions were combined at 0 °C and stirred for 10 min. Subsequently, the compound **5** solution was added dropwise and more acetone added dropwise until a clear homogeneous solution formed. The mixture was stirred at 0 °C overnight (the solution became opaque), after which the pH was adjusted to 6 with 1 M HCl (solution cleared up) and the solvent was evaporated under a stream of nitrogen. The residue was redissolved in 1:1 mixture of water/acetonitrile and directly purified by preparative HPLC (gradient 35–50%B over 10 min). The fractions were lyophilised to yield TFA salt of **6** (21.4 mg, 0.021 mmol, 66%) as a white solid.

**$^1$H NMR (700 MHz, $D_2O$):** δ 0.84 (3H, s, H-18/19), 0.85 (3H, t, *J* = 7.2 Hz, H-1), 0.97 (3H, s, H-18/19), 1.21–1.26 (2H, m, H-3), 1.26–1.30 (2H, m, H-2), 1.41 (2H, quint, *J* = 7.5 Hz, H-4), 2.04 (2H, t, *J* = 7.6 Hz, H-5), 2.45 (2H, t, *J* = 6.8 Hz, H-13), 3.04 (2H, t, *J* = 6.3 Hz, H-10), 3.37† (2H, s, H-8), 3.37† (2H, t, *J* = 6.1 Hz, H-11), 3.48 (2H, t, *J* = 7.0 Hz, H-14), 3.62 (1H, dd, *J* = 9.7, 4.7 Hz, H-20), 3.89 (1H, dd, *J* = 9.7, 4.7 Hz, H-20), 4.06 (1H, s, H-16), 4.25–4.31 (2H, m, H-21), 4.63 (1H, quint, *J* = 2.5 Hz, H-22), 4.88–4.91 (1H, m, H-23), 4.91–4.93 (1H, m, H-24), 4.95 (1H, s, H-7a/b), 5.01 (1H, q, *J* = 1.3 Hz, H-7a/b), 6.26 (1H, d, *J* = 5.9 Hz, H-25), 8.48 (1H, s, H-29), 8.72 (1H, s, H-26)

†Peaks overlap with a total integral of 4H.

**$^{13}$C NMR (176 MHz, $D_2O$):** δ 13.3 (C-1), 18.2 (C-18/19), 20.9 (C-18/19), 21.8 (C-2), 26.4 (C-4), 28.2 (C-10), 30.6 (C-3), 35.2 (C-5), 35.37 (C-13), 35.42 (C-14), 38.4 (d, *J* = 8.1 Hz, C-17), 38.5 (C-11), 50.5 (C-8), 65.1 (d, *J* = 4.6 Hz, C-21), 71.9 (d, *J* = 5.9 Hz, C-20), 74.0 (d, *J* = 4.9 Hz, C-24), 74.1 (C-16), 74.2 (d, *J* = 5.6 Hz, C-23), 83.6–83.7 (m, C-22), 87.5 (C-25), 114.6 (C-7), 118.6 (C-27), 142.6 (C-26), 143.4 (C-6), 144.7 (C-29), 148.6 (C-30), 150.0 (C-28), 173.9 (C-12), 174.7 (C-15), 202.3 (C-9)

**$^{31}$P NMR (202 MHz, $D_2O$):** δ -11.4 (br), -10.9 (br), -0.3

**HRMS-ESI (*m/z*):** $[M - H]^-$ calcd for $[C_{30}H_{49}N_7O_{17}P_3S]^-$, 904.2124; found, 904.2123

**Purity of CoA conjugates**

The purity of CoA conjugates **2** and **6** was assessed using analytical HPLC. Two gradients: 5–95%B and 20–60%B were used. The HPLC traces are shown below with the retention time and percentage area under curve of the peaks highlighted.

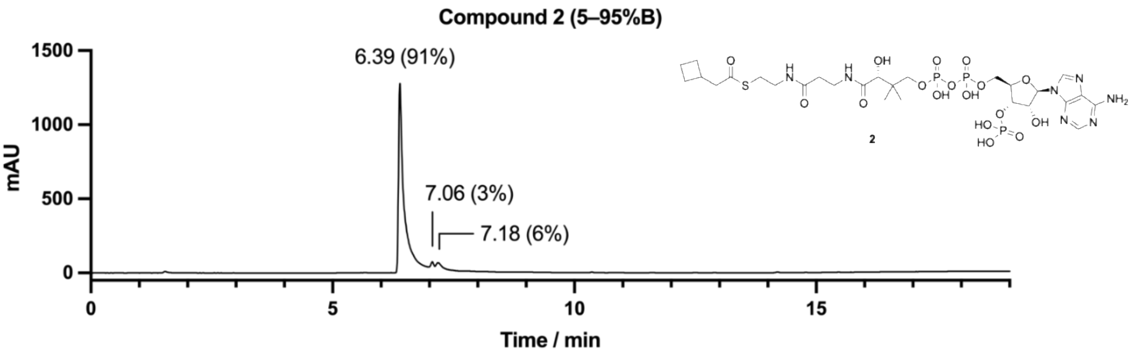


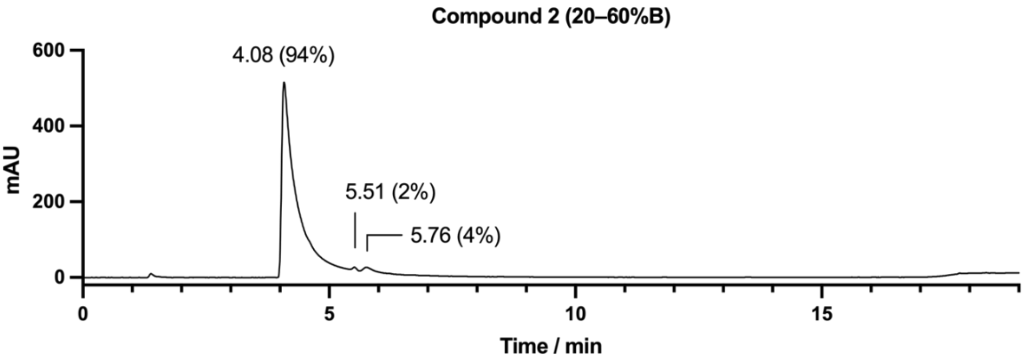

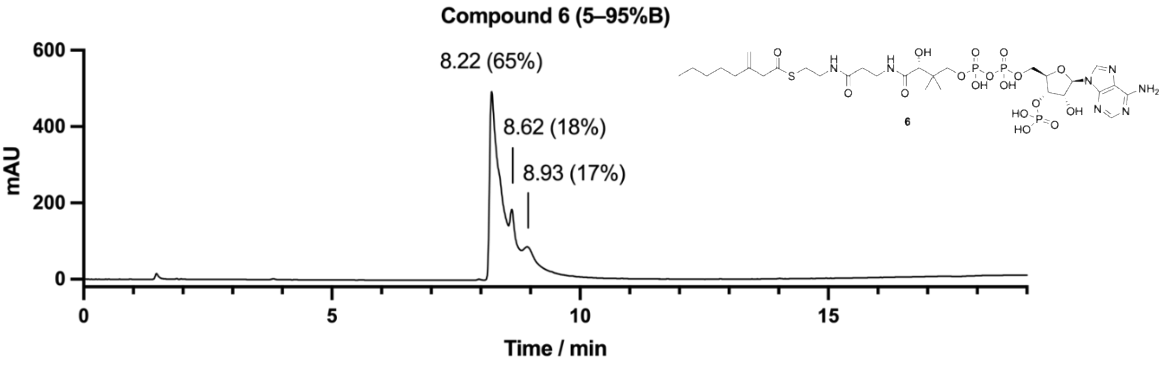
Compound 6 (5–95%B)
8.22 (65%)
8.62 (18%)
8.93 (17%)
mAU
Time / min
6

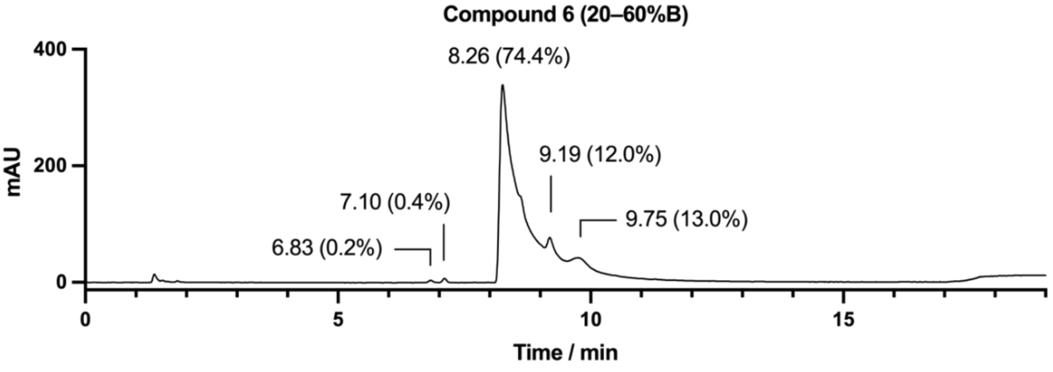
Compound 6 (20–60%B)
8.26 (74.4%)
9.19 (12.0%)
7.10 (0.4%)
6.83 (0.2%)
9.75 (13.0%)
mAU
Time / min

## NMR spectra

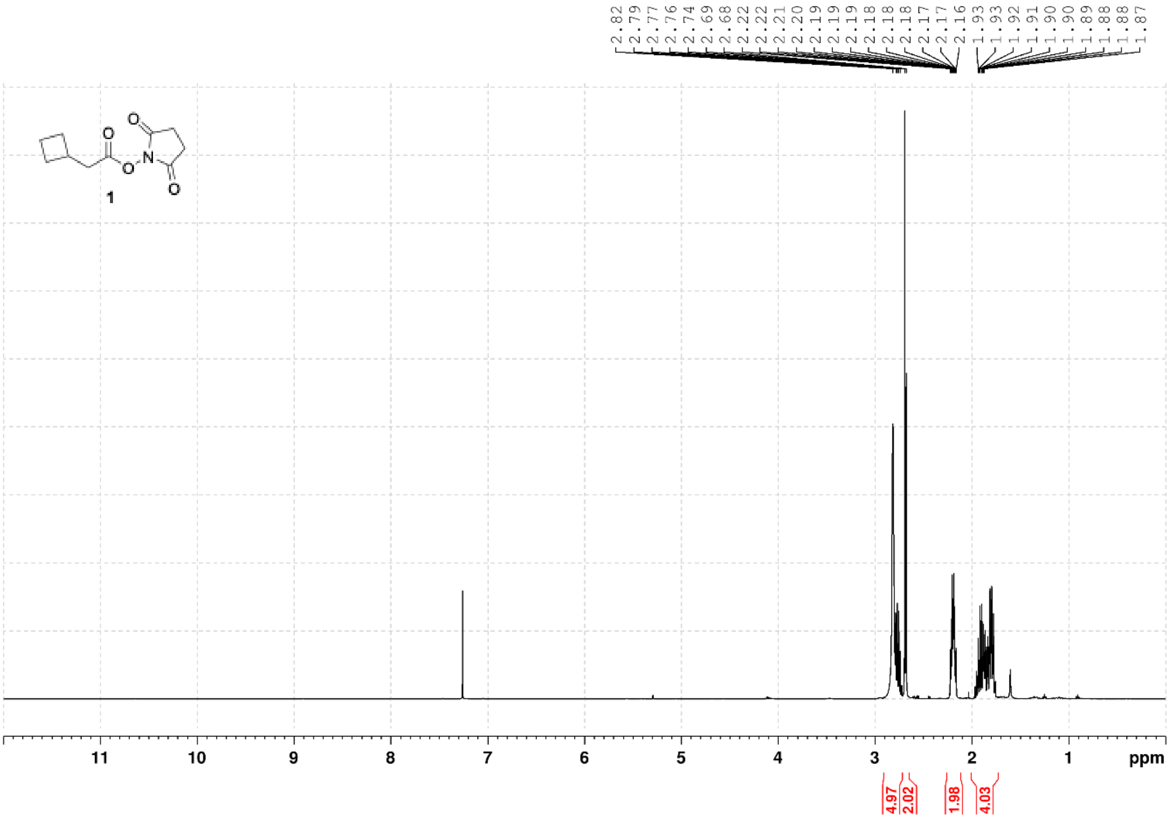
2.82
2.79
2.77
2.76
2.74
2.69
2.68
2.22
2.22
2.21
2.20
2.19
2.19
2.19
2.18
2.18
2.18
2.17
2.17
2.16
1.93
1.93
1.92
1.91
1.90
1.90
1.89
1.88
1.88
1.87
1
ppm
4.97
2.02
1.98
4.03

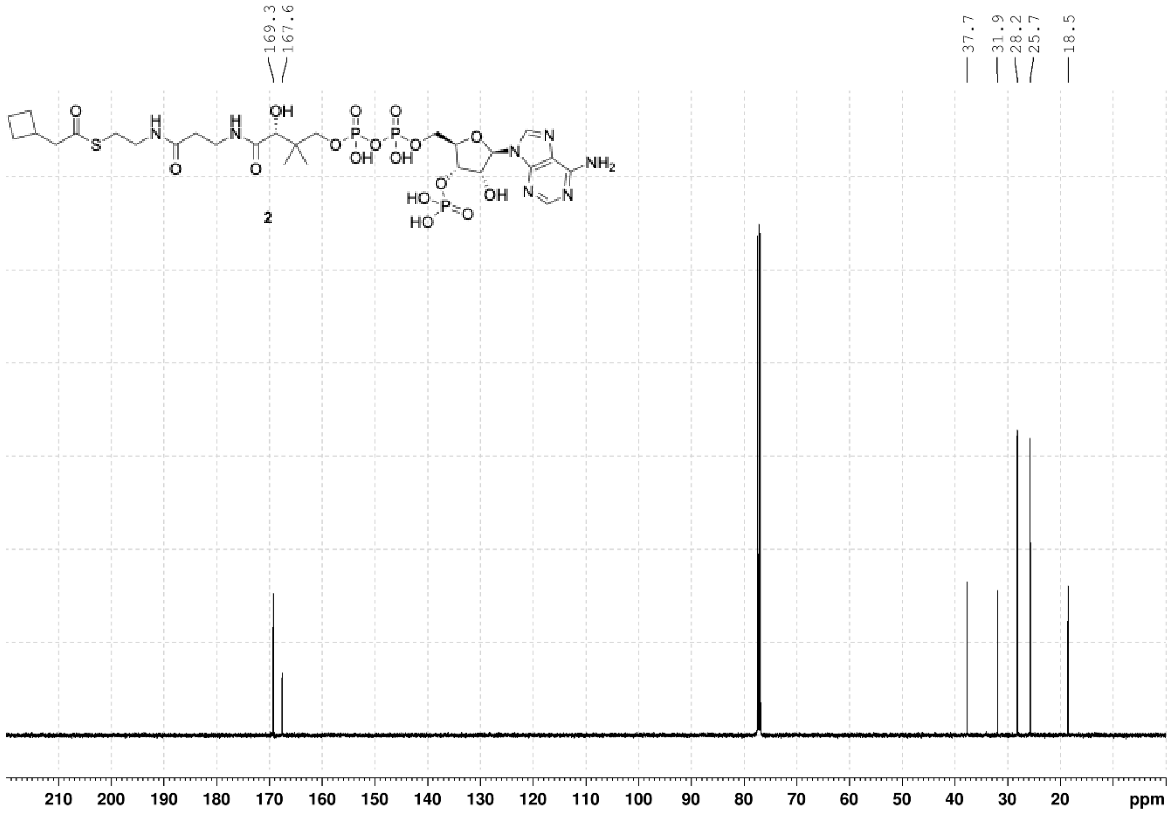
169.3
167.6
37.7
31.9
28.2
25.7
18.5
2
210 200 190 180 170 160 150 140 130 120 110 100 90 80 70 60 50 40 30 20 ppm

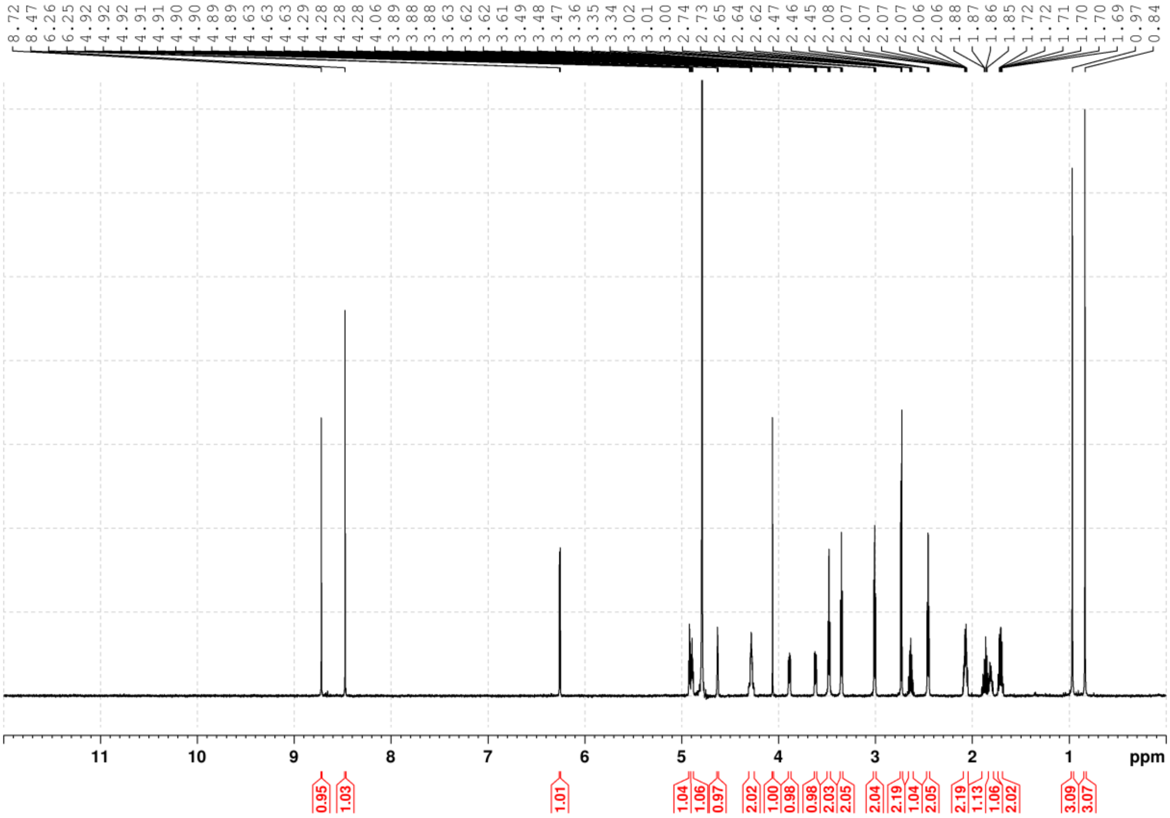
8.72 8.47 6.26 6.25 4.92 4.92 4.91 4.91 4.90 4.90 4.89 4.89 4.63 4.63 4.63 4.29 4.28 4.28 4.28 4.06 3.89 3.88 3.88 3.63 3.62 3.61 3.49 3.48 3.47 3.36 3.35 3.34 3.02 3.01 3.00 2.74 2.73 2.65 2.64 2.62 2.47 2.46 2.45 2.08 2.07 2.07 2.07 2.07 2.06 2.06 1.88 1.87 1.86 1.85 1.72 1.72 1.71 1.70 1.70 1.69 0.97 0.84
11 10 9 8 7 6 5 4 3 2 1 ppm
0.95 1.03 1.01 1.04 1.06 0.97 2.02 1.00 0.98 0.98 2.03 2.05 2.04 2.19 1.04 2.05 2.19 1.13 1.06 2.02 3.09 3.07

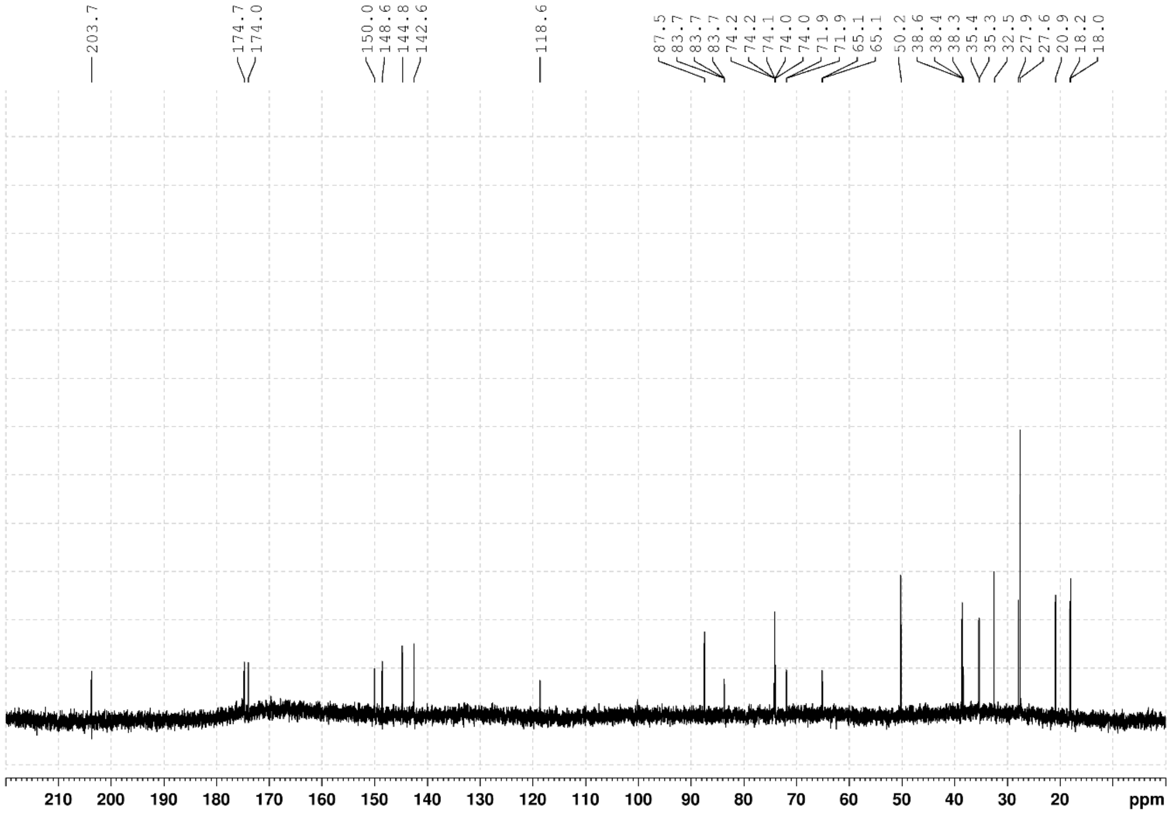

203.7
174.7
174.0
150.0
148.6
144.8
142.6
118.6
87.5
83.7
83.7
83.7
74.2
74.2
74.1
74.0
74.0
71.9
71.9
65.1
65.1
50.2
38.6
38.4
38.3
35.4
35.3
32.5
27.9
27.6
20.9
18.2
18.0
210 200 190 180 170 160 150 140 130 120 110 100 90 80 70 60 50 40 30 20 ppm

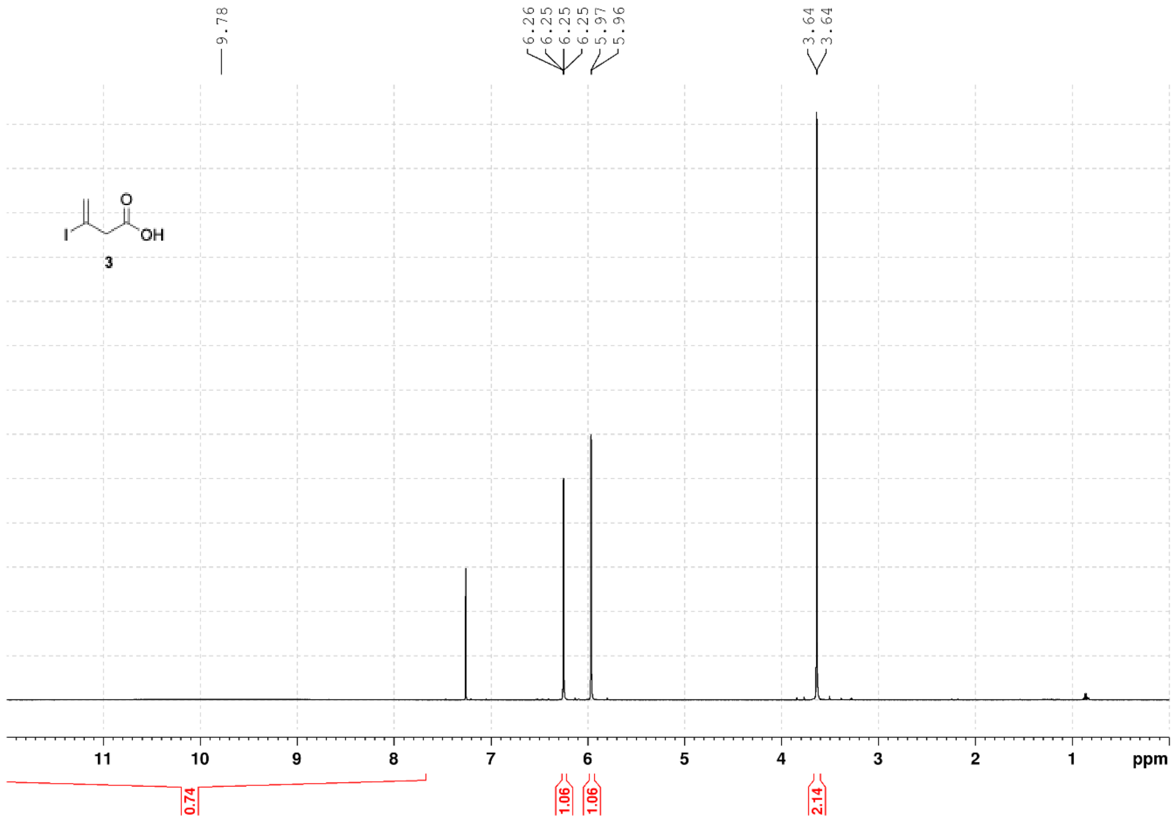
9.78
6.26
6.25
6.25
6.25
5.97
5.96
3.64
3.64
I
O
OH
3
11
10
9
8
7
6
5
4
3
2
1
ppm
0.74
1.06
1.06
2.14

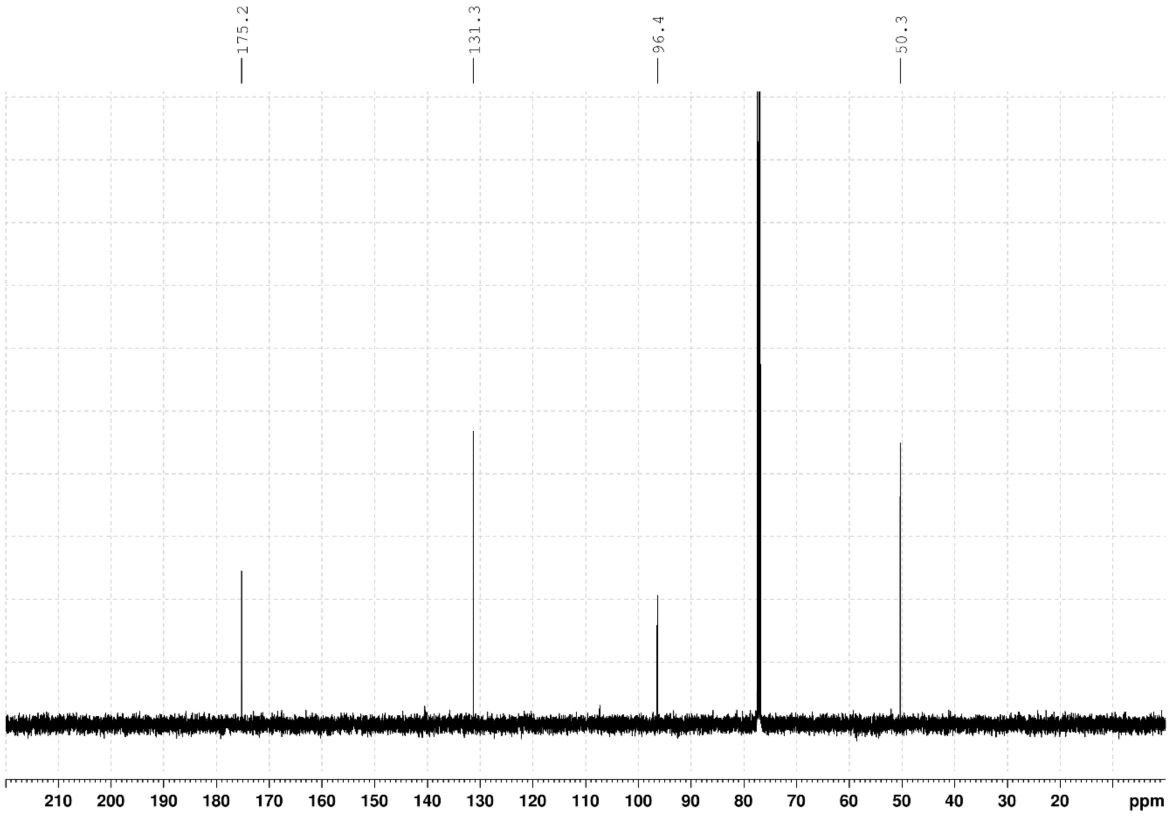
175.2
131.3
96.4
50.3
210
200
190
180
170
160
150
140
130
120
110
100
90
80
70
60
50
40
30
20
ppm

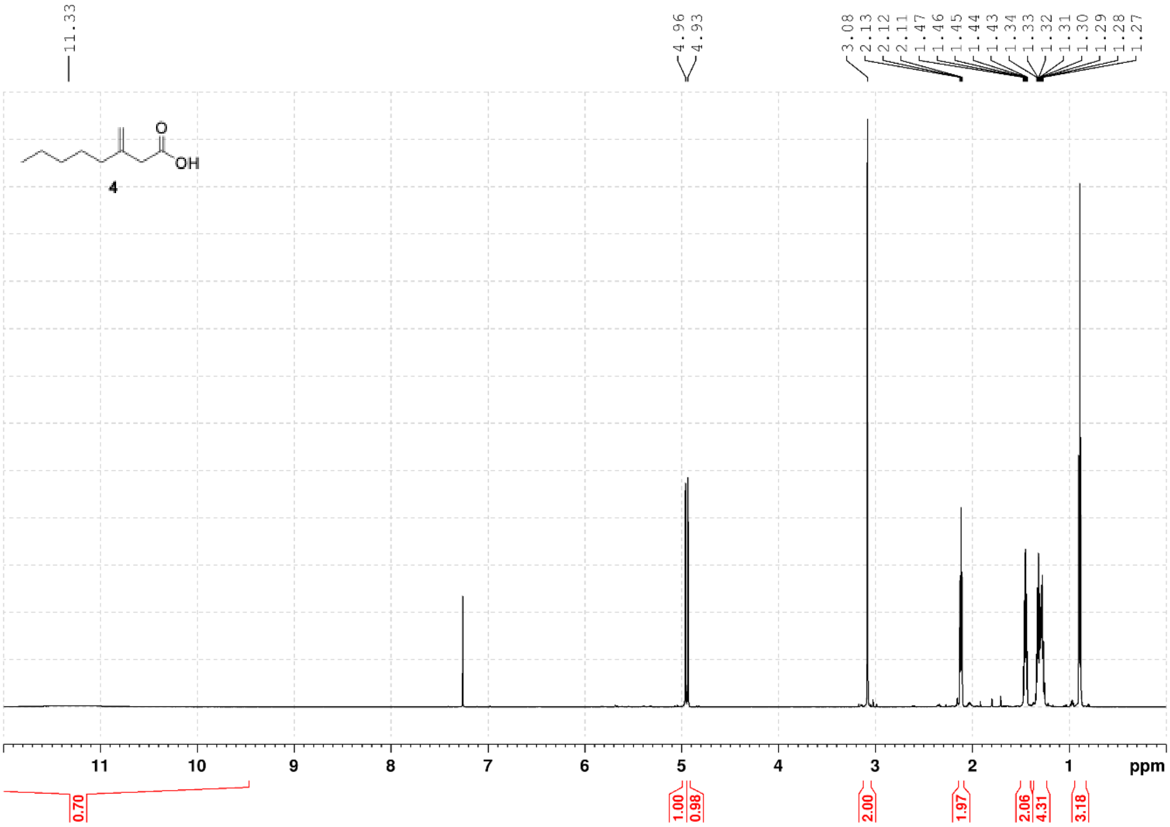

11.33
4.96
4.93
3.08
2.13
2.12
2.11
1.47
1.46
1.45
1.44
1.43
1.34
1.33
1.32
1.31
1.30
1.29
1.28
1.27
O
OH
4
11 10 9 8 7 6 5 4 3 2 1 ppm
0.70
1.00
0.98
2.00
1.97
2.06
4.31
3.18


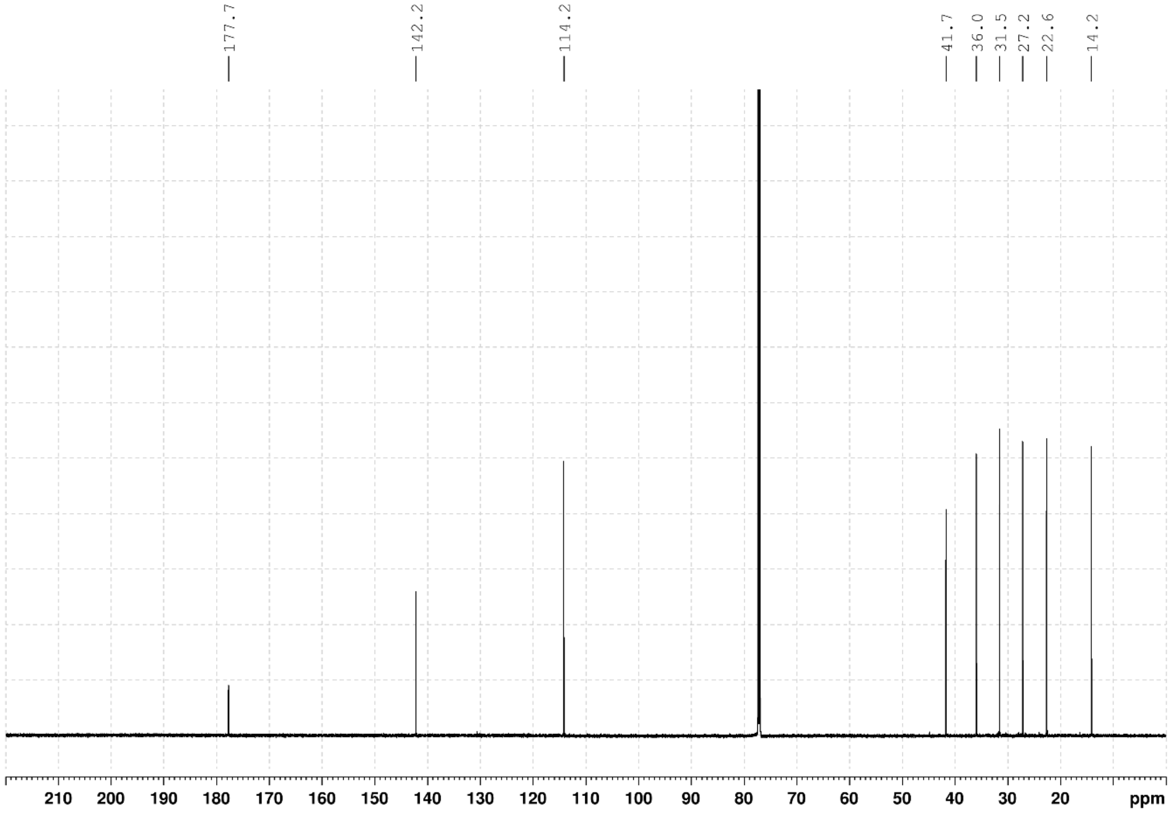

177.7
142.2
114.2
41.7
36.0
31.5
27.2
22.6
14.2
210 200 190 180 170 160 150 140 130 120 110 100 90 80 70 60 50 40 30 20 ppm

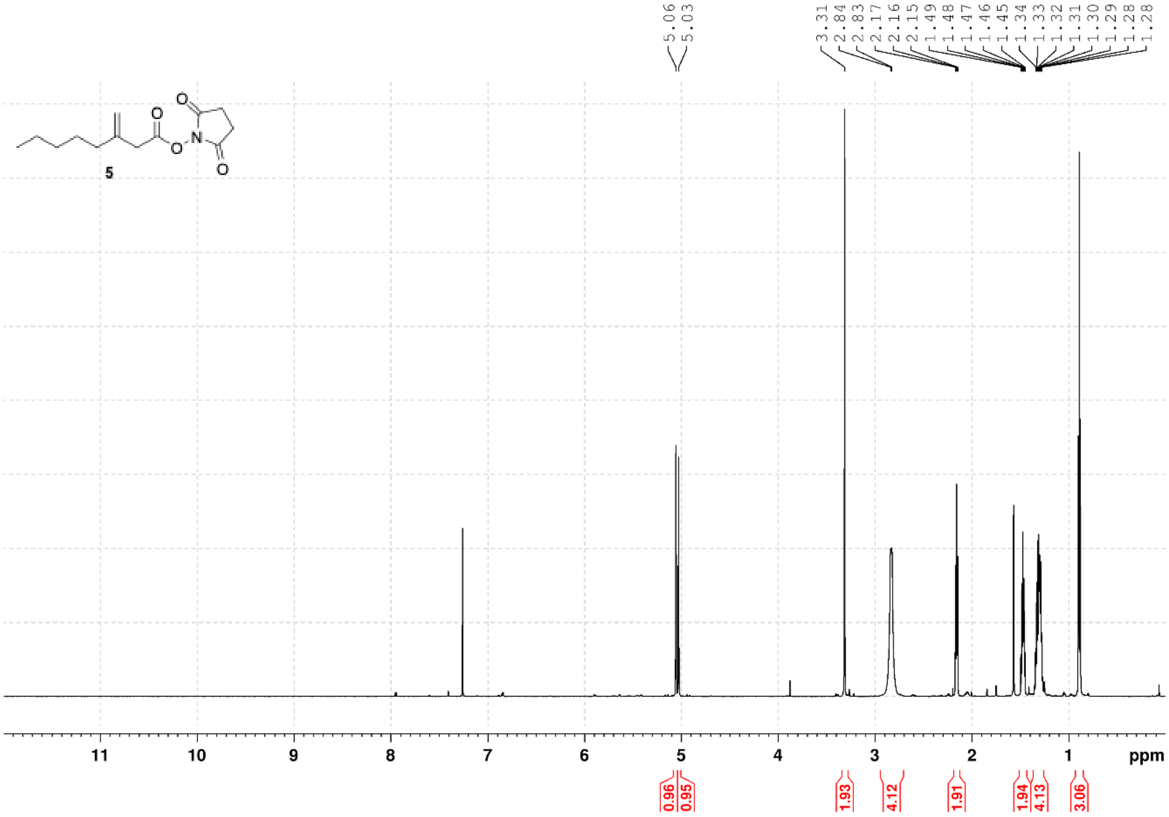

5
5.06
5.03
3.31
2.84
2.83
2.17
2.16
2.15
1.49
1.48
1.47
1.46
1.45
1.34
1.33
1.32
1.31
1.30
1.29
1.28
1.28
11 10 9 8 7 6 5 4 3 2 1 ppm
0.96
0.95
1.93
4.12
1.91
1.94
4.13
3.06


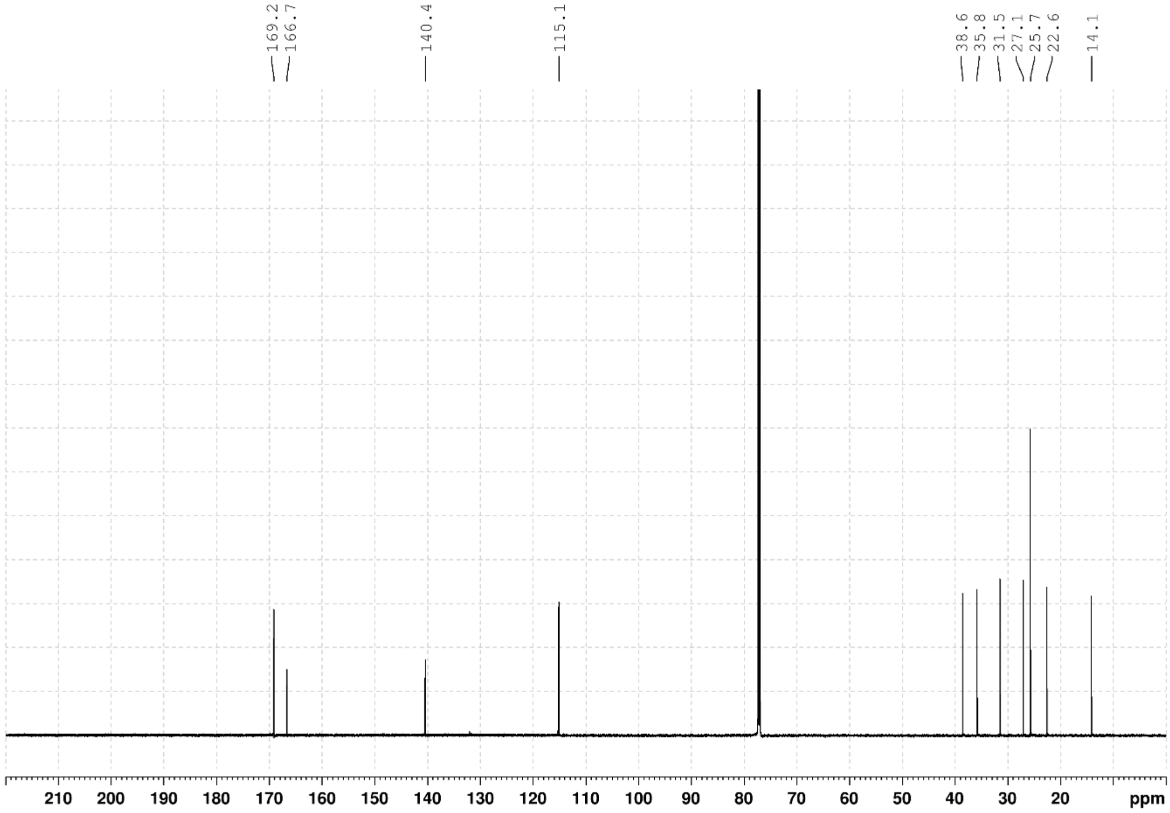

169.2
166.7
140.4
115.1
38.6
35.8
31.5
27.1
25.7
22.6
14.1
210 200 190 180 170 160 150 140 130 120 110 100 90 80 70 60 50 40 30 20 ppm

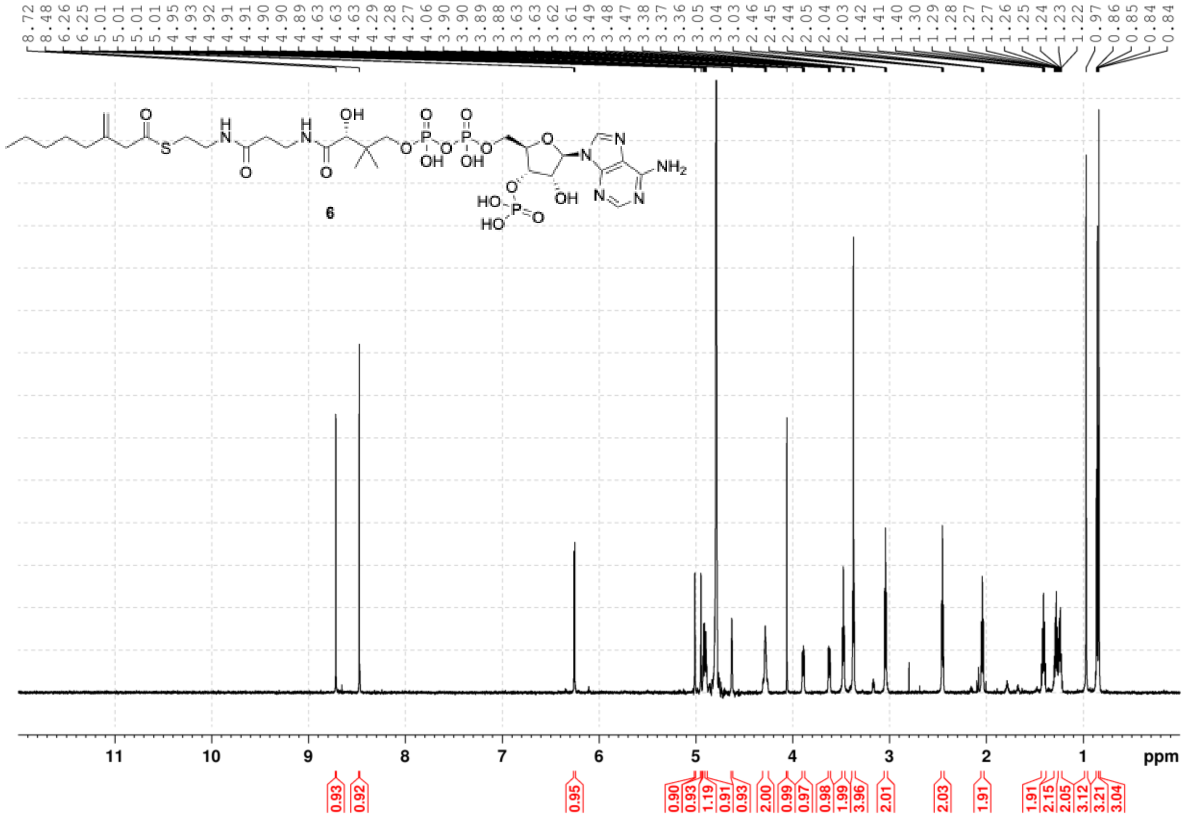
8.72
8.48
6.26
6.25
5.01
5.01
5.01
5.01
4.95
4.93
4.92
4.91
4.91
4.90
4.90
4.89
4.63
4.63
4.29
4.28
4.27
4.06
3.90
3.90
3.89
3.88
3.63
3.63
3.62
3.61
3.49
3.48
3.47
3.38
3.37
3.36
3.05
3.04
3.03
2.46
2.45
2.44
2.05
2.04
2.03
1.42
1.41
1.40
1.30
1.29
1.28
1.27
1.27
1.26
1.25
1.24
1.23
1.22
0.97
0.86
0.85
0.84
0.84
6
11
10
9
8
7
6
5
4
3
2
1
ppm
0.93
0.92
0.95
0.90
0.93
1.19
0.91
0.93
2.00
0.99
0.97
0.98
1.99
3.96
2.01
2.03
1.91
1.91
2.15
2.05
3.12
3.21
3.04

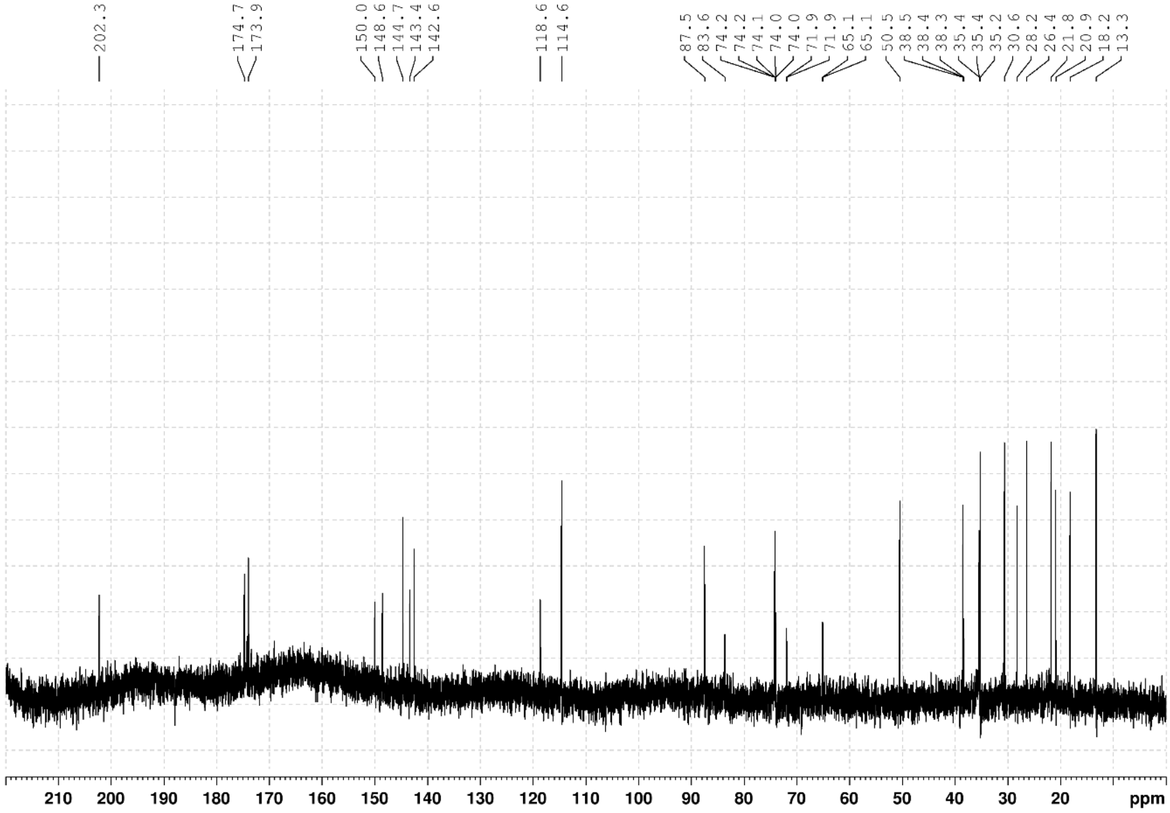
202.3
174.7
173.9
150.0
148.6
144.7
143.4
142.6
118.6
114.6
87.5
83.6
74.2
74.1
74.0
74.0
71.9
71.9
65.1
50.5
38.5
38.4
38.3
35.4
35.4
35.2
30.6
28.2
26.4
21.8
20.9
18.2
13.3
210
200
190
180
170
160
150
140
130
120
110
100
90
80
70
60
50
40
30
20
ppm

**Supplementary references**


1 Ghisla, S. & Thorpe, C. Acyl-CoA dehydrogenases. *European Journal of Biochemistry* **271**, 494-508 (2004).

2 Gao, B.-W., Wang, X.-H., Liu, X., Shi, S.-P. & Tu, P.-F. Rapid preparation of (methyl)malonyl coenzyme A and enzymatic formation of unusual polyketides by type III polyketide synthase from Aquilaria sinensis. *Bioorganic & Medicinal Chemistry Letters* **25**, 1279-1283 (2015).

3 Abarbri, M., Parrain, J.-L., Kitamura, M., Noyori, R. & Duchêne, A. Palladium-Catalyzed Cross-Coupling of 3-Iodobut-3-enoic Acid with Organometallic Reagents. Synthesis of 3-Substituted But-3-enoic Acids. *The Journal of Organic Chemistry* **65**, 7475-7478 (2000).
