# Supplementary material for: Pseudomonas aeruginosa acyl-CoA dehydrogenases and structure-guided inversion of their substrate specificity
Source: Nat Commun. 2025 Mar 8;16:2334. doi: 10.1038/s41467-025-57532-z (PMC11890623; doi:10.1038/s41467-025-57532-z)
Supplement: Supplementary file 5 — Transparent Peer Review file [file 41467_2025_57532_MOESM5_ESM.pdf]

nature portfolio

Peer Review File

Pseudomonas aeruginosa acyl-CoA dehydrogenases and structure-guided inversion of their substrate specificity.

Corresponding Author: Professor Martin Welch

This file contains all reviewer reports in order by version, followed by all author rebuttals in order by version.

Version 1:

Reviewer comments:

Reviewer #1

(Remarks to the Author)
The manuscript "How to fit a round peg in a square hole: Pseudomonas aeruginosa acyl-CoA dehydrogenases and structure-guided inversion of their substrate specificity" by Meng Wang et al. characterizes the structure and function of two acyl-CoA dehydrogenases essential for p aeruginosa to grow on fatty acids. The authors used tandem mass-tag proteomics to find out that two acyl-CoA dehydrogenases, out of the 22 found, were highly induced when P. aeruginosa was grown on fatty acids (octanoate, palmitate or oleate) compared to when grown on glucose. Moreover, mutations in these dehydrogenases impaired P. aeruginosa grown on fatty acids. One of these dehydrogenases, FadE1, showed preference for long-chain fatty acids while the other, FadE2, for medium-chain fatty acids. The structure of both deshydrogenesases, as well as some mutants, were solved to identify, in combination with mutagenesis, the residues involved in their different specificity. This also led to the identification of potential inhibitors that could target P. aeruginosa, but not human cells.
Although this is a serious, well-written and interesting article, in its present state it lacks the novelty necessary for it to be published in Nature communications.
Major issues
1- To avoid misinterpretations inherent of the Proteomics technique this study should be complemented with RNAseq analysis of the strain grown on fatty acids vs glucose. The stranded mRNA analysis could also help in the identification of the different TSS and promoters. The authors state in the discussion section that the PA0506, PA0507, PA0508 is not predicted to be an operon, but by RNAseq analysis they could easily check this fact.
2- There is an ample discussion in the article about the possible role of PsrA and PvrA in regulating FadE1 and FadE2 expression. The presence of the consensus sequences of PsrA and PvrA binding sites in the promoter region of the genes coding for these dehydrogenases should be analyzed in silico or even in vitro by EMSA or in vivo by transcriptomic or proteomic analysis of PsrA or PvrA P. aeruginosa mutant strains.
Other issues.
-Page 6. Line 136. Two FadE1 and FadE2 share 53% identity with one another, but almost none with E. coli FadE. Please show the identity percentage between E. coli FadE and FadE1 and FadE2.
-Page 8. Line 180. "Other FadE homologs were upregulated …" It would be good to see the MSA of these FadE proteins with FadE1 and FadE2, highlighting the differences and even predicting the fatty acids used in each case. In fact a MSA and classification of all the 22 identified acyl-CoA dehydrogenases in P aeruginosa could be helpful to understand their differential role.
-Page 11. When comparing FadE1 and FadE2 structures between each other and with other homologs, Cα RMSD should be provided.
-Page 13. FadE1^{E441A} bound to C16-CoA structure should also be compared with wtFadE1 (presumably bound to octanoyl-CoA) structure.
-Page 15. Line 306. Is there any extra electron density in the active site of FadE2 triple mutant?
-In general, the quality of the figures describing the structures should be improved.

Reviewer #2

(Remarks to the Author)

This manuscript presented a study to elucidate the functions of predicted acyl-CoA dehydrogenase. The authors first applied a proteomics approach to identify 2 predicted FadE proteins that were overexpressed in PA grown in media containing different lengths of fatty acids as sole carbon source. This study is very interesting and the application of TMT-16 plex labelled proteomics was apt and elegant.

My co-reviewer and I were specifically asked to focus and comment on the proteomics section of this manuscript. In general, we did not find major flaws. However, we did suggest some improvement to the reporting that can enhance the reporting accuracy. Our comments are as follows:

Major points
1. Since the authors used tandem mass tags (TMT-16plex) approach for protein quantification, it is important to evaluate the labeling efficiency. Can authors provide this information? Also, please provide sample information for each tag channel.

2. Further, they mentioned, "Sixteen pairs of dried fractions were analyzed by LC-MS/MS as previously described [6]…" How did they derive these 16 pairs of dried fractions? I could not obtain this information from reference [6] provided: Dolan Stephen K, Kohlstedt M, Trigg S, Vallejo Ramirez P, Kaminski Clemens F, Wittmann C, et al. Contextual Flexibility in Pseudomonas aeruginosa Central Carbon Metabolism during Growth in Single Carbon Sources. mBio. 2020;11:e02684-19. Though I got this information from the "Sample Processing Protocol" from the PRIDE database as follows: "Desalted peptides were dried and resuspended in 100 uL of 20mM ammonium formate (pH 10.0) then mixed with 4% (v/v) acetonitrile for fractionation. Fractionation was performed using Waters Acquity UPLC (Waters Corporation, Milford MA) equipped with an Acquity bridged ethyl hybrid C18 UPLC column (Waters; 2.1 mm i.d. x 150 mm, 1.7 µm particle size). Peptides were profiled at a flow rate of 0.25 mL/min with a linear gradient of 5-60% acetonitrile with 20 mM ammonium formate (pH 10.0) over 60 minutes. Thirty-two fractions were collected and reduced to dryness by vacuum centrifugation. Fractions were combined into 16 pairs (i.e. the first fraction with the middle fraction etc) and were analyzed by LC-MS/MS", this should be included in the method session. The authors should have described the protocol early in the supporting information. Furthermore, they should describe clearly how they have concatenated the fractions, such as combining fractions 1 and 17, 2 and 18, etc.

3. Certain parameters used in this work and that of reference [6] were slightly different, such as the flow rate of the basic reverse phase run. This should have been specified for the sake of reporting accuracy.

4. I did not find the experimental details of LC-MS/MS analysis and data processing. These are crucial for evaluating the reliability and data quality of proteomic analysis. Specific details about the instrument parameters, such as the column type, gradient, flow rate, and MS settings (e.g., resolution, scan range, collision energy), are required to ensure reproducibility. How many sequence queries are in your database, and where does your database come from? How is the data distribution with or without normalization? How does normalization proceed? How did the authors calculate the protein abundance using a fractionation dataset? Which software and search parameters did authors use for data processing? For example, the tolerance for MS1, MS2, and MS3, the fixed and variable modifications, the FDR at PSM, peptide, and protein level of target-decoy database searching, the number of missed cleavages, and the criteria of minimum peptide length should all be disclosed.

5. On page 6: "Of the 2640 proteins that were common to all of the samples, 19 showed increased abundance ($\log2FC \geq 1.0$, adjusted p-value ≤ 0.01) on all three fatty acid substrates (cf. glucose) and 57 showed decreased abundance (Figure S2A, B)". Does this mean the author had ensured that all replicates have valid values? Any protein that has a missing value in even one replicate is discarded without applying imputation? Isn't that too stringent? Would that result in the accidental removal of any of the other 19 predicted acyl-CoA dehydrogenases (FadE) that might have been differentially expressed from this dataset?

6. It is unclear what the input of PCA in Figure S2C is: the total proteome or the significantly changed proteins. The meaning is quite different based on the input dataset. Besides, there is no related description in the manuscript.

7. Figure 1A-C: There were other predicted proteins that were overexpressed in C8:0 (PA4435 & PA1631), C16:0 (PA4435) and C18d (PA4435). Are any of these proteins related to fatty acid metabolism?

Minor points:
1. On page S19, the authors mentioned using Bio-rad protein assay reagent. Is that Bradford assay or other types of assays for protein concentration estimation?

2. The authors should also preferably upload the protein FASTA used in the PRIDE repository and specify the version and date of download of such a database from UniProt.

3. For subsequent statistical analysis, the authors mentioned, "Following global normalization of the data (using common quality thresholds), the data were analyzed using limma with a mean reference model and the lmFit and Bayes functions." This information is too simplified. The authors should have included the parameters or algorithms used. For example, how did they pre-clean, impute, or normalize their data? Is there any minimal number of missing values?
4. To be reader-friendly, the proteins involved in glucose or fatty acid metabolism in Fig1A-1D should be labeled with a different color or symbol.

Reviewer #3

(Remarks to the Author)
The study by Wang et al. characterizes two putative acyl-CoA dehydrogenases from Pseudomonas aeruginosa, which is a significant infection in cystic fibrosis patients. Crystal structures and biochemical assays support the identification of selectivity filters that define the substrate selectivity profile for each enzyme. Interesting speculative evolutionary analysis indicates one of the enzymes has reached a peak in local adaptive fitness whereas the other is still evolving toward its peak. The characterization is interesting but there are several limitations that dampen the overall impact of the study and make it better suited for a specialty journal.
Major comments:
1. In vivo validation of the enzymatic analysis. Measurement of the CoA pools via metabolomics is necessary to confirm the function of the putative acyl-CoA dehydrogenases in vivo. If the identities are correct, then the mutants (fadE1 and fadE2 KOs) will alter the CoA pools and provide an explanation for the phenotypes – toxicity from CoA sequestration.
2. Biological validation of the in silico analysis. FadE1 and FadE2 biochemical inhibition by the thioester inhibitors is primarily needed to confirm the enzymes are sensitive to the inhibitors, and secondarily to validate their homolog selectivity. More importantly, drug (or perhaps prodrug) toxicity to Pseudomonas aeruginosa must be demonstrated to establish the viability for this antimicrobial strategy.
3. Infection phenotype for mutants. There is no evidence that inhibiting PA0506 and/or PA0508 will have any impact on Pseudomonas aeruginosa infection. Although there is a strong phenotype well the cells are grown in vitro, it is unclear how the other 20+ putative acyl-CoA dehydrogenases compensate (or don't) in the context of infection. Their potential redundant functions may be induced under conditions not tested in this study. Thus, whether inhibition of these enzymes have any therapeutic promise for this infection is uncertain.
Minor comments:
1. A schematic of the b-oxidation process that illustrates where FadE fits in would benefit the reader and nonexpert who is unfamiliar with the reactions.
2. Can the differences between FadE1 and FadE2 be used to predict the substrate selectivity of the other 20+ Pseudomonas aeruginosa acyl-CoA dehydrogenases?
3. How many of the other putative acyl-CoA dehydrogenases are in gene clusters (e.g., polyketide synthesis)? Presumably this would suggest they are not primarily engaged in b-oxidation and not redundant for FadE1 or FadE2.
4. Figure 1. The genes of interest should be emphasized (bolded?) to better draw the reader's attention to them.
5. An explanation must be provided for rationalizing the resolutions of the crystal structures and why the authors avoided statistically sensible cutoffs for resolution (e.g., I/sigma = 2). Although there is good multiplicity, completeness, and satisfactory CC1/2 in the highest shell, the R-factors for data at "this resolution" are unacceptable, particularly for the Apo FadE2 structure. The separation of the Rwork and Rfree for Apo FadE2 indicates over refinement and 1.94 Å resolution is unliklely. The data should be reprocessed at a lower resolution.

Reviewer #4

(Remarks to the Author)
I co-reviewed this manuscript with one of the reviewers who provided the listed reports. This is part of the Nature Communications initiative to facilitate training in peer review and to provide appropriate recognition for Early Career Researchers who co-review manuscripts.

Version 2:

Reviewer comments:

Reviewer #1

(Remarks to the Author)
After addresing the issues raised by the referees, I recommend that the article should be published in the present form.

Reviewer #2

(Remarks to the Author)
I went through the revised manuscript point by point. The authors have satisfactorily addressed every point and comment that I raised in the my previous review. I am happy with the answers and revision provided. I recommend to accept this revised manuscript.

Reviewer #3

(Remarks to the Author)
The revised manuscript by Wang et al. characterizes two putative acyl-CoA dehydrogenases from Pseudomonas aeruginosa, a clinically significant pathogen in cystic fibrosis patients. Through crystal structures, infection experiments, and biochemical assays, the authors identify selectivity filters that define the substrate specificity of each enzyme and their relevance in Pseudomonas infection. Additionally, a fascinating evolutionary analysis suggests that one enzyme has reached a peak in local adaptive fitness, while the other appears to be evolving toward its fitness peak.

The authors have thoroughly addressed the concerns raised in the previous review, significantly enhancing the study's clarity and impact. I enthusiastically support the publication of this outstanding work.

Reviewer #4

(Remarks to the Author)
I co-reviewed this manuscript with one of the reviewers who provided the listed reports. This is part of the Nature Communications initiative to facilitate training in peer review and to provide appropriate recognition for Early Career Researchers who co-review manuscripts.

Open Access This Peer Review File is licensed under a Creative Commons Attribution 4.0 International License, which permits use, sharing, adaptation, distribution and reproduction in any medium or format, as long as you give appropriate credit to the original author(s) and the source, provide a link to the Creative Commons license, and indicate if changes were made.
In cases where reviewers are anonymous, credit should be given to 'Anonymous Referee' and the source.
The images or other third party material in this Peer Review File are included in the article's Creative Commons license, unless indicated otherwise in a credit line to the material. If material is not included in the article's Creative Commons license and your intended use is not permitted by statutory regulation or exceeds the permitted use, you will need to obtain permission directly from the copyright holder.
To view a copy of this license, visit https://creativecommons.org/licenses/by/4.0/

Pre-amble. Our apologies for the time taken to turn this around; some of the experiments were demanding (novel chemical synthesis, setting up of collaborations for the Galleria work, optimizing growth for metabolomics work, etc). The Nat Comms office has been kept informed of progress and anticipated re-submission dates.

We thank the reviewers for their comments, and for the time they took to go over the paper. The MS has now improved as a consequence of their inputs. Verbatim comments from reviewers, and our responses to these (in block yellow) are given below. We look forward to hearing from Nat Comms in the near future.

REVIEWER 1

Major issues

1- To avoid misinterpretations inherent of the Proteomics technique this study should be complemented with RNAseq analysis of the strain grown on fatty acids vs glucose. The stranded mRNA analysis could also help in the identification of the different TSS and promoters. The authors state in the discussion section that the PA0506, PA0507, PA0508 is not predicted to be an operon, but by RNAseq analysis they could easily check this fact.

Given the focus of this manuscript on the identification and characterization of the FadE1 and FadE2 proteins, proteomics is the optimal approach for these purposes. Modern quantitative proteomics is no more prone to misinterpretation than RNAseq. Moreover, we do not feel that inclusion of RNAseq analyses *in this paper* would add much. We do have extensive RNAseq data on *P. aeruginosa* grown in artificial sputum medium (again, data not intended for publication *in this paper*) which is rich in fatty acid substrates. The reads for the region PA0506-PA0507-PA0508 are shown below. It is clear from these data that PA0506 and PA0508 are both expressed in this medium, but as separate transcriptional units. PA0507 is not expressed to any appreciable extent.

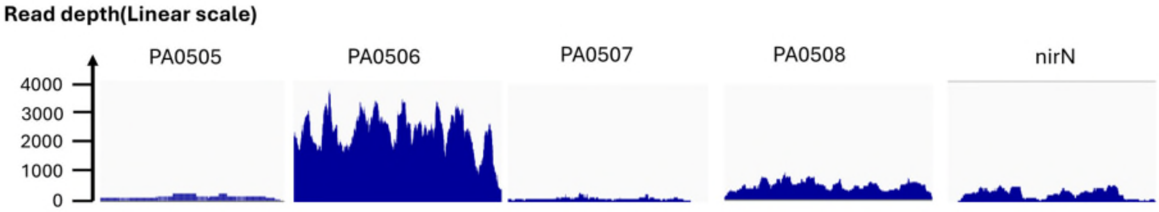

WT mono replicate 1

Gene name	Mean depth
PA0506	2882.44
PA0507	36.6839
PA0508	602.422

2- There is an ample discussion in the article about the possible role of PsrA and PvrA in regulating FadE1 and FadE2 expression. The presence of the consensus sequences of PsrA and PvrA binding sites in the promoter region of the genes coding for these dehydrogenases should be analyzed in silico or even in vitro by EMSA or in vivo by transcriptomic or proteomic analysis of PsrA or PvrA P. aeruginosa mutant strains.

As noted above, *this paper* is not focused on transcriptional regulation, although it would have been remiss of us to not mention this in the Discussion. *In silico* analyses did indeed reveal the presence of PsrA and PvrA binding motifs upstream of the *fadE1* and *fadE2* ORFs, and this is now acknowledged at the appropriate point in the Discussion (lines 467-480).

Other issues.

-Page 6. Line 136. Two FadE1 and FadE2 share 53% identity with one another, but almost none with E. coli FadE. Please show the identity percentage between E. coli FadE and FadE1 and FadE2.

The % identity of FadE1 and FadE2 with *E.coli* FadE is now stated (lines 148-149).

-Page 8. Line 180. "Other FadE homologs were upregulated …" It would be good to see the MSA of these FadE proteins with FadE1 and FadE2, highlighting the differences and even predicting the fatty acids used in each case. In fact a MSA and classification of all the 22 identified acyl-CoA dehydrogenases in *P aeruginosa* could be helpful to understand their differential role.

We generated the MSA as suggested by the referee, but inspection of the output led us to conclude that it did not tell us anything informative about the potential function of the 22 orthologues of FadE present in *P. aeruginosa*. Indeed, the level of sequence conservation in some cases was so low that the "catalytic glutamate" residue, which we know is essential for acyl-CoA dehydrogenase activity, was not correctly aligned. We note that a phylogenetic tree incorporating all 22 orthologs (and other relevant FadE-like enzymes from other organisms) is already shown in Fig S12. In summary, we investigated this suggestion, but felt that overall, it detracted from the main message of the work, rather than adding to it.

-Page 11. When comparing FadE1 and FadE2 structures between each other and with other homologs, Cα RMSD should be provided.

Done (lines 252-253).

-Page 13. FadE1^{E441A}bound to C16-CoA structure should also be compared with wtFadE1 (presumably bound to octanoyl-CoA) structure.

Done (see legend to Figure S9).

-Page 15. Line 306. Is there any extra electron density in the active site of FadE2 triple mutant?

No.

-In general, the quality of the figures describing the structures should be improved.

Figure quality has now been improved.

REVIEWER 2

Major points

1. Since the authors used tandem mass tags (TMT-16plex) approach for protein quantification, it is important to evaluate the labeling efficiency. Can authors provide this information? Also, please provide sample information for each tag channel.

The calculated N-terminal labelling efficiency was 92.2%. The percentage of TMT-labelled lysines was 99%. The overall labelling efficiency was 99.6%. This is now stated in the Materials and Methods (SI information section "TMT labelling…") The sample information for each tag channel is now given in Table S3 (SI information).

2. Further, they mentioned, "Sixteen pairs of dried fractions were analyzed by LC-MS/MS as previously described [6]…" How did they derive these 16 pairs of dried fractions? I could not obtain this information from reference [6] provided: Dolan Stephen K, Kohlstedt M, Trigg S, Vallejo Ramirez P, Kaminski Clemens F, Wittmann C, et al. Contextual Flexibility in Pseudomonas aeruginosa Central Carbon Metabolism during Growth in Single Carbon Sources. mBio. 2020;11:e02684-19. Though I got this information from the "Sample Processing Protocol" from the PRIDE database as follows: "Desalted peptides were dried and resuspended in 100 uL of 20mM ammonium formate (pH 10.0) then mixed with 4% (v/v) acetonitrile for fractionation. Fractionation was performed using Waters Acquity UPLC (Waters Corporation, Milford MA) equipped with an Acquity bridged ethyl hybrid C18 UPLC column (Waters; 2.1 mm i.d. x 150 mm, 1.7 µm particle size). Peptides were profiled at a flow rate of 0.25 mL/min with a linear gradient of 5-60% acetonitrile with 20 mM ammonium formate (pH 10.0) over 60 minutes. Thirty-two fractions were collected and reduced to dryness by vacuum centrifugation. Fractions were combined into 16 pairs (i.e. the first fraction with the middle fraction etc) and were analyzed by LC-MS/MS", this should be included in the method session. The authors should have described the protocol early in the supporting information. Furthermore, they should describe clearly how they have concatenated the fractions, such as combining fractions 1 and 17, 2 and 18, etc.

Yes, we overlooked this description. All relevant details are now supplied in the Materials and Methods (SI information section "LC-MS/MS…..).

3. Certain parameters used in this work and that of reference [6] were slightly different, such as the flow rate of the basic reverse phase run. This should have been specified for the sake of reporting accuracy.

Well spotted. We have now updated the Materials and Methods accordingly (in SI information section "LC-MS/MS…..).

4. I did not find the experimental details of LC-MS/MS analysis and data processing. These are crucial for evaluating the reliability and data quality of proteomic analysis. Specific details about the instrument parameters, such as the column type, gradient, flow rate, and MS settings (e.g., resolution, scan range, collision energy), are required to ensure reproducibility. How many sequence queries are in your database, and where does your database come from? How is the data distribution with or without

normalization? How does normalization proceed? How did the authors calculate the protein abundance using a fractionation dataset? Which software and search parameters did authors use for data processing? For example, the tolerance for MS1, MS2, and MS3, the fixed and variable modifications, the FDR at PSM, peptide, and protein level of target-decoy database searching, the number of missed cleavages, and the criteria of minimum peptide length should all be disclosed.

We have now updated the Materials and Methods accordingly (in SI information section "LC-MS/MS….." and "Data analysis").

5. On page 6: "Of the 2640 proteins that were common to all of the samples, 19 showed increased abundance (log2FC ≥ 1.0, adjusted p-value ≤ 0.01) on all three fatty acid substrates (cf. glucose) and 57 showed decreased abundance (Figure S2A, B)". Does this mean the author had ensured that all replicates have valid values? Any protein that has a missing value in even one replicate is discarded without applying imputation? Isn't that too stringent? Would that result in the accidental removal of any of the other 19 predicted acyl-CoA dehydrogenases (FadE) that might have been differentially expressed from this dataset?

Yes; all replicates have valid values as the proteins with any missing values (even in one replicate) were removed before normalization. This meant that one putative acyl-CoA dehydrogenase, PA1535(Q9I3H8), was discarded during data processing. We found no evidence to suggest that PA1535 is expressed at all in these growth conditions, so this is not an issue.

6. It is unclear what the input of PCA in Figure S2C is: the total proteome or the significantly changed proteins. The meaning is quite different based on the input dataset. Besides, there is no related description in the manuscript.

The PCA was generated from the total proteome, inclusive of the proteins common to all samples. This is now explicitly stated in the legend to Figure S2.

7. Figure 1A-C: There were other predicted proteins that were overexpressed in C8:0 (PA4435 & PA1631), C16:0 (PA4435) and C18d (PA4435). Are any of these proteins related to fatty acid metabolism?

Some possibly, some almost certainly not. Based on the phylogenetic tree in Figure S12, PA4435 is a member of the same clade as a human isovaleryl-CoA dehydrogenase and an unusual oxidase flavoprotein heterotetramer (the Fic32/33 complex from *S. ficellus*). Isovaleryl-CoA dehydrogenase is involved in leucine catabolism, and the Fic32/33 complex is involved in the NRPS-mediated synthesis of 1-azabicyclo[3.1.0]hexane. *It is therefore unlikely that PA4435 is involved in fatty acid catabolism, but rather, plays a role in the metabolism of amino acids or secondary metabolites*.

However, PA1631 is sandwiched between a clade that includes human ACAD-9 and a clade that includes several mammalian medium-chain acyl-CoA dehydrogenases. *It is therefore formally possible that PA1631 plays some role in medium-chain fatty acid metabolism*. However, we note that FadE2 (determined in this study as being the main medium-chain fatty acyl-CoA dehydrogenase involved in β-oxidation) was 22-fold up-

regulated on C8 substrate (*cf.* glucose) whereas PA1631 was <4-fold up-regulated on this substrate. It therefore seems likely that if PA1631 does play a role in fatty acid catabolism, it is a minor one (relative to FadE1 and FadE2).

Minor points:

1. On page S19, the authors mentioned using Bio-rad protein assay reagent. Is that Bradford assay or other types of assays for protein concentration estimation?

Yes. Bradford assay. This is now clarified (Methods section now on page S19 – last line of section entitled "sample preparation").

2. The authors should also preferably upload the protein FASTA used in the PRIDE repository and specify the version and date of download of such a database from UniProt.

The uploaded FASTA file is now included as an SI dataset. We have also updated the details in Method section accordingly.

3. For subsequent statistical analysis, the authors mentioned, "Following global normalization of the data (using common quality thresholds), the data were analyzed using limma with a mean reference model and the lmFit and Bayes functions." This information is too simplified. The authors should have included the parameters or algorithms used. For example, how did they pre-clean, impute, or normalize their data? Is there any minimal number of missing values?

We have updated the "Data Analysis" (paragraph 2) section accordingly (page S21), and the revised text should be much clearer.

4. To be reader-friendly, the proteins involved in glucose or fatty acid metabolism in Fig1A-1D should be labeled with a different color or symbol.

Done (relevant proteins boxed and labelled). Note that this is now Figure 2.

REVIEWER 3

Major comments:

1. In vivo validation of the enzymatic analysis. Measurement of the CoA pools via metabolomics is necessary to confirm the function of the putative acyl-CoA dehydrogenases in vivo. If the identities are correct, then the mutants (fadE1 and fadE2 KOs) will alter the CoA pools and provide an explanation for the phenotypes – toxicity from CoA sequestration.

We tried to address this, but soon ran into a problem: the growth rates of the *fadE1* single mutant and of the *fadE1 fadE2* double mutant on long chain fatty acid substrates were always low *cf*. the growth rate of the *fadE2* mutant or wild type (this is also reflected by the growth data in Fig 3 of the paper). To try and circumvent this, we also grew the strains on mixtures of long- and short-chain fatty acids; however, this did not relieve the growth defect in the *fadE1* mutant or the the *fadE1 fadE2* double mutant, suggesting that growth inhibition is probably not related to dysfunctional CoA sequestration *per se*. Moreover, growth rate effects are an issue because – as our metabolomics colleagues reminded us – large differences in growth rate have a direct impact on the metabolome, making it difficult to draw any biologically-relevant clear conclusions.

2. Biological validation of the *in silico* analysis. FadE1 and FadE2 biochemical inhibition by the thioester inhibitors is primarily needed to confirm the enzymes are sensitive to the inhibitors, and secondarily to validate their homolog selectivity. More importantly, drug (or perhaps prodrug) toxicity to *Pseudomonas aeruginosa* must be demonstrated to establish the viability for this antimicrobial strategy.

To address this, we collaborated with colleagues in the Chemistry Department (who are now authors on the revised MS) to synthesize the "hits" in our *in silico* analyses (compounds E and H) and their respective CoA derivatives. The CoA derivative of compound E inhibited neither FadE1 nor FadE2, but the CoA derivative of compound H was a very effective inhibitor of both enzymes (showing slightly greater inhibitory activity against FadE1, consistent with the *in silico* predictions). The data, validating the *in silico* analyses, are now shown in Figure 7 and described on lines 411-422. However, compound H did not inhibit the growth of *P. aeruginosa* (on a C8 carbon source) even when supplied at mM concentrations. This suggests either that it is not efficiently taken up by the bacteria, or that it is not efficiently converted to a CoA derivative once taken up. Nevertheless, our data confirm proof-of-principle that FadE1 and FadE2 can be targeted for inhibition by xenobiotics.

3. Infection phenotype for mutants. There is no evidence that inhibiting PA0506 and/or PA0508 will have any impact on Pseudomonas aeruginosa infection. Although there is a strong phenotype when the cells are grown in vitro, it is unclear how the other 20+ putative acyl-CoA dehydrogenases compensate (or don't) in the context of infection. Their potential redundant functions may be induced under conditions not tested in this study. Thus, whether inhibition of these enzymes have any therapeutic promise for this infection is uncertain.

To assess the impact of FadE1 and FadE2 on infection, we collaborated with the Galleria Infection Group at the University of Exeter (who are now authors on the revised MS). The Galleria haemocoel is rich in fatty acids, making this a good (3Rs-compliant) potential model for soft tissue infections generally. As shown in Figure 4, the *fadE1* mutant and the the *fadE1 fadE2* double mutant showed impaired infection (*cf*. the parental progenitor strain and the *fadE2* mutant). Note that, due to the lethality of PA14 in this infection model, we had to recapitulate the mutations in the less virulent PAO1 background. This is made clear in the body text. We conclude that loss of *fadE1* function does indeed lead to impaired soft tissue infection.

Minor comments:

1. A schematic of the β-oxidation process that illustrates where FadE fits in would benefit the reader and nonexpert who is unfamiliar with the reactions.

Done. This is now shown as Figure 1.

2. Can the differences between FadE1 and FadE2 be used to predict the substrate selectivity of the other 20+ *Pseudomonas aeruginosa* acyl-CoA dehydrogenases?

No, and although this is a nice idea, it goes well beyond the current study. Although we can change the specificities of FadE1 and FadE2 by e.g., switching the “filter” residues in their substrate tunnels, in doing this we also lose catalytic power (presumably by subtly perturbing the architecture of the active site). Clearly, the precise geometry of the active site is crucial, and there is much more to the problem than simply identifying a handful of potential specificity-determining residues. Moreover, even when potentially important residues are identified (e.g., the glutamine residue at the “pinch point” of FadE1 and FadE2 highlighted in the current study) this does not mean that their orientation in the fully-folded 3D structure is the same in different enzymes.

3. How many of the other putative acyl-CoA dehydrogenases are in gene clusters (e.g., polyketide synthesis)? Presumably this would suggest they are not primarily engaged in β-oxidation and not redundant for FadE1 or FadE2.

Seven of the putative acyl-CoA dehydrogenases encoded by *P. aeruginosa* PAO1 are located within a predicted operon or gene cluster. Not all of these have a reasonable identifiable likely function, but, based on the surrounding ORFs/genes, we predict that;

(i) PA0746 likely encodes an acyl-CoA dehydrogenase involved in alcohol metabolism catalysing the α-β dehydrogenation of certain intermediates.
(ii) PA5187 likely encodes an enzyme involved in a minor β-oxidation pathway.
(iii) PA0879 may be involved in steroid biosynthesis or the synthesis of cyclic carboxylate compounds.

However, given that the main focus of the paper is on FadE1 and FadE2, we would prefer not to speculate on their role(s) in this manuscript.

4. Figure 1. The genes of interest should be emphasized (bolded?) to better draw the reader’s attention to them.

Done.

5. An explanation must be provided for rationalizing the resolutions of the crystal structures and why the authors avoided statistically sensible cutoffs for resolution (e.g., I/sigma = 2). Although there is good multiplicity, completeness, and satisfactory CC1/2 in the highest shell, the R-factors for data at "this resolution" are unacceptable, particularly for the Apo FadE2 structure. The separation of the Rwork and Rfree for Apo FadE2 indicates over refinement and 1.94 Å resolution is unliklely. The data should be reprocessed at a lower resolution.

Our X-ray crystallography collaborator (and author on the MS), Dr Brear, addressed this for us. Although traditionally R-factors were used to determine the high resolution limit, it is now widely regarded as a being an unsuitable statistic to use for this purpose and leads to a significant amount of useful data being excluded. R-factors are highly dependent on the redundancy of the data, which is the number of times a reflection is measured. Higher redundancy can artificially inflate R-factors values even if the data quality is good. It therefore should not be used for this purpose. R-factors tends to increase non-linearly with resolution because higher resolution shells generally contain weaker reflections with lower signal-to-noise ratios. This natural increase in R-factors at higher resolutions can lead to an overly conservative cutoff, excluding potentially useful data. R-factors do not provide information about the actual signal-to-noise ratio (SNR) of the reflections, which is crucial for determining the true quality and utility of the data. CC1/2 is now seen as a better method for assessing the quality of the data and has replaced the use of both Rmerge/Rmeus/Rpim and I/σI (Karplus and Diederichs 2012, Karplus and Diederichs 2015). As can be seen from the data collection statistics, the CC1/2 are significantly above 0.1 in the higher resolution shell, which is regarded by many as a suitable cutoff to use (Karplus and Diederichs 2015). The separation for R-work to R-free for Apo FadE2 is within acceptable levels.

References cited.

Karplus, P. A. and K. Diederichs (2012). "Linking Crystallographic Model and Data Quality." Science **336**(6084): 1030-1033.

Karplus, P. A. and K. Diederichs (2015). "Assessing and maximizing data quality in macromolecular crystallography." Current Opinion in Structural Biology **34**: 60-68.
